# Asymmetric tandem conjugate addition and reaction with carbocations on acylimidazole Michael acceptors

**DOI:** 10.3762/bjoc.19.65

**Published:** 2023-06-16

**Authors:** Brigita Mudráková, Renata Marcia de Figueiredo, Jean-Marc Campagne, Radovan Šebesta

**Affiliations:** 1 Comenius University Bratislava, Faculty of Natural Sciences, Department of Organic Chemistry, Mlynská dolina, Ilkovičova 6, 842 15 Bratislava, Slovakia; 2 ICGM, University of Montpellier, CNRS, ENSCM, Montpellier, France

**Keywords:** acylimidazole, asymmetric catalysis, carbocation, conjugate addition, enolate

## Abstract

We present here a stereoselective tandem reaction based on the asymmetric conjugate addition of dialkylzinc reagents to unsaturated acylimidazoles followed by trapping of the intermediate zinc enolate with carbocations. The use of a chiral NHC ligand provides chiral zinc enolates in high enantiomeric purities. These enolates are reacted with highly electrophilic onium compounds to afford densely substituted acylimidazoles. DFT calculations helped to understand the reactivity of the zinc enolates derived from acylimidazoles and allowed their comparison with metal enolates obtained by other conjugate addition reactions.

## Introduction

Asymmetric metal-catalyzed conjugate additions provide access to numerous chiral scaffolds. This type of C–C bond formation efficiently enables the construction of stereogenic centers using polar organometallics [1]. In this way, 1,4-additions of typical organometallics such as dialkylzinc, Grignard reagents, and trialkylaluminum have been developed [2–9]. Recently, also Cu-catalyzed conjugate additions of organozirconium [10–11] or organoboron reagents were realized [12]. Also, in terms of suitable Michael acceptors as substrates, unsaturated ketones, aldehydes, esters, thioesters, amides, alkenyl heterocycles and enoyl heterocycles became viable for conjugate additions. The maturity and robustness of this methodology is documented by its applications in the total syntheses of complex natural products and other molecules of biological relevance [13–14].

Acylimidazoles proved to be versatile building blocks broadly applicable in asymmetric catalysis and organic synthesis. Today, acylimidazoles are used as ester/amide surrogates, because of their particular chemical and physical properties [15]. In addition to ester/amide synthesis, enoyl imidazolides were developed as excellent Michael acceptors. Acylimidazoles are unique electrophiles that demonstrate moderate reactivity, relatively high stability, chemical selectivity, and high solubility in water. Among exceptional properties belongs to easy post-transformation of acylimidazoles to common carbonyl analogs. These tunable properties allow the use of acylimidazoles in chemical biology research, which includes chemical synthesis of proteins/peptides, structure analysis, and functional control of RNA [16]. Moreover, Campagne and co-workers showed that Cu–NHC-catalyzed conjugate additions of dialkylzinc reagents proceed with high enantioselectivities [17–19]. Furthermore, this methodology allows iterative access to 1,3-disubstituted motifs that are present in various natural products [20].

A salient feature of conjugate additions of organometallic reagents is that they generate reactive metal enolates as primary products. These enolates can be used in a variety of subsequent transformations [21]. Chiral enolates generated by conjugate additions react with carbonyl compounds, imines, other Michael acceptors, or alkyl halides. Our group is developing trapping of metal enolates with stabilized carbocations and could show that magnesium enolates generated from enones [22], unsaturated amides [23], or heterocycles reacted with tropylium, dithiolylium or flavylium cations [24]. These non-traditional electrophiles allow access to structurally highly interesting motifs. In addition, they are amenable to valuable synthetic transformations such as oxidative ring contraction of the cycloheptatrienyl ring or reduction of the benzodithiolyl group.

**Scheme 1:**
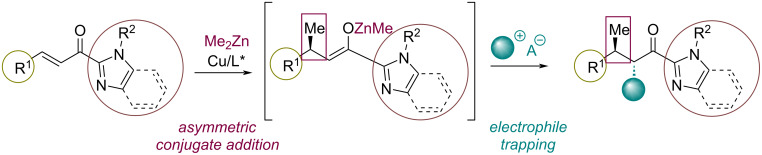
Concept of this work.

In this context, we decided to study a tandem reaction comprising the Cu–NHC-catalyzed addition of dialkylzinc reagents to enoyl imidazoles followed by a trapping reaction with various onium compounds (Scheme 1). In this work we show the development of this methodology and its application to a range of acylimidazoles and carbocations.

## Results and Discussion

**Scheme 2:**
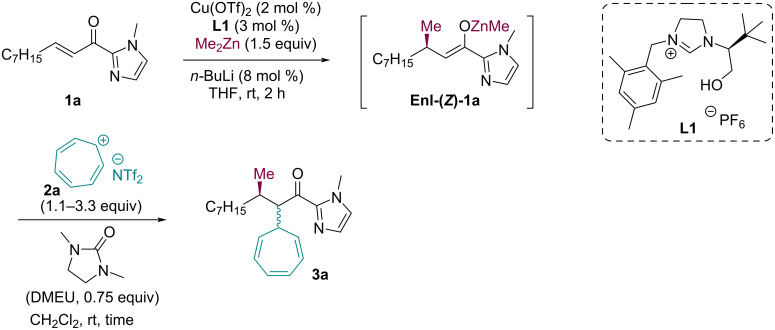
Initial experiments for the trapping of the intermediate enolate **Enl-1a** with tropylium NTf_2_.

For initial experiments, we have selected the conjugate addition of Me_2_Zn to acylimidazole **1a** catalyzed by a chiral NHC ligand derived from imidazolium salt **L1**. This NHC precursor has been described previously by Gérard, Mauduit, Campagne and co-workers [19]. The ligand **L1** is excellent in asymmetric conjugate additions of dialkylzincs to acylimidazoles [25]. The initial reaction conditions were inspired by literature precedence on conjugate additions. As the first electrophile for trapping of the chiral enolate, we have used tropylium bistriflimide (Scheme 2). Following our earlier experience, we employed the tropylium ion with the more lipophilic bis(trifluoromethane)sulfinimide (NTf_2_) anion because of its better solubility in the applied organic solvents than the commercially available tetrafluoroborate (BF_4_) form [22–24]. The cyclic urea DMEU (1,3-dimethyl-2-imidazolidinone) [26] additive was used as polar aprotic solvent, which also increases the homogeneity of the reaction mixture.

In the first reaction, 1.1 equiv of tropylium NTf_2_ were used. The reaction worked at room temperature and after 16 h the product **3a** was isolated in low 22% yield and a 2:1 diastereomeric ratio (Table 1, entry 1). First we attempted to increase the yield and the diastereoselectivity of the reaction by prolonging the reaction time (Table 1, entry 2). However, a shorter reaction time was preferable as after only one hour the product was isolated in a better yield (33%) with good dr (4:1, Table 1, entry 3). The addition of two equivalents of tropylium NTf_2_ led to a decrease of the diastereoselectivity (Table 1, entry 4). In the next tandem reaction, 1.1 equiv of tropylium bistriflimide (**2a**) were added to the reaction mixture every hour until the full conversion of the starting acylimidazole **3a** (Table 1, entry 5) as monitored by TLC analysis. The reaction was completed after 3 hours, meaning that the in situ-formed enolate needed 3.3 equiv of tropylium NTf_2_ (**2a**) to complete the reaction. By this route, the tandem product **3a** was isolated in a high yield of 93% but without any diastereoselectivity. The reaction was also carried out using 3.3 equiv of the electrophile added in one portion. The full conversion of the starting acylimidazole **1a** was observed after 30 minutes (TLC monitoring), however, the diastereoselectivity remained low (Table 1, entry 6). Neither an increase nor decrease of the reaction temperature led to improved reaction outcomes (Supporting Information File 1, Table S1, entries 2 and 11). We have continued the evaluation of reaction conditions for improving the diastereoselectivity of the reaction. We have tested transmetallation of the in situ-generated zinc enolate to the ammonium enolate by treatment with *n*-tetrabutylammonium chloride (Table 1, entry 7). For this purpose, the enolate was added to a solution of *n*-Bu_4_NCl in THF, and then the reaction mixture was stirred for 30 min before the addition of tropylium NTf_2_. Prolonging the transmetallation reaction time led to the formation of only one diastereomer, but in a low yield of 11%. Neither the addition of LiCl helped to increase the diastereoselectivity of the reaction (Table 1, entry 8). See Supporting Information File 1, for complete optimization of the reaction conditions.

**Table 1:** Results of selected optimization experiments.

Entry	Tropylium NTf_2_ [equiv]	Additive	Time [h]	Yield [%]	dr (*S,R*)/(*R,R*)-**3a**^a^

1	1.1	–	16	22	2:1
2	1.1	–	72	15	1:1
3	1.1	–	1	33	4:1
4	2.0	–	16	33	2:1
5^b^	3.3	–	3	93	1:1
6	3.3	–	0.5	94	1:1
7	3.3	*n*-Bu_4_N^+^Cl^−^	1	11	>99:1
8	3.3	LiCl	16	73	1:1

^a^The diastereoselectivity of the reaction was determined by ^1^H NMR spectroscopy of the crude reaction mixture; ^b^3 portions (1.1 equiv each) of tropylium NTf_2_ were added to the reaction mixture every one hour until the full conversion of **3a**.

Achieving diastereoselective reactions on acyclic systems is often difficult due to small energy differences between reacting conformers. In the case of our trapping reaction of chiral imidazolyl enolates, the lack of diastereoselectivity may be associated with the presence of an *E*/*Z* mixture of enolates. This would also explain why at lower onium salt amount and shorter reaction times the diastereoselectivities are higher but at the expense of the overall yield.

**Scheme 3:**
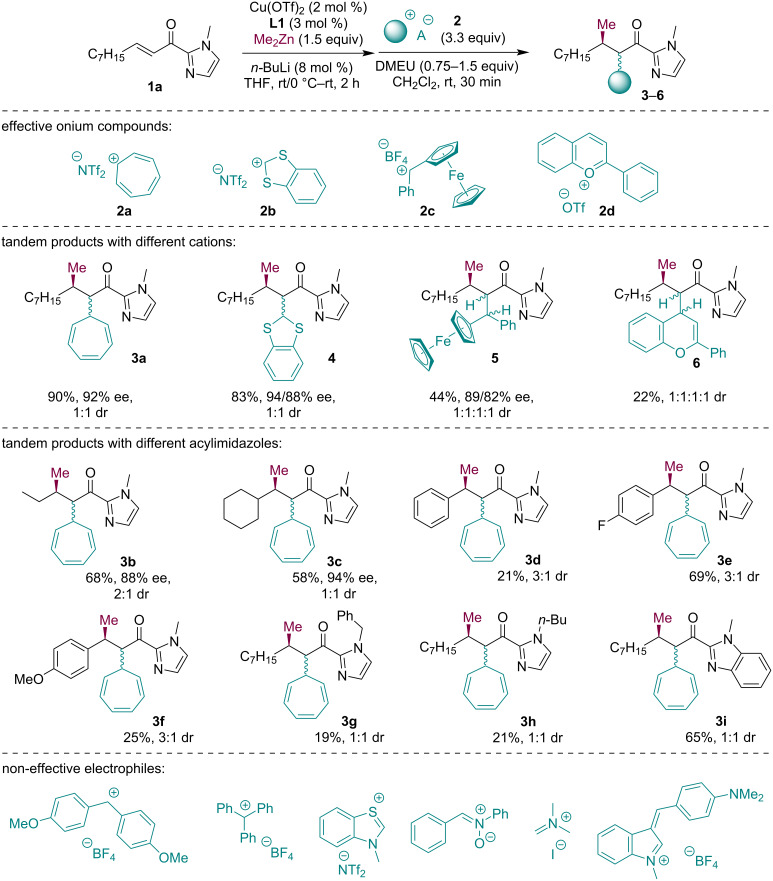
The reaction scope.

With the optimized reaction conditions in our hands, we next explored the scope of the domino reaction. Structurally diverse onium compounds **2** were tested to probe their reactivity with the Zn-enolate derived from **1a** (Scheme 3). The onium compounds **2a** and **2b** were obtained by anion exchange using LiNTf_2_ from the commercially available tetrafluoroborate salts. Onium compounds **2c** and **2d** were obtained by acidic dehydration of the corresponding hydroxy derivatives using HBF_4_ or CF_3_SO_3_H (see Supporting Information File 1 for more details). The corresponding tandem products were isolated in medium to good yields as mixtures of diastereomers and high enantiomeric purities were recorded for selected tandem products. The enantioselectivity of this reaction is mainly governed by the conjugate addition step and, in comparison to conjugate addition products described in the literature [19], these tandem products differed only slightly. So, we can assume that all tandem products have high enantiomeric purities.

**Figure 1:**
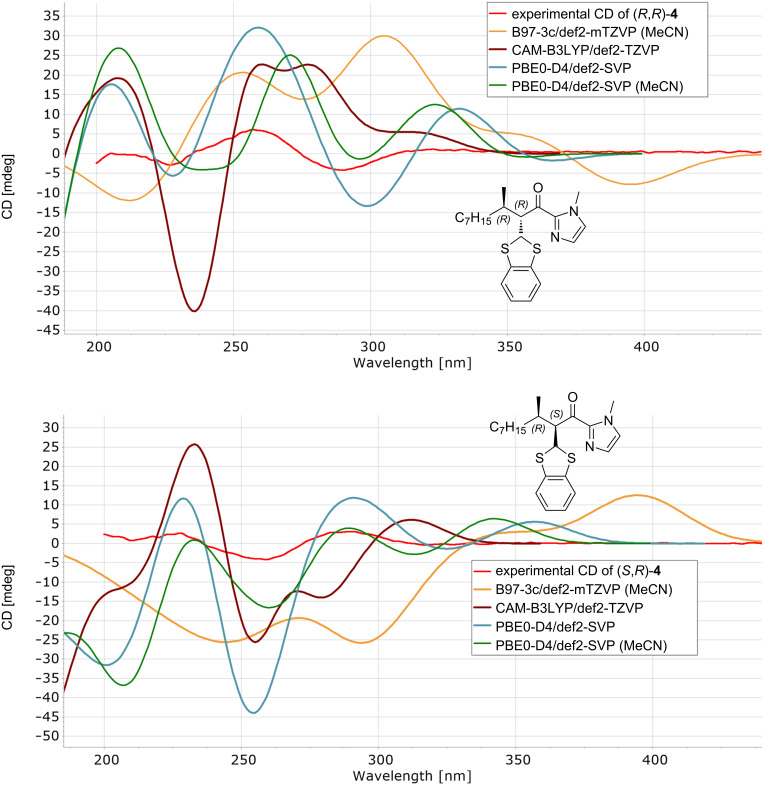
Comparison of DFT-calculated and experimental ECD of (2*R*,3*R*)-**4** and (2*S*,3*R*)-**4**.

Absolute configurations of tandem products were determined by comparison of experimentally measured electronic circular dichroism (ECD) spectra with those of the DFT-calculated ones (Figure 1 and see Supporting Information File 1 for more details). CD spectra were calculated for the two most populated conformers for both diastereomers of product **4**. The best match between the experimental and averaged calculated spectra was achieved by B97-3c/def2-mTZVP and PBE0-D4/def2-SVP methods. The presence of many conformers in these types of derivatives complicates their analysis and decreased the fit between experimental and calculated CD spectra. Furthermore, the configuration at the position C-3 is determined by the chiral ligand **L1** and was determined previously as (*R*) [19].

**Figure 2:**
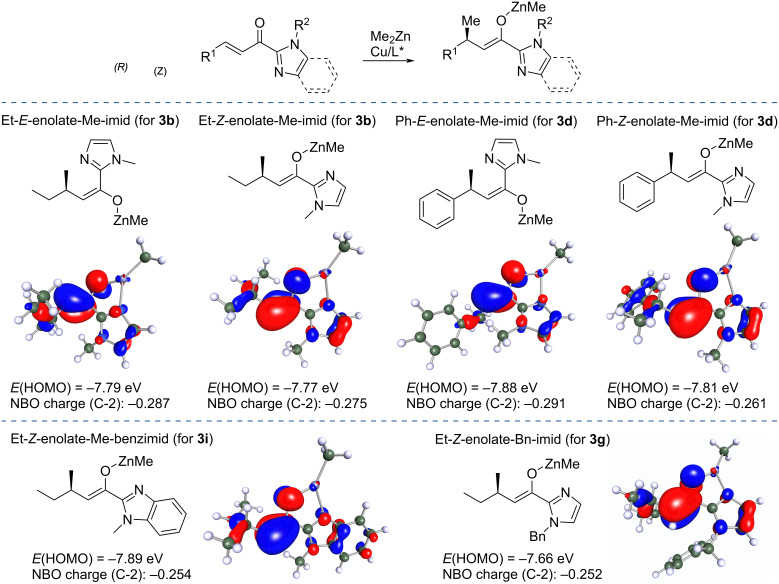
DFT calculated (ωB97X-D4/def2-TZVPPD//PBEh-3c/def2-mSVP) HOMO energies and NBO charges for representative metal enolates.

To gain insight into the reactivity of enolates formed in this transformation, we evaluated properties of Zn enolates by DFT calculations (Figure 2). The corresponding (*E*) and (*Z*)-enolates were calculated for products **3b** and **3d**, which possess either an alkyl (ethyl) or an aryl (phenyl) substituent on the stereogenic center. To probe the nature of the imidazole moiety, enolates for products **3g** and **3i** were also calculated (with a shorter alkyl chain to simplify the calculations). Single point energy calculations were performed using long-range corrected hybrid density functional ωB97X, which offer very good performance and have been recommended for general use in chemistry [27] with empirical dispersion correction D4 [28] (ωB97X-D4) and the triple-zeta def2-TZVPPD basis set [29]. Geometry optimizations were performed at the PBEh-3c/def2-mSVP level [30]. The energies of HOMOs show only small variations from −7.77 to −7.89 eV and the charges at the C-2 carbon obtained via natural population analysis also ranged only little from −0.254 to −0.291. Differences in HOMO energies and charges at C-2 of enolates cannot account for the differences in the yields of the trapping products as these are affected by their specific stabilities and issues during isolation and purification. As an additional question that we tried to answer with these calculations was the comparison of the Zn enolates obtained from acylimidazoles in this work with other metal enolates obtained by related conjugate additions. In comparison, the HOMO energy of a related silyl ketene aminal was similar (−7.87 eV) but its NBO charge was more negative −0.343. The TMS enolate of benzoxazole has a higher HOMO energy of −7.13 eV and an even more negative NBO charge of −0.368 at the C-2 position. We can confer from these data that Zn enolates obtained from acylimidazoles are somewhat less reactive than silyl enol ethers obtained in the Lewis acid-promoted conjugate addition of Grignard reagents [23]. This finding correlates also with the slightly lower yields for the tandem products obtained with Zn enolates from acylimidazoles.

## Conclusion

Enantioselective conjugate additions of dialkylzinc reagents afford chiral zinc enolates. These reactive species were trapped with several highly electrophilic onium compounds to introduce synthetically valuable functionalities. In this way, a range of acylimidazoles featuring cycloheptatrienyl, benzodithiolyl, ferrocenyl, and chromenyl substituents were prepared. The DFT calculated HOMO energies and NBO charges of the intermediate zinc enolates allowed placement of these reactive intermediates among other metal enolates obtained in conjugate additions.

## Experimental

**General procedure for the one-pot conjugate addition of organozinc reagents to acylimidazole followed by trapping with carbocations:** In a flame-dried Schlenk flask flushed with Ar, Cu(OTf)_2_ (1.81 mg, 0.005 mmol, 2 mol %) and chiral NHC ligand **L1** (3.36 mg, 0.0075 mmol, 3 mol %) were dissolved in freshly distilled anhydrous THF (1.0 mL) and the mixture was stirred for 10 min at rt. The reaction mixture was cooled to 0 °C, and then 1.6 M *n*-BuLi (12.5 µL, 0.02 mmol, 8 mol %) was added dropwise and the mixture was stirred for 10 min. Subsequently, 1.2 M dimethylzinc reagent in toluene (0.31 mL, 0.38 mmol, 1.5 equiv) was added dropwise to the solution and the resulting mixture was also stirred for 10 min. The acylimidazole (0.25 mmol, 1.0 equiv) dissolved in anhydrous THF (0.5 mL) was added dropwise to the mixture. The reaction was stirred for 2 h, while it was slowly warmed up to rt. Then, the electrophile in anhydrous CH_2_Cl_2_ (1.0 mL) together with DMEU (20.2–40.4 µL, 75–150 mol %, to achieve homogeneity of the reaction mixture) were added to the reaction mixture followed by stirring at rt for 0.5–1 h. The reaction was quenched by the addition of 1 M HCl (6 mL) and EtOAc (6 mL). Then, the organic phase was washed with a sat. aq. solution of NaHCO_3_ (6 mL), brine (6 mL), dried over MgSO_4_, and concentrated under vacuum. The residue was purified by flash chromatography on SiO_2_ (hexane/EtOAc 15:1).

## Supporting Information

File 1 Characterization data for all compounds, computational details, and picture of NMR spectra.
