# Supplementary material for: Asymmetric tandem conjugate addition and reaction with carbocations on acylimidazole Michael acceptors
Source: Beilstein J Org Chem. 2023 Jun 16;19:881–8. doi: 10.3762/bjoc.19.65 (PMC10280057; doi:10.3762/bjoc.19.65)
Supplement: File 1 — Characterization data for all compounds, computational details, and picture of NMR spectra. [file Beilstein_J_Org_Chem-19-881-s001.pdf]

BEILSTEIN JOURNAL OF ORGANIC CHEMISTRY

Supporting Information

for

Asymmetric tandem conjugate addition and reaction with carbocations on acylimidazole Michael acceptors

Brigita Mudráková, Renata Marcia de Figueiredo, Jean-Marc Campagne and Radovan Šebesta

Beilstein J. Org. Chem. **2023,** *19,* 881–888. doi:10.3762/bjoc.19.65

Characterization data for all compounds, computational details, and picture of NMR spectra

License and Terms: This is a supporting information file under the terms of the Creative Commons Attribution License (https://creativecommons.org/licenses/by/4.0). Please note that the reuse, redistribution and reproduction in particular requires that the author(s) and source are credited and that individual graphics may be subject to special legal provisions.
The license is subject to the *Beilstein Journal of Organic Chemistry* terms and conditions: (https://www.beilstein-journals.org/bjoc/terms)

Table of contents

1. General information S2
2. Additional experimental results S3
    2.1. Optimization of the tandem reaction conditions on acylimidazole derivative S3
    Table S1: Optimization of the tandem reaction conditions on acylimidazole **1a** with tropylium NTf_2 .... S3
    Table S2: Optimization of the tandem reaction conditions on acylimidazole **1a**, changing the zinc enolate to the ammonium salt, and reaction with tropylium NTf_2. ..... S4
    Table S3: Optimization of the tandem reaction conditions on acylimidazole **1a**, transmetallation of zinc enolate to the lithium enolate, and reaction with tropylium NTf_2. .... S4
3. Experimental procedures S5
4. Characterization data S9
5. Copies of NMR, MS, HRMS, and HPLC spectra S17
6. CD spectra S40
7. Details of DFT calculations S40
8. References S62

1. General information

The reactions were carried out under Ar atmosphere using oven-dried glassware and standard Schlenk techniques. Solvents were dried and purified by standard procedures described in the literature and were freshly distilled prior to their use [1]. CH_2Cl_2 was dried and distilled over calcium hydride, Et_2O, MTBE, THF were dried and distilled over Na/benzophenone. $Cu(OTf)_2$ was commercially available from Sigma-Aldrich. Ligand **L1** was prepared according to a literature procedure [2]. Organozinc reagents were commercially available from Acros. Acylimidazoles [3] and cations [4-6] were synthesized according to the literature. The prepared salts were stored under argon in the dark and in the freezer and were used up to a maximum of two weeks after their syntheses. Reactions were monitored by thin-layer chromatography using Merck silica gel 60 F_{254} glass TLC plates. Following the general work-up, products were purified by flash chromatography using Isolera Biotage FSKO-1107-0010 and Std, HC or HP Puriflash columns by Interchim. NMR spectra were measured on a Varian VNMRS 600 spectrometer (600 MHz for ^{1}H NMR, 150 MHz for ^{13}C NM, and 564 MHz for ^{19}F NMR) and a Bruker Avance NEO 400 MHz (400 MHz for ^{1}H NMR, 100 MHz for ^{13}C NMR, and 376 MHz for ^{19}F NMR). Chemical shifts (δ) in ^{1}H and ^{13}C NMR spectra are reported down-fielded to TMS as an internal standard and are given in ppm. ^{19}F NMR were measured using ref. sample CCl_3F. Interaction constants (*J*) are given in Hz. The following abbreviations are used to describe the multiplicity of the observed signals - s (singlet), d (doublet), t (triplet), q (quartet) and m (multiplet). High-resolution mass spectrometry was measured by using an Orbitrap Thermo Scientific Velos pro with HESI (heated electrospray ionization). Enantiomeric purities were determined by Chiralcel and Chiralpak (Daicel Chemical Industries Ltd.) and Chiral ART (YMC) columns on HPLC Agilent Technologies 1200 Infinity series using the Chemstation software for LC systems. Circular dichroism spectra were determined by using a Jasco J-815 spectrometer. IR spectra were recorded on an Agilent Technologies Cary 630 FTIR. Melting points were measured on an M-565 Büchi apparatus.

2. Additional experimental results

2.1. Optimization of the tandem reaction conditions on acylimidazole derivative

Table S1: Optimization of the tandem reaction conditions on acylimidazole **1a** with tropylium NTf_2.

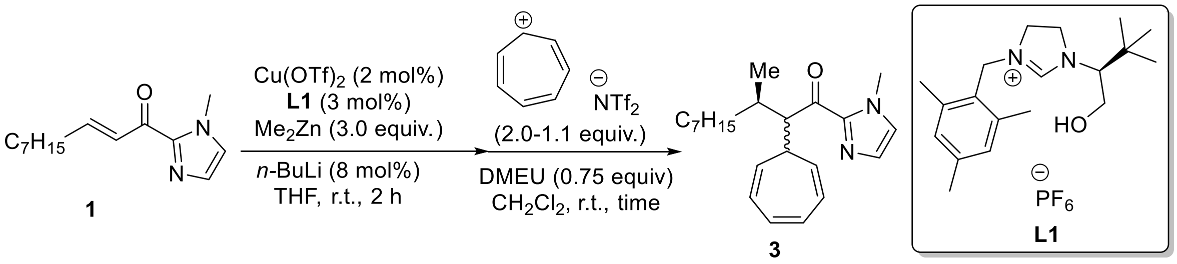

Entry	Trop. NTf_2 [equiv.]	Temp.	Time [h]	Yield [%]	dr[d]
1	1.1	r.t.	16	22	2:1
2	1.1	40°C	16	9	5:1
3	1.1	r.t.	72	15	1:1
4	1.1	r.t.	5	29	4:1
5	1.1	r.t.	2	32	4:1
6	1.1	r.t.	1	33	4:1
7	2.0	r.t.	16	33	2:1
8[a]	3.3	r.t.	3	93	1:1
9	3.3	r.t.	0.5	94	1:1
10[b]	3.3	r.t.	4	78	1:1
11	3.3	0°C	1	84	1:1
12[c]	3.3	r.t.	0.5	54	1:1
13	3.0	r.t.	0.5	61	1:1
14	2.5	r.t.	0.5	48	1:1

[a]3 portions (1.1 equiv of tropylium NTf_2 were added to the reaction mixture every hour until the full conversion). [b]Syringe pump (rate 0.7 mL/h, volume 1.5 mL of solution, 1.3 mL of CH_2Cl_2). [c]Tropylium tetrafluoroborate was used. [d]The diastereoselectivity of the reaction was determined by ^1H NMR spectroscopy of the crude reaction mixture.

Table S2: Optimization of the tandem reaction conditions on acylimidazole **1a**, changing the zinc enolate to the ammonium salt, and reaction with tropylium NTf_2.

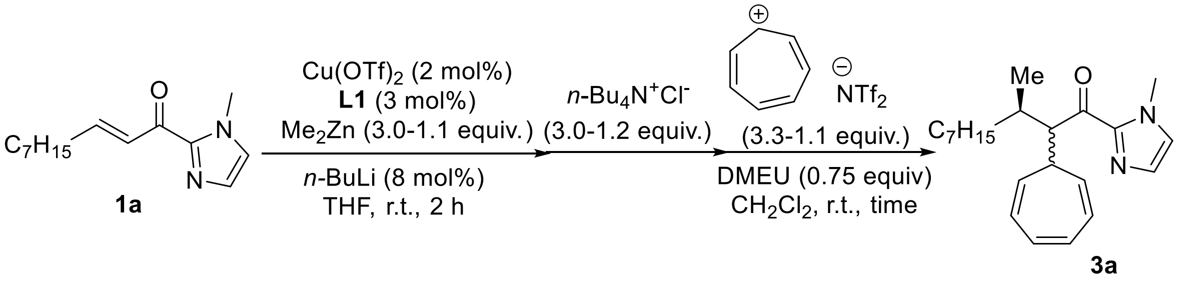

Entry	Me_2Zn [equiv.]	*n*-$Bu_4N^+Cl^-$ [equiv.]	Trop. NTf_2 [equiv.]	Time [h]	Yield [%]	dr[b]
1	3.0	3.0	1.1	16	20	8:1
2	1.5	1.2	3.3	0.5	44	2:1
3[a]	1.1	1.2	3.3	1	11	>99:1
4[a]	1.1	1.2	3.3	16	13	3:1

[a]The enolate was added to the solution of *n*-$Bu_4N^+Cl^-$ in THF, and then the reaction mixture was stirred 30 min before the addition of tropylium NTf_2. [b]The diastereoselectivity of the reaction was determined by ^1H NMR spectroscopy of the crude reaction mixture.

Table S3: Optimization of the tandem reaction conditions on acylimidazole **1a**, transmetallation of zinc enolate to the lithium enolate, and reaction with tropylium NTf_2.

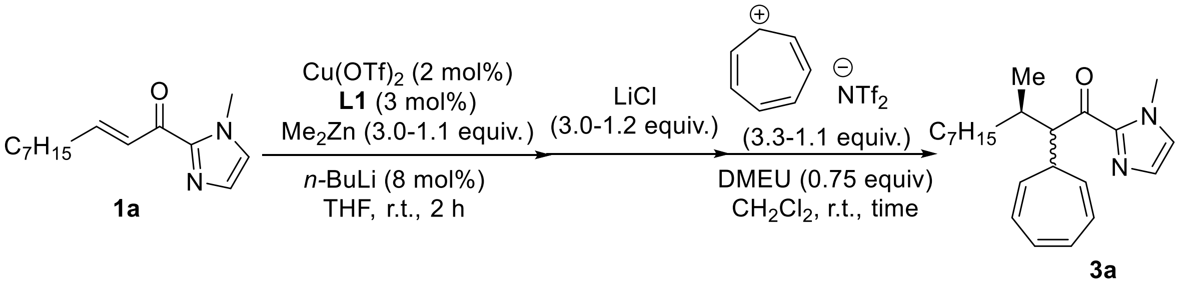

Entry	Me_2Zn [equiv.]	LiCl [equiv.]	Trop. NTf_2 [equiv.]	Time [h]	Yield [%]	dr[b]
1	3.0	3.0	1.1	16	25	5:1
2	1.5	1.2	3.3	0.5	76	1:1
3[a]	1.1	1.2	3.3	16	73	1:1
4[a]	1.1	1.2	3.3	0.5	56	1:1

[a]The enolate was added to the solution of LiCl in THF, and then the reaction mixture was stirred 30 min before the addition of tropylium NTf_2. [b]The diastereoselectivity of the reaction was determined by ^1H NMR spectroscopy of the crude reaction mixture.

3. Experimental procedures

Synthesis of the starting acylimidazoles

Method A

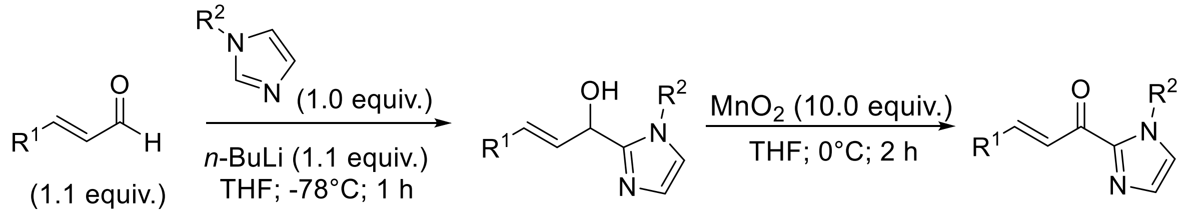

Synthesis of alcohols:

In a flame-dried round-bottomed flask, a 1.6 M solution of *n*-BuLi in hexane (8.38 mL, 13.40 mmol, 1.1 equiv) was added dropwise at −78 °C to a solution of NMI (0.97 mL, 12.20 mmol, 1.0 equiv) in anhydrous THF (24.4 mL). The resulting solution was stirred for 10 min at −78 °C, then allowed to warm to rt and stirred for additional 10 min. Afterwards, the reaction was cooled to −78 °C before the addition of the aldehyde (13.40 mmol, 1.1 equiv). The mixture was stirred for 1 h at −78 °C, then H_2O (10 mL) was added to quench the reaction. The organic phase was washed with a sat. aq. solution of $NaHCO_3$ (15 mL) and brine (15 mL). Then, the organic phase was dried over $MgSO_4$ and concentrated under vacuum. The desired alcohol was purified by column chromatography on SiO_2 (CH_2Cl_2/MeOH 96:4).

Synthesis of acylimidazoles:

In a flame-dried round-bottomed flask, the alcohol (10.30 mmol, 1.0 equiv) was taken up in anhydrous THF (103 mL; c = 0.1 M). MnO_2 (8.95 g, 103.00 mmol, 10.0 equiv) was added in one portion to the solution at 0 °C. The mixture was stirred for 2 h and monitored by TLC. After completion, the reaction mixture was allowed to warm to rt, filtered through a pad of celite, washed with EtOAc (150 mL), and the solvent was evaporated under reduced pressure. The product was purified by column chromatography on SiO_2 (pentane/EtOAc 4:6).

Method B

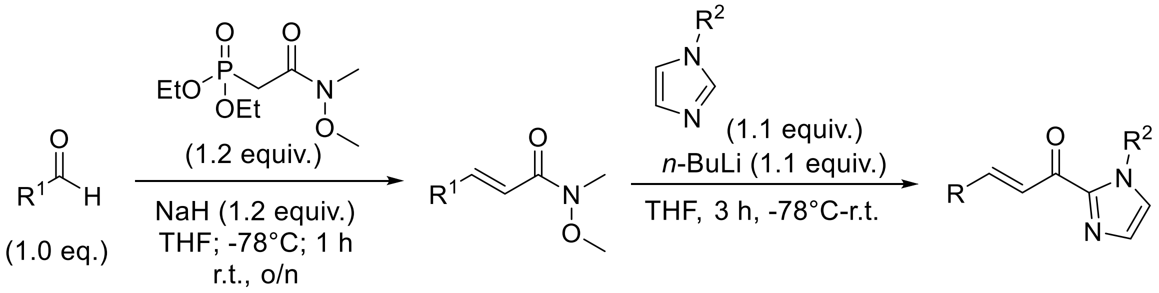

Synthesis of Weinreb amides:

In a flame-dried round-bottomed flask, diethyl 2-(methoxy(methyl)amino)-2-oxoethylphosphonate (2.87 g, 12.00 mmol, 1.2 equiv) was dissolved in anhydrous THF (25 mL) and sodium hydride (powder, 95%, 288.0 mg, 12.00 mmol, 1.2 equiv) was added portion-wise at rt until the bubbling has stopped. The mixture was then cooled to −78 °C before the addition of the aldehyde (10.00 mmol, 1.0 equiv) dissolved in anhydrous THF (5.8 mL). The reaction medium was stirred at −78 °C for 1 h, then allowed to warm to rt and stirred overnight. Then, water was added to quench the reaction. The layers were separated and the organic phase was washed with a sat. aq. solution of $NaHCO_3$ (15 mL), brine (15 mL), dried over $MgSO_4$, and concentrated under vacuum. The residue was purified by column chromatography on SiO_2 (pentane/EtOAc 4:6).

Synthesis of acylimidazoles:

In a flame-dried round-bottomed flask, a 1.6 M solution of *n*-BuLi in hexane (3.29 mL, 5.26 mmol, 1.1 equiv) was added dropwise at −78 °C to a solution of 1-methyl-1*H*-imidazole (0.41 mL, 5.26 mmol, 1.1 equiv) in anhydrous THF (14.5 mL). The resulting mixture was stirred for 20 min at −78 °C, and then was allowed to warm to rt and stirred for additional 10 min. The reaction mixture was cooled to −78 °C before the addition of the Weinreb amide (4.78 mmol, 1.0 equiv) in anhydrous THF (5.3 mL). The mixture was stirred for 30 min at −78 °C, then for 3 h at rt followed by the addition of a sat. aq. solution of NH_4Cl (15 mL) in order to quench the reaction. EtOAc (15 mL) was added and the organic phase was separated and further washed with a sat. aq. solution of $NaHCO_3$ (15 mL) and brine (15 mL). The organic layer was dried over $MgSO_4$ and evaporated. Purification was performed by column chromatography on SiO_2 (pentane/EtOAc 4:6).

Preparations of the electrophilic reactants for one-pot reactions

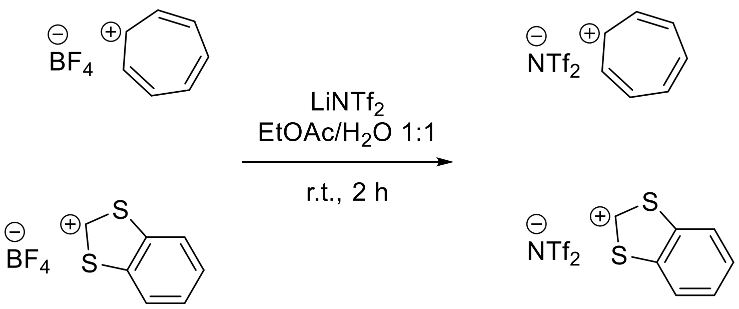

Tropylium BF_4 (1.0 g, 5.60 mmol, 1.0 equiv) or 1,3-benzodithiol-2-ylium BF_4 (1.34 g, 5.60 mmol, 1.0 equiv) and $LiNTf_2$ (1.61 g, 5.60 mmol, 1.0 equiv) were dissolved in a mixture of EtOAc (14 mL) and H_2O (14 mL) and stirred at room temperature for 2 h. Next, the phases were separated, and the organic phase was dried over anhydr. $MgSO_4$, filtered, and the solvent was evaporated under reduced pressure. The solids were dried under high vacuum.

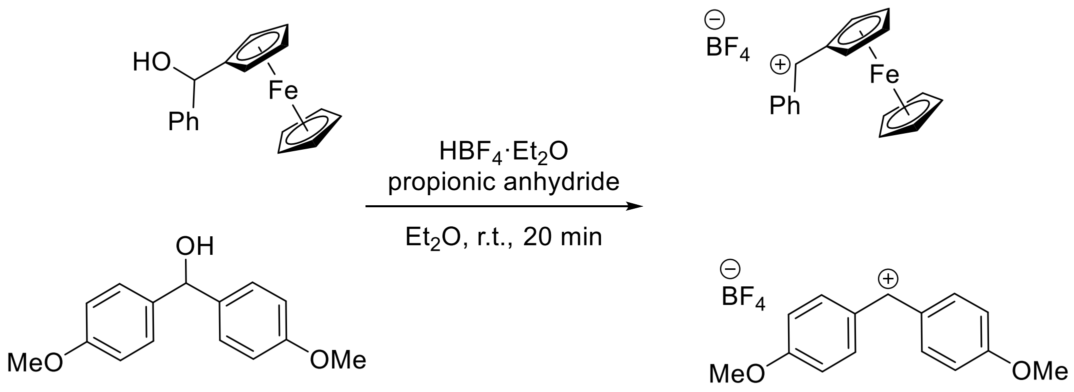

To a solution of 4,4'-dimethoxybenzhydrol (0.50 g, 2.00 mmol, 1.0 equiv) or (hydroxyphenylmethyl)ferrocene (0.56 g, 2.00 mmol, 1.0 equiv) in dry Et_2O (6 mL) was added propionic anhydride (1.3 mL, 10.2 mmol, 10.0 equiv). Next, $HBF_4{\cdot}Et_2O$ (0.58 mL, 4.10 mmol, 2.0 equiv) was dropwise added, and the resulting mixture was stirred for 20 min at room temperature. Then, the reaction mixture was cooled to 0 °C and stirred for 10 min. The resulting precipitate was filtered and washed with cold anhydrous Et_2O (3 × 10 mL) and pentane (3 × 10 mL). The solids were dried under high vacuum.

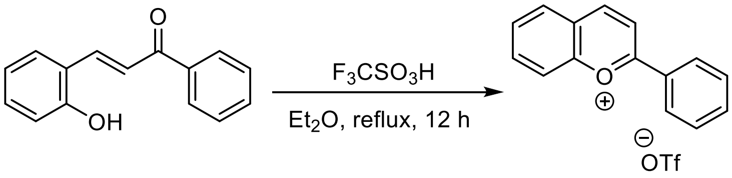

To a suspension of (*E*)-3-(2-hydroxyphenyl)-1-phenylprop-2-en-1-one (1.5 g, 6.69 mmol, 1.0 equiv) in Et_2O (4 mL) was added dropwise F_3CSO_3H (0.59 µL mL, 22.55 mmol, 3.4 equiv). The reaction mixture was refluxed overnight. Then, the mixture was filtered, the precipitate was washed with cold anhydrous Et_2O (3 × 5 mL), and the solid was dried under high vacuum.

General procedure for the one-pot conjugate addition of organozinc reagents to acylimidazole followed by trapping with carbocations

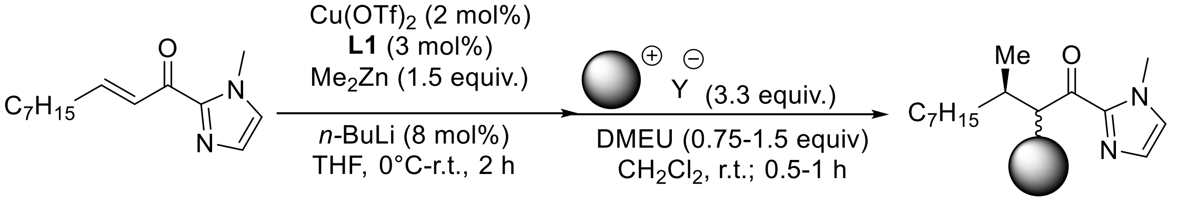

In a flame-dried Schlenk flask flushed with Ar, the copper source (1.81 mg, 0.005 mmol, 2 mol %) and chiral NHC ligand (3.36 mg, 0.0075 mmol, 3 mol %) were dissolved in freshly distilled anhydrous THF (1 mL) and the mixture was stirred for 10 min at rt. The reaction mixture was cooled to 0 °C, and then a 1.6 M solution of *n*-BuLi in hexane (12.5 µL, 0.02 mmol, 8 mol %) was added dropwise and the mixture was stirred for 10 min. Subsequently, a 1.2 M solution of dimethylzinc reagent in toluene (0.31 mL, 0.38 mmol, 1.5 equiv) was added dropwise to the solution and the resulting mixture was also stirred for 10 min. The acylimidazole (0.25 mmol, 1.0 equiv) in anhydrous THF (0.5 mL) was added dropwise to the mixture. The reaction mixture was stirred for 2 h, while it was slowly allowed to warm to rt. Then, the electrophile in anhydrous CH_2Cl_2 (1 mL) together with DMEU (20.2–40.4 µL, 75–150 mol %, to achieve homogeneity of the reaction mixture) were added to the reaction mixture and stirring at rt was continued for 0.5–1 h. The reaction was quenched with 1 M HCl (6 mL), and EtOAc (6 mL) was added. Then, the organic phase was washed with a sat. aq. solution of $NaHCO_3$ (6 mL), with brine (6 mL), dried over $MgSO_4$, and concentrated under vacuum. The residue was purified by flash chromatography on SiO_2 (hexane/EtOAc 15:1).

General procedure for the racemic one-pot conjugate addition of organozinc reagents to acylimidazole followed by trapping with carbocations

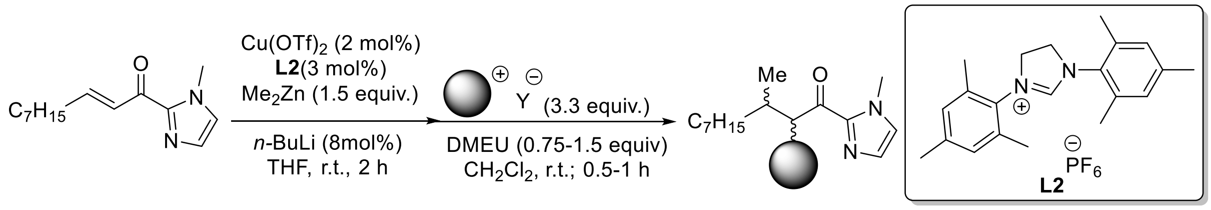

In a flame-dried Schlenk flask flushed with Ar, the copper source (1.81 mg, 0.005 mmol, 2 mol %) and achiral NHC ligand (3.60 mg, 0.0075 mmol, 3 mol %) were dissolved in freshly distilled anhydrous THF (1 mL). The mixture was stirred for 10 min at rt. Then, a 1.6 M solution of *n*-BuLi in hexane (12.5 µL, 0.02 mmol, 8 mol %) was added dropwise and the mixture was stirred also for 10 min. Subsequently, a 1.2 M solution of the dimethylzinc reagent in toluene (0.31 mL, 0.38 mmol, 1.5 equiv) was added dropwise to the solution. The resulting mixture was stirred at rt for 10 min. The acylimidazole (0.25 mmol, 1.0 equiv) in anhydrous THF (0.5 mL) was added dropwise and the reaction mixture was stirred at rt for 2 h. Next, the electrophile in anhydrous CH_2Cl_2 (1 mL) together with DMEU (20.2–40.4 µL, 75–150 mol %, to achieve homogeneity of the reaction mixture) were added to the reaction mixture and stirring at room temperature was continued 0.5–1 h. The reaction was quenched with 1 M HCl (6 mL), and EtOAc (6 mL) was added. Then, the organic phase washed with a sat. aq. solution of $NaHCO_3$ (6 mL), with brine (6 mL), dried over $MgSO_4$, and concentrated under vacuum. The residue was purified by flash chromatography on SiO_2 (hexane/EtOAc 15:1).

4. Characterization data

(3*R*)-2-(Cyclohepta-2,4,6-trien-1-yl)-3-methyl-1-(1-methyl-1*H*-imidazol-2-yl)decan-1-one (**3a**)

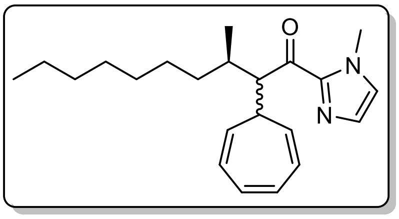

Orange oil, yield: 90% (76.6 mg), d. r. 1:1. purified by silica gel flash chromatography (hexane/EtOAc 8:1). The diastereomers were not separated. **^{1}H NMR** (400 MHz, $CDCl_3$): δ 7.17 (s, 1H), 7.16 (s, 1H), 7.03 (s, 2H), 6.68 – 6.61 (m, 4H), 6.23 – 6.05 (m, 4H), 5.36 (dd, *J* = 9.3, 5.9 Hz, 1H), 5.31 – 5.23 (m, 3H), 4.46 (dd, *J* = 10.0, 5.6 Hz, 1H), 4.38 (dd, *J* = 8.7, 6.9 Hz, 1H), 3.99 (s, 3H), 3.98 (s, 3H), 2.29 – 2.13 (m, 2H), 2.12 – 2.00 (m, 2H), 1.51 – 1.03 (m, 24H), 0.91 (d, *J* = 6.9 Hz, 3H), 0.88 – 0.82 (m, 9H) ppm. **^{13}C NMR** (151 MHz, $CDCl_3$): δ 196.6, 196.4, 145.0, 144.8, 130.9, 130.8, 130.5, 130.5, 129.1, 129.0, 127.2 (2 C), 124.7 (2 C), 124.6, 124.6, 123.8, 123.5, 123.0, 122.4, 52.2, 51.2, 39.8, 39.6,

36.4, 36.4 (2 C), 36.4, 35.1, 34.5, 34.4, 33.7, 31.8, 29.6, 29.2, 29.2, 27.3, 27.2, 22.6 (2 C), 17.5, 15.7, 14.1 (2 C) ppm. **IR** (ATR): 3106, 3010, 2954, 2924, 2853, 2068, 1988, 1662, 1457, 1406, 1364, 1156, 913, 768, 744, 709 cm^{-1}. **HRMS** (HESI): *m/z* $[M+Na]^+$ calcd for $C_{22}H_{32}N_2ONa$: 363.2407, found: 363.2407. **HPLC**: Chiralpak IC, hexane/*i*-PrOH = 90:10, 1.0 mL/min, 260 nm, t_R(minor) = 5.25 min, t_R(major) = 5.52 min.

(3*R*)-2-(Benzo[*d*][1,3]dithiol-2-yl)-3-methyl-1-(1-methyl-1*H*-imidazol-2-yl)decan-1-one (**4**)

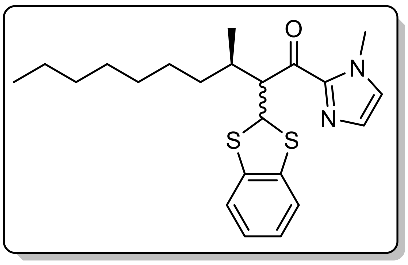

Yellow oil, yield: 83% (84.0 mg), diastereoselective ratio 1:1, purified by silica gel flash chromatography (hexane/EtOAc 15:1). The diastereomers were separated by flash chromatography. **^{1}H NMR** (**4-diast1**) (400 MHz, $CDCl_3$): δ 7.18 – 7.15 (m, 1H), 7.11 – 7.08 (m, 2H), 7.01 (s, 1H), 6.99 – 6.95 (m, 2H), 5.37 (d, *J* = 10.6 Hz, 1H), 4.55 (dd, *J* = 10.6, 4.4 Hz, 1H), 3.95 (s, 3H), 2.28 – 2.19 (m, 1H), 1.53 – 1.41 (m, 1H), 1.37 – 1.16 (m, 10H), 1.03 – 0.93 (m, 1H), 0.90 (d, *J* = 6.9 Hz, 3H), 0.85 (t, *J* = 6.9 Hz, 3H) ppm. **^{1}H NMR** (**4-diast2**) (400 MHz, $CDCl_3$) δ 7.15 – 7.08 (m, 3H), 7.00 (s, 1H), 6.98 – 6.94 (m, 2H), 5.41 (d, *J* = 10.0 Hz, 1H), 4.51 (dd, *J* = 10.0, 5.0 Hz, 1H), 3.90 (s, 3H), 2.28 – 2.16 (m, 1H), 1.57 – 1.45 (m, 1H), 1.42 – 1.16 (m, 10H), 1.15 – 1.04 (m, 1H), 0.95 (d, *J* = 7.0 Hz, 3H), 0.87 (t, *J* = 6.8 Hz, 3H) ppm. **^{13}C NMR** (**4-diast1**) (151 MHz, $CDCl_3$): δ 193.59, 144.17, 137.59, 136.87, 129.25, 127.26, 125.48, 125.37, 122.47, 122.35, 56.91, 54.42, 36.29, 35.33, 34.82, 31.81, 29.50, 29.19, 27.31, 22.62, 15.15, 14.08 ppm. **^{13}C NMR** (**4-diast2**) (151 MHz, $CDCl_3$) δ 193.41, 144.04, 137.70, 137.08, 129.23, 127.24, 125.46, 125.34, 122.44, 122.27, 57.56, 54.15, 36.25, 34.61, 32.56, 31.83, 29.60, 29.26, 27.48, 22.64, 17.95, 14.10 ppm. **IR** (**4-diast1**) (ATR): 3110, 2922, 2852, 2111, 1659, 1444, 1406, 1117, 911, 739, 687 cm^{-1}. **IR** (**4-diast2**) (ATR): 3105, 2923, 2853, 1660, 1444, 1406, 1117, 987, 913, 739 cm^{-1}. **HRMS** (HESI, **4-diast1**): *m/z* $[M+Na]^+$ calcd for $C_{22}H_{30}N_2S_2ONa$: 425.1692, found: 425.1692. **HRMS** (HESI, **4-diast2**): *m/z* $[M+Na]^+$ calcd for $C_{22}H_{30}N_2S_2ONa$: 425.1692, found: 425.1692. **HPLC** (**4-diast1**): Chiralpak IC, hexane/*i*-PrOH = 95:5, 1.0 mL/min, 260 nm, t_R(major) = 6.491 min, t_R(minor) = 9.242 min. **HPLC** (**4-diast2**): Chiralpak IC, hexane/i-PrOH = 95:5, 1.0 mL/min, 260 nm, t_R(minor) = 6.482 min, t_R(major) = 8.269 min.

(3*R*)-3-Methyl-1-(1-methyl-1*H*-imidazol-2-yl)-2-(phenyl(ferrocenyl)methyl)decan-1-one (**5**)

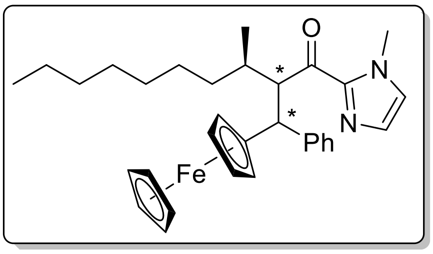

Orange oil, yield: 44% (57.0 mg), diastereoselective ratio 1:1:1:1, purified by silica gel flash chromatography (hexane/EtOAc 15:1). The diastereomers were separated by preparative thin-layer chromatography (SiO_2, hexane/EtOAc 4:1). **^{1}H NMR** (**5-diast1,2**) (400 MHz, $CDCl_3$): δ 7.48 (d, *J* = 7.2 Hz, 2H), 7.39 (t, *J* = 7.5 Hz, 4H), 7.30 – 7.27 (m, 1H), 7.18 (t, *J* = 7.6 Hz, 2H), 7.08 (d, *J* = 7.3 Hz, 1H), 7.03 (d, *J* = 1.4 Hz, 2H), 6.87 (s, 1H), 6.78 (s, 1H), 4.64 (dd, *J* = 11.7, 3.8 Hz, 1H), 4.49 (dd, *J* = 11.8, 2.9 Hz, 1H), 4.19 – 4.17 (m, 1H), 4.14 – 4.10 (m, 2H), 4.07 – 4.05 (m, 1H), 4.01 (d, *J* = 11.7 Hz, 1H), 3.96 – 3.91 (m, 3H), 3.86 (s, 3H), 3.82 – 3.77 (m, 2H), 3.67 (s, 5H), 3.66 (s, 5H), 3.61 (s, 3H), 1.66 – 1.59 (m, *J* = 9.5, 6.3 Hz, 1H), 1.49 – 1.43 (m, 1H), 1.35 – 1.06 (m, 24H), 0.97 – 0.91 (m, 6H), 0.87 – 0.79 (m, 6H) ppm. **^{1}H NMR** (**5-diast3,4**) (400 MHz, $CDCl_3$) δ 7.45 (d, *J* = 7.3 Hz, 2H), 7.38 (dd, *J* = 16.3, 8.1 Hz, 4H), 7.28 – 7.23 (m, 1H), 7.18 (t, *J* = 7.6 Hz, 2H), 7.08 (d, *J* = 7.3 Hz, 1H), 7.05 (d, *J* = 9.2 Hz, 2H), 6.88 (s, 1H), 6.78 (s, 1H), 4.60 (dd, *J* = 11.6, 4.0 Hz, 1H), 4.47 (dd, *J* = 11.8, 3.4 Hz, 1H), 4.16 – 4.10 (m, 3H), 4.09 – 4.03 (m, 2H), 4.01 – 3.96 (m, 2H), 3.96 – 3.92 (m, 1H), 3.87 (s, 3H), 3.81 (d, *J* = 6.2 Hz, 2H), 3.68 (s, 5H), 3.65 (s, 5H), 3.61 (s, 3H), 1.79 – 1.66 (m, 2H), 1.61 – 1.53 (m, 1H), 1.50 – 1.41 (m, 1H), 1.34 – 0.96 (m, 22H), 0.88 (m, 6H), 0.81 (d, *J* = 7.0 Hz, 3H), 0.77 (d, *J* = 7.0 Hz, 3H) ppm. **^{13}C NMR** (**5-diast1,2**) (101 MHz, $CDCl_3$): δ 196.1, 195.6, 145.1, 145.0, 144.1 (2 C), 143.5 (2 C), 129.1, 128.8, 128.6, 128.5, 128.1 (2 C), 127.5 (2 C), 126.5, 126.4, 126.2, 126.0, 91.9 (2 C), 70.0, 69.5, 68.5 (5 C), 68.4 (5 C), 68.2, 67.5, 67.1, 66.6, 66.0, 65.8, 55.5 (2 C), 46.7, 46.3, 36.2, 35.9, 35.8, 35.1, 34.2, 31.9, 31.8, 29.7, 29.5, 29.5, 29.2, 29.1, 27.6, 27.5, 22.6, 22.6, 14.9, 14.4, 14.1, 14.1 ppm. **^{13}C NMR** (**5-diast3,4**) (101 MHz, $CDCl_3$): δ 196.2, 195.7, 145.1, 145.0, 144.0 (2 C), 143.6 (2 C), 129.1, 128.8, 128.7, 128.5, 128.1 (2 C), 127.5 (2 C), 126.6, 126.4, 126.3, 126.1, 91.8 (2 C), 70.2, 69.6, 68.5 (10 C), 68.3, 67.6, 67.2, 66.6, 66.0, 65.9, 58.1, 57.3 (2 C), 46.4, 45.9, 36.2, 35.9, 34.9, 34.3, 31.9, 31.9, 31.2, 30.8, 30.0, 29.6, 29.4, 29.2, 28.0, 27.8, 22.7, 22.7, 19.0, 18.7, 14.2 ppm. **IR** (**5-diast1,2**) (ATR): 2954, 2922, 2852, 1664, 1408, 1380, 1106, 1026, 1000, 913, 808, 701, 482 cm^{-1}. **IR** (**5-diast3,4**) (ATR): 2953, 2921, 2851, 1664, 1408, 1287, 1154, 1106, 999, 984, 915, 805, 703, 480 cm^{-1}. **HRMS** (**5-diast1,2**) (HESI): *m/z* $[M+Na]^+$ calcd for $C_{32}H_{40}FeN_2ONa$: 547.2382, found: 574.2382. **HRMS** (**5-diast3,4**) (HESI): *m/z* $[M+Na]^+$ calcd for $C_{32}H_{40}FeN_2ONa$: 547.2382, found: 574.2382. **HPLC** (**5-diast1**): Chiral ART Amylose SA, hexane/*i*-PrOH = 90:10, 1.0 mL/min, 210 nm, t_R(major) = 4.319 min, t_R(minor) = 4.754 min. **HPLC** (**4-diast2**): Chiral ART Amylose SA, hexane/i-PrOH = 90:10, 1.0 mL/min, 210 nm, t_R(major) = 4.572 min, t_R(minor) = 4.994 min.

(3*R*)-3-Methyl-1-(1-methyl-1*H*-imidazol-2-yl)-2-(2-phenyl-4*H*-chromen-4-yl)decan-1-one (**6**)

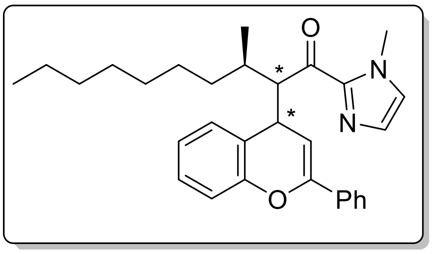

Yellow oil, yield: 22% (25.0 mg), d. r. 1:1:1. The reaction mixtrure was purified by silica gel flash chromatography (hexane/EtOAc 15:1). The diastereomers were separated by preparative thin-layer chromatography (SiO_2, hexane/EtOAc 5:1). **^{1}H NMR** (**6-diast1,2**) (400 MHz, $CDCl_3$): δ 7.65 – 7.57 (m, 4H), 7.39 – 7.30 (m, 7H), 7.21 – 7.13 (m, *J* = 9.2, 1.4 Hz, 3H), 7.10 (d, *J* = 8.9 Hz, 2H), 7.02 (dt, *J* = 7.3, 6.3 Hz, 4H), 6.92 (d, *J* = 3.4 Hz, 2H), 5.68 (d, *J* = 5.1 Hz, 1H), 5.64 (d, *J* = 5.0 Hz, 1H), 4.40 – 4.26 (m, 2H), 4.10 (dd, *J* = 10.0, 5.1 Hz, 2H), 3.94 (s, 3H), 3.92 (s, 3H), 2.25 – 2.14 (m, 2H), 1.56 – 1.44 (m, 2H), 1.40 – 1.31 (m, *J* = 10.2 Hz, 2H), 0.94 (d, *J* = 2.6 Hz, 20H), 0.92 (d, *J* = 2.6 Hz, 6H), 0.86 – 0.81 (m, 6H) ppm. **^{1}H NMR** (**6-diast3**) (400 MHz, $CDCl_3$): δ 7.71 – 7.66 (m, 2H), 7.41 – 7.31 (m, 3H), 7.26 – 7.23 (m, 1H), 6.98 – 6.93 (m, 1H), 6.90 – 6.78 (m, 3H), 6.70 (s, 1H), 5.57 (d, *J* = 5.0 Hz, 1H), 4.24 (dd, *J* = 10.3, 5.1 Hz, 1H), 4.17 (dd, *J* = 17.7, 12.6 Hz, 1H), 3.66 (s, 3H), 2.28 – 2.13 (m, 1H), 1.61 – 1.53 (m, 1H), 1.43 (dd, *J* = 8.6, 5.5 Hz, 1H), 1.28 – 1.13 (m, 10H), 0.84 (t, *J* = 6.9 Hz, 3H) ppm. **^{13}C NMR** (**6-diast1,2**) (101 MHz, $CDCl_3$): δ 195.9, 195.7, 152.3, 152.3, 149.7, 149.5, 144.4, 144.3, 134.3, 134.2, 129.0, 128.8, 128.7, 128.4, 128.2 (2 C), 128.2 (2 C), 127.6, 127.5, 127.0, 127.0, 124.8 (2 C), 124.7 (2 C), 123.2, 123.2, 116.4, 116.4, 98.8, 98.6, 58.7, 57.5, 36.2, 36.2, 35.5, 35.3, 34.8, 34.3, 32.7, 32.5, 31.8, 31.8, 30.9, 29.8, 29.7, 29.3, 29.2, 27.0, 26.4, 22.6 (2 C), 17.5, 17.2, 14.1 (2 C) ppm. **^{13}C NMR** (**6-diast3**) (101 MHz, $CDCl_3$): δ 195.1, 151.8, 150.2, 145.1, 134.6, 129.5, 128.5, 128.4, 128.3 (2 C), 127.3, 125.9, 125.0 (2 C), 123.0, 121.7, 115.6, 97.3, 59.5, 35.8, 35.6, 35.2, 31.8, 31.8, 29.8, 29.3, 27.1, 22.6, 17.0, 14.1 ppm. **IR** (**6-diast1,2**) (ATR): 2555, 2923, 2853, 1665, 1490, 1457, 1403, 1230, 1005, 912, 757, 732, 695 cm^{-1}. **IR** (**6-diast3**) (ATR): 2954, 2923, 2853, 1665, 1487, 1457, 1407, 1232, 1003, 752, 692 cm^{-1}. **HRMS** (**6-diast1,2**) (HESI): *m/z* $[M+Na]^+$ calcd for $C_{30}H_{36}N_2O_2Na$: 479.2669, found: 479.2669. **HRMS** (**6-diast3**) (HESI): *m/z* $[M+Na]^+$ calcd for $C_{30}H_{36}N_2O_2Na$: 479.2669, found: 479.2669.

(3*R*)-2-(Cyclohepta-2,4,6-trien-1-yl)-3-methyl-1-(1-methyl-1*H*-imidazol-2-yl)pentan-1-one (**3b**)

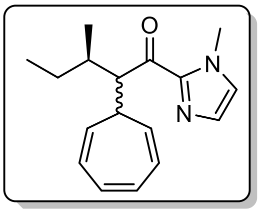

Orange oil, yield: 68% (46.0 mg), d.r. 2:1, purified by silica gel flash chromatography (hexane/EtOAc 10:1 – 1:1). The diastereomers were not separated. **^{1}H NMR** (400 MHz, $CDCl_3$): δ 7.18 – 7.17 (m, 2H), 7.04 (bs, 2H), 6.67 – 6.64 (m, 4H), 6.22 – 6.08 (m, 4H), 5.38 (dd, *J* = 9.4, 5.9 Hz, 1H), 5.31 – 5.23 (m, 3H), 4.47 (dd, *J* = 10.0, 5.7 Hz, 1H), 4.39 (dd, *J* = 8.5, 7.1 Hz, 1H), 4.00 (s, 3H), 4.00 (s, 3H),

2.19 – 2.01 (m, 4H), 1.59 – 1.43 (m, 2H), 1.22 – 0.97 (m, 2H), 0.92 – 0.86 (m, 12H) ppm. **^{13}C NMR** (101 MHz, $CDCl_3$): δ 196.8, 196.5, 145.0, 144.8, 131.0, 130.9, 130.6, 130.6, 129.2, 129.1, 127.3 (2 C), 124.8, 124.8, 124.7, 124.6, 123.8, 123.5, 123.1, 123.0, 52.0, 51.1, 39.9, 39.7, 36.5, 36.4, 36.3, 36.2, 27.9, 26.7, 16.9, 15.2, 12.0, 11.9 ppm. **HRMS** (HESI): *m/z* $[M+Na]^+$ calcd for $C_{17}H_{22}N_2ONa$: 293.1624, found: 293.1624. **HPLC**: Chiralpak IC, hexane/*i*-PrOH = 90:10, 1.0 mL/min, 254.4 nm, t_R(minor) = 5.45 min, t_R(major) = 5.69 min.

(3*R*)-2-(Cyclohepta-2,4,6-trien-1-yl)-3-cyclohexyl-1-(1-methyl-1*H*-imidazol-2-yl)butan-1-one (**3c**)

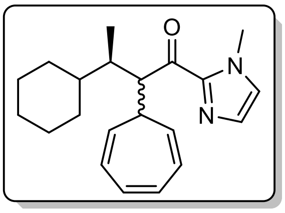

Orange oil, yield: 58% (47.0 mg), d. r. 1:1, purified by silica gel flash chromatography (hexane/EtOAc 10:1 – 1:1). The diastereomers were not separated. **^{1}H NMR** (400 MHz, $CDCl_3$): δ 7.18 – 7.17 (m, 2H), 7.04 (bs, 2H), 6.68 – 6.61 (m, 4H), 6.20 – 6.08 (m, 4H), 5.57 (dd, *J* = 9.4, 5.9 Hz, 1H), 5.36 (dd, *J* = 9.3, 5.9 Hz, 1H), 5.25 – 5.20 (m, 2H), 4.56 (dd, *J* = 9.1, 6.5 Hz, 1H), 4.49 (dd, *J* = 9.1, 6.7 Hz, 1H), 4.01 (s, 3H), 3.99 (s, 3H), 2.21 – 2.11 (m, 1H), 2.09 – 2.00 (m, 2H), 1.99 – 1.93 (m, 1H), 1.87 – 1.75 (m, 2H), 1.73 – 1.48 (m, 10H), 1.17 – 0.88 (m, 10H).0.83 (d, *J* = 7.1 Hz, 3H), 0.79 (d, *J* = 7.0 Hz, 3H) ppm. **^{13}C NMR** (101 MHz, $CDCl_3$): δ 197.5, 197.0, 145.1, 144.7, 131.0, 130.8, 130.5, 130.4, 129.2, 129.1, 127.2 (2 C), 124.8, 124.8, 124.6, 124.5, 123.6, 123.4, 123.1, 123.1, 49.2, 49.1, 41.0 (2 C), 40.2, 40.1, 40.0, 39.9, 36.4, 36.4, 32.2, 32.1, 28.7, 27.4, 26.9, 26.8, 26.7 (2 C), 26.6, 26.5, 12.6, 12.4. **HRMS** (HESI): *m/z* $[M+Na]^+$ calcd for $C_{21}H_{28}N_2ONa$: 347.2094, found: 347.2094. **HPLC**: Chiralpak IC, hexane/*i*-PrOH = 90:10, 1.0 mL/min, 254.4 nm, t_R(major) = 4.82 min, t_R(minor) = 5.46 min.

(3*S*)-2-(Cyclohepta-2,4,6-trien-1-yl)-1-(1-methyl-1*H*-imidazol-2-yl)-3-phenylbutan-1-one (**3d**)

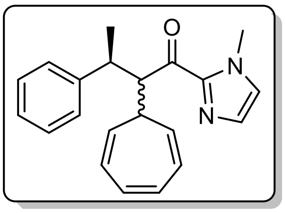

Colorless oil, yield: 21% (17.0 mg), d. r. 3:1, purified by silica gel flash chromatography (hexane/EtOAc 15:1 – 4:1). The diastereomers were not separated.**^{1}H NMR** (400 MHz, $CDCl_3$): δ 7.26 – 7.04 (m, 12H), 7.02 (bs, 1H), 6.87 (bs, 1H) 6.69 – 6.66 (m, 2H), 6.51 – 6.49 (m, 2H), 6.22 – 6.13 (m, 2H), 6.09 (dd, *J* = 9.3, 4.8 Hz, 1H), 5.96 (dd, *J* = 9.2, 4.8 Hz, 1H), 5.59 (dd, *J* = 9.3, 5.9 Hz, 1H), 5.49 (dd, *J* = 9.4, 5.8 Hz, 1H), 5.29 (dd, *J* = 9.0, 6.2 Hz, 1H), 5.08 (dd, *J* = 9.3, 6.0 Hz, 1H), 4.74 – 4.67 (m, 2H), 4.00 (s, 3H), 3.71 (s, 3H), 3.50 – 3.41 (m, 2H), 2.12 – 2.08 (m, 1H), 1.76 – 1.71 (m, 1H), 1.31 (d, *J* = 7.1 Hz, 3H), 1.24 (d, *J* = 7.0 Hz, 3H).ppm. **^{13}C NMR** (101 MHz, $CDCl_3$):

δ 196.2, 195.7, 145.0, 144.7 (2 C), 144.6, 130.9, 130.7, 130.6, 130.4, 129.3, 129.0, 128.2 (2 C), 127.9 (2 C), 127.9 (2 C), 127.8 (2 C), 127.4, 126.8, 126.4, 126.1, 124.8, 124.7, 124.6, 124.4, 123.3, 123.0, 122.7, 122.5, 53.0, 53.0, 41.3, 41.3, 40.8, 40.3, 36.4, 36.0, 19.8, 19.3 ppm. **HRMS** (HESI): *m/z* $[M+Na]^+$ calcd for $C_{21}H_{22}N_2ONa$: 341.1624, found: 341.1624.

(3*S*)-2-(Cyclohepta-2,4,6-trien-1-yl)-3-(4-fluorophenyl)-1-(1-methyl-1*H*-imidazol-2-yl)butan-1-one (**3e**)

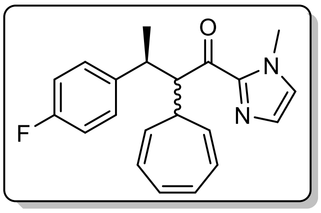

Light brown solid, yield: 69% (58.0 mg), diastereoselective ratio 3:1, purified by silica gel flash chromatography (hexane/EtOAc 15:1-4:1). The diastereomers were separated by flash chromatography. **^{1}H NMR (3e-diast1)** (400 MHz, $CDCl_3$): δ 7.20 – 7.14 (m, 2H), 7.12 (d, *J* = 0.7 Hz, 1H), 6.91 (bs, 1H), 6.86 – 6.79 (m, 2H), 6.69 – 6.65 (m, 2H), 6.22 – 6.12 (m, 2H), 5.56 (dd, *J* = 9.4, 5.9 Hz, 1H), 5.26 (dd, *J* = 9.3, 6.0 Hz, 1H), 4.68 (dd, *J* = 9.1, 7.3 Hz, 1H), 3.76 (s, 3H), 3.50 – 3.42 (m, 1H), 2.11 – 2.03 (m, 1H), 1.28 (d, *J* = 7.1 Hz, 3H) ppm. **^{1}H NMR (3e-diast2)** (400 MHz, $CDCl_3$): δ 7.20 – 7.16 (m, 3H), 7.03 (bs, 1H), 6.93 – 6.84 (m, 2H), 6.54 – 6.50 (m, 2H), 6.13 – 6.06 (m, 1H), 6.00 – 5.94 (m, 1H), 5.47 (dd, *J* = 9.4, 5.8 Hz, 1H), 5.07 (dd, *J* = 9.3, 6.1 Hz, 1H), 4.64 (dd, *J* = 9.4, 7.5 Hz, 1H), 4.00 (s, 3H), 3.48 – 3.40 (m, 1H), 1.69 (dd, *J* = 12.7, 6.1 Hz, 1H), 1.22 (d, *J* = 7.0 Hz, 3H) ppm. **^{13}C NMR (3e-diast1)** (101 MHz, $CDCl_3$): δ 195.5, 164.9, 160.0, 144.6, 140.4, 131.0, 130.7, 129.4, 129.3, 129.1, 124.9, 124.7, 123.2, 122.5, 114.7, 114.5, 52.9, 40.5, 40.2, 36.1, 19.9 ppm. **^{13}C NMR (3e-diast2)** (101 MHz, $CDCl_3$): δ 196.0, 162.7, 160.3, 144.9, 140.3, 140.2, 130.8, 130.5, 129.4, 129.1, 129.1, 124.8, 124.5, 122.8, 115.0, 114.8, 53.1, 40.8, 40.5, 36.4, 19.4 ppm. **HRMS (3e-diast1)** (HESI): *m/z* $[M+Na]^+$ calcd for $C_{21}H_{21}FN_2ONa$: 359.1530, found: 359.1530. **HRMS (3e-diast2)** (HESI): *m/z* $[M+Na]^+$ calcd for $C_{21}H_{21}FN_2ONa$: 359.1530, found: 359.1530.

(3*S*)-2-(Cyclohepta-2,4,6-trien-1-yl)-3-(4-methoxyphenyl)-1-(1-methyl-1*H*-imidazol-2-yl)butan-1-one (**3f**)

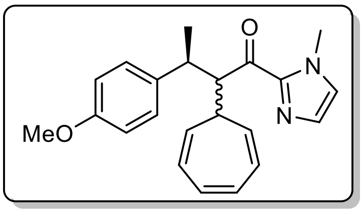

Light yellow oil, yield: 25% (22.0 mg), d. r. 3:1, purified by silica gel flash chromatography (hexane/EtOAc 15:1 – 4:1). The diastereomers were not separated. **^{1}H NMR** (400 MHz, $CDCl_3$): δ 7.20 – 7.05 (m, 6H), 7.03 (bs, 1H), 6.89 (bs, 1H), 6.74 (d, *J* = 8.7 Hz, 2H), 6.69 (d, *J* = 8.7 Hz, 2H), 6.68 – 6.65 (m, 2H), 6.52 – 6.50 (m, 2H), 6.19 – 6.14 (m, 2H), 6.09 (dd, *J* = 9.3, 4.9 Hz, 1H), 5.96 (dd, *J* = 9.1, 4.7 Hz, 1H), 5.56 (dd, *J* = 9.4, 5.9 Hz, 1H), 5.49 (dd, *J* = 9.4, 5.8 Hz,

1H), 5.28 (dd, J = 9.2, 6.0 Hz, 1H), 5.08 (dd, J = 9.3, 6.0 Hz, 1H), 4.71 – 4.60 (m, 2H), 4.01 (s, 3H), 3.80 (s, 3H), 3.75 (s, 3H), 3.71 (s, 3H), 3.45 – 3.36 (m, 2H), 2.13 – 2.03 (m, 1H), 1.75 – 1.70 (m, 1H), 1.26 (d, J = 3.5 Hz, 3H), 1.21 (d, J = 7.0 Hz, 3H) ppm. **^{13}C NMR** (101 MHz, $CDCl_3$): δ 196.4, 195.8, 158.1, 157.8, 145.0, 144.7, 136.8, 136.7, 130.9, 130.7, 130.6, 130.4, 129.3, 129.0, 128.9, 128.6 (2 C), 128.5, 127.4, 126.9, 124.8, 124.7, 124.6, 124.3, 123.4, 123.1, 122.7, 122.6, 113.5 (2 C), 113.3 (2 C), 55.2, 55.2, 53.3, 53.1, 40.9, 40.5, 40.4, 40.3, 36.4, 36.1, 20.0, 19.6 ppm. **HRMS** (HESI): m/z $[M+Na]^+$ calcd for $C_{22}H_{24}N_2O_2Na$: 371.1730, found: 371.1730.

(3*R*)-1-(1-Benzyl-1*H*-imidazol-2-yl)-2-(cyclohepta-2,4,6-trien-1-yl)-3-methyldecan-1-one (**3g**)

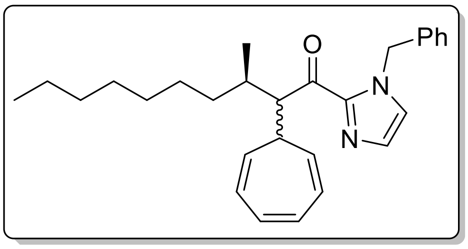

Yellow oil, yield: 19% (20.0 mg), d. r. 1:1, purified by silica gel flash chromatography (hexane/EtOAc 4:1). The diastereomers were not separated. **^{1}H NMR** (400 MHz, $CDCl_3$): δ 7.34 – 7.24 (m, 7H), 7.24 – 7.19 (m, 3H), 7.16 – 7.11 (m, 4H),.6.70 – 6.61 (m, 4H), 6.23 – 6.12 (m, 2H), 6.12 – 6.04 (m, 2H), 5.77 (s, 1H), 5.74 (s, 1H), 5.58 – 5.47 (m, 2H), 5.33 – 5.16 (m, 4H), 4.47 (dd, J = 10.4, 5.4 Hz, 1H), 4.38 (dd, J = 9.0, 6.7 Hz, 1H), 2.13 – 2.20 (m, 4H), 1.35 – 1.15 (m, 24H), 0.88 – 0.85 (m, 6H), 0.83 (d, J = 6.9 Hz, 3H), 0.72 (d, J = 6.8 Hz, 3H) ppm. **^{13}C NMR** (101 MHz, $CDCl_3$): δ 196.7, 196.5, 144.6, 144.4, 136.6 (2 C), 131.0, 130.9, 130.5 (2 C), 129.5, 129.4, 128.8 (2 C), 128.8 (2 C), 128.0 (2 C), 127.5 (2 C), 127.5 (2 C), 126.4 (2 C), 124.8 (2 C), 124.6 (2 C), 123.9, 123.5, 123.1, 122.9, 52.5, 52.0, 52.0, 51.4, 39.8, 39.6, 35.1, 34.5, 34.5, 33.5, 31.9 (2 C), 29.6, 29.6, 29.3, 29.2, 27.3, 27.3, 22.7 (2 C), 17.6, 15.4, 14.1 (2 C) ppm. **HRMS** (HESI): m/z $[M+Na]^+$ calcd for $C_{28}H_{26}N_2ONa$: 439.2720, found: 339.2720.

(3*R*)-1-(1-Butyl-1*H*-imidazol-2-yl)-2-(cyclohepta-2,4,6-trien-1-yl)-3-methyldecan-1-one (**3h**)

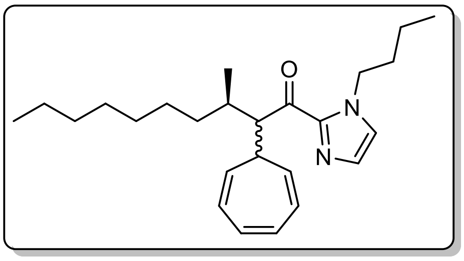

Yellow oil, yield: 21% (20.0 mg), d. r. 1:1, purified by silica gel flash chromatography (hexane/EtOAc 4:1). The diastereomers were not separated. **^{1}H NMR** (400 MHz, $CDCl_3$): δ 7.22 – 7.15 (m, 2H), 7.08 (bs, 2H), 6.67 – 6.65 (m, 4H), 6.24 – 6.05 (m, 4H), 5.36 (dd, J = 9.3, 5.9 Hz, 1H), 5.31 – 5.21 (m, 3H), 4.56 – 4.23 (m, 6H), 2.24 – 2.14 (m, 2H), 2.11 – 1.99 (m, 2H), 1.77 – 1.70 (m, 4H), 1.48 – 1.13 (m, 28H), 0.98 – 0.79 (m, 18H) ppm. **^{13}C NMR** (101

MHz, $CDCl_3$): δ 196.6, 196.4, 144.5, 144.3, 131.0, 130.9, 130.6 (2 C), 129.2, 129.1, 126.3 (2 C), 124.7 (2 C), 124.6, 124.6, 123.9, 123.5, 123.1, 123.0, 52.3, 51.4, 48.7, 48.7, 39.9, 39.8, 35.1, 34.5, 33.8, 33.2 (2 C), 33.2, 31.9 (2 C), 29.6 (2 C), 29.2 (2 C), 27.4, 27.3, 22.7 (2 C), 19.7 (2 C), 17.6, 15.8, 14.1 (2 C), 13.7 (2 C) ppm. **HRMS** (HESI): *m/z* $[M+Na]^+$ calcd for $C_{25}H_{38}N_2ONa$: 405.2876, found: 405.2876.

(3*R*)-2-(Cyclohepta-2,4,6-trien-1-yl)-3-methyl-1-(1-methyl-1*H*-benzo[*d*]imidazol-2-yl)decan-1-one (**3i**)

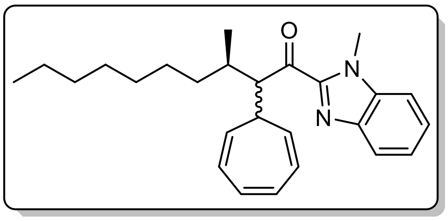

Yellow oil, yield: 65% (66.0 mg), d. r. 1:1, purified by silica gel flash chromatography (hexane/EtOAc 4:1). The diastereomers were not separated. **^{1}H NMR** (400 MHz, $CDCl_3$): δ 7.96 – 7.93 (m, 2H), 7.45 – 7.43 (m, 4H), 7.42 – 7.35 (m, 2H), 6.69 – 6.67 (m, 4H), 6.26 – 6.09 (m, 4H), 5.40 (dd, *J* = 9.3, 5.8 Hz, 1H), 5.35 – 5.29 (m, 3H), 4.75 (dd, *J* = 10.3, 5.4 Hz, 1H), 4.66 (dd, *J* = 9.0, 6.7 Hz, 1H), 4.14 (s, 3H), 4.14 (s, 3H), 2.33 – 2.20 (m, 2H), 2.16 – 2.04 (m, 2H), 1.26 – 1.19 (m, 24H), 0.96 (d, *J* = 6.9 Hz, 3H), 0.92 (d, *J* = 6.8 Hz, 3H), 0.88 – 0.82 (m, 6H) ppm. **^{13}C NMR** (101 MHz, $CDCl_3$): δ 199.9, 199.6, 148.0, 147.8, 141.7 (2 C), 137.2 (2 C), 131.0, 131.0, 130.6 (2 C), 126.0, 126.0, 124.9, 124.9, 124.8, 124.7, 123.8, 123.7, 123.6, 123.3, 122.9, 122.8, 122.2 (2 C), 110.6 (2 C), 53.4, 53.1 (2 C), 52.1, 40.0, 39.8, 35.2, 34.7, 34.6, 33.7, 32.5, 32.4, 31.8 (2 C), 29.6, 29.3, 27.4 (2 C), 22.7, 22.6, 17.6, 15.6, 14.1, 14.1 ppm. **HRMS** (HESI): *m/z* $[M+Na]^+$ calcd for $C_{26}H_{34}N_2ONa$: 313.2563, found: 413.2563.

N,*N*-Dimethyl-4-(1-(1-methyl-1*H*-indol-3-yl)ethyl)aniline (**S1**)

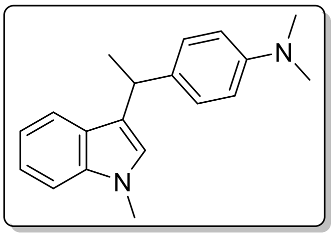

Brown solid, yield: 70% (56.0 mg), purified by flash chromatography (SiO_2, Hex/EtOAc 10:1). **Mp**: 93.2-94.4 °C. **^{1}H NMR** (400 MHz, $CDCl_3$): δ 7.43 (d, *J* = 7.9 Hz, 1H), 7.27 (d, *J* = 7.3 Hz, 1H), 7.21 – 7.16 (m, 3H), 7.00 (t, *J* = 7.5 Hz, 1H), 6.80 (s, 1H), 6.70 (d, *J* = 8.6 Hz, 2H), 4.30 (q, *J* = 7.1 Hz, 1H), 3.75 (s, 3H), 2.91 (s, 6H), 1.67 (d, *J* = 7.1 Hz, 3H) ppm. **^{13}C NMR** (101 MHz, $CDCl_3$): δ 149.0, 137.3, 135.2, 128.0 (2 C), 127.3, 125.8, 121.3, 120.8, 120.0, 118.5, 112.9 (2 C), 109.0, 40.9 (2 C), 35.8, 32.6, 22.6 ppm. **IR** (ATR): 3056, 2964, 2922, 1616, 1523, 1474, 1354, 1325, 1155, 1084, 946 cm^{-1}. **HRMS** (HESI): *m/z* $[M+Na]^+$ calcd for $C_{19}H_{22}N_2Na$: 301.1675, found: 301.1675.

5. Copies of NMR, MS, HRMS, and HPLC spectra

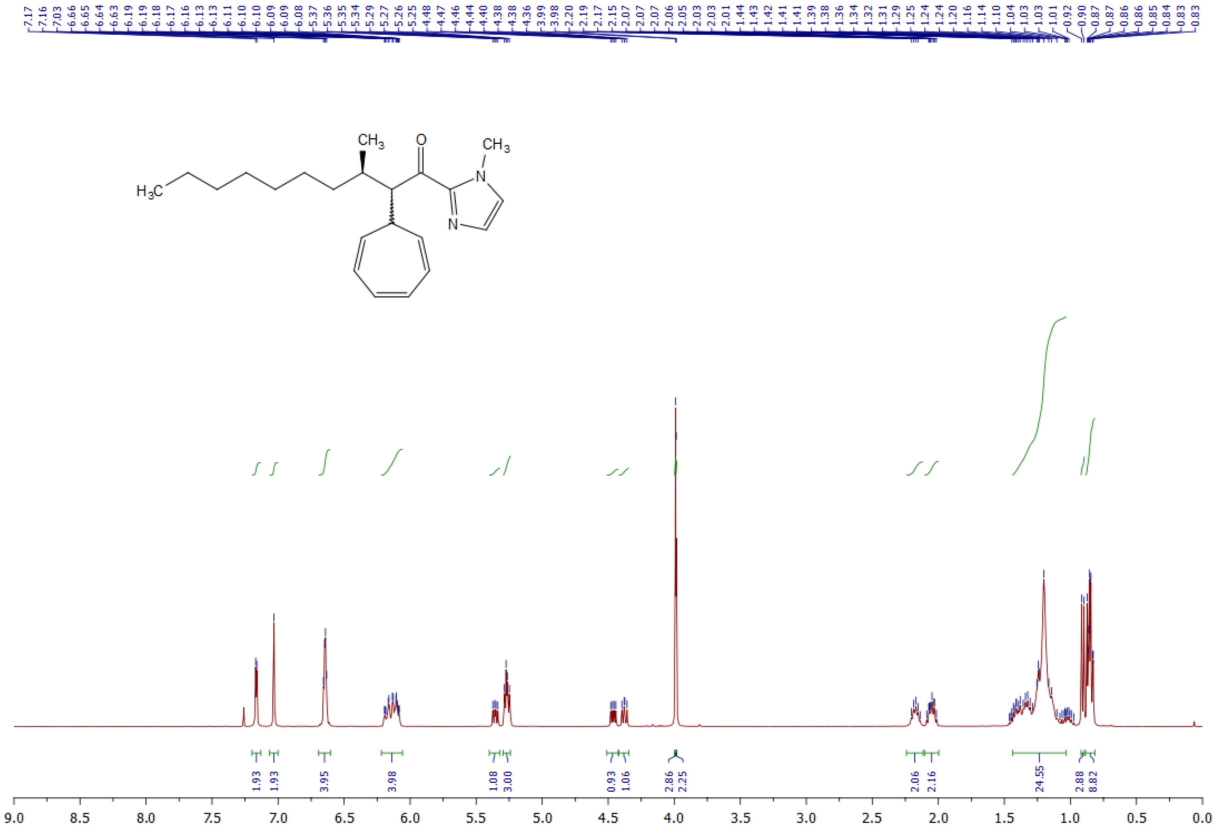

^{1}H NMR spectrum of compound **3a**.

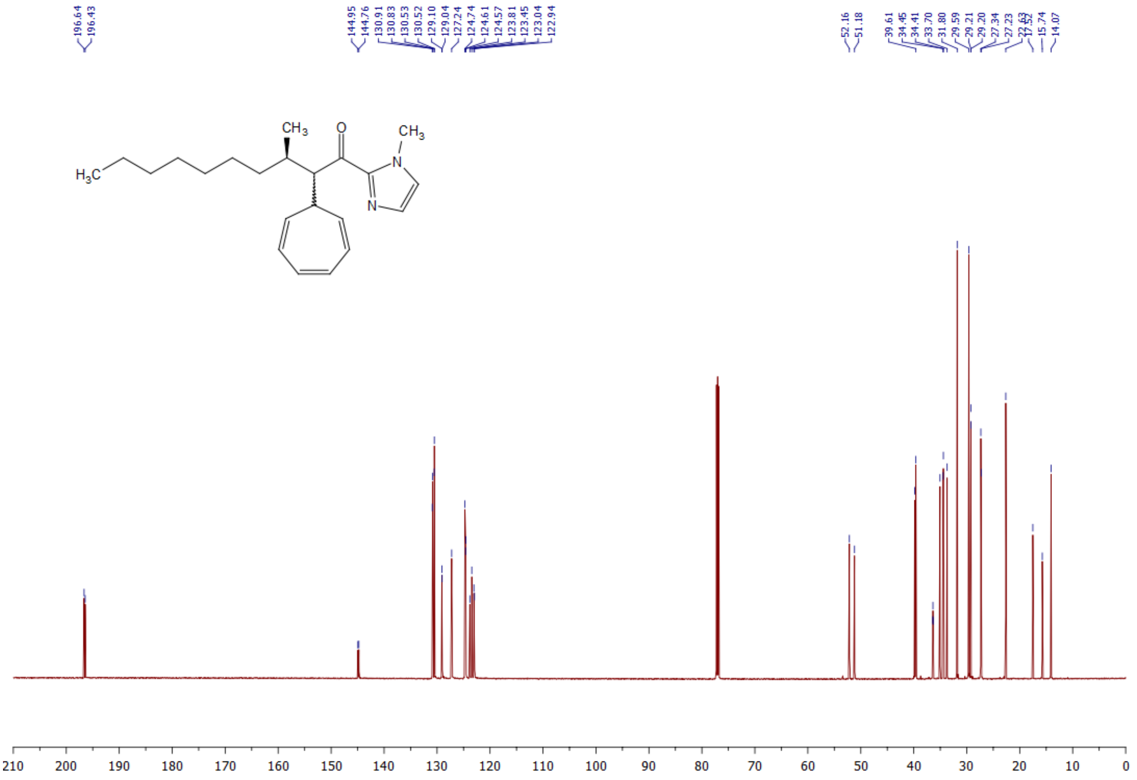

^{13}C NMR spectrum of compound **3a**.

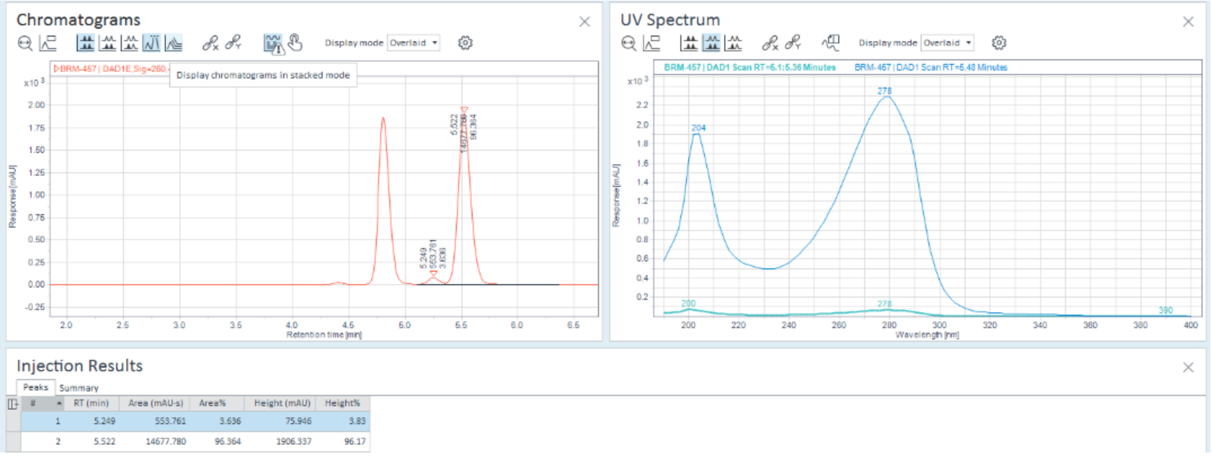

HPLC chromatogram of compound **3a** (non-racemic).

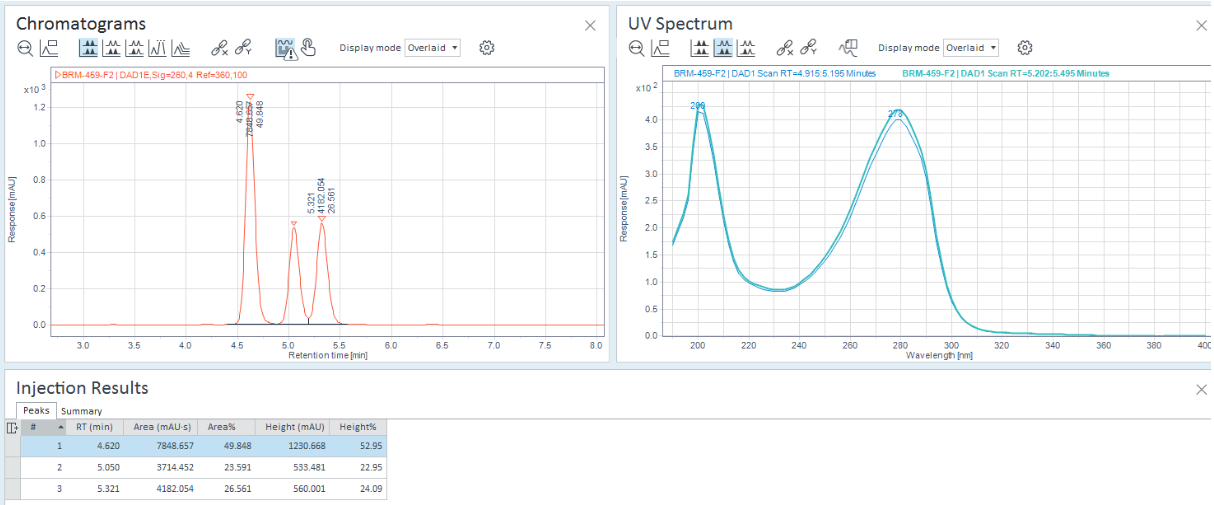

HPLC chromatogram of compound **3a** (racemic).

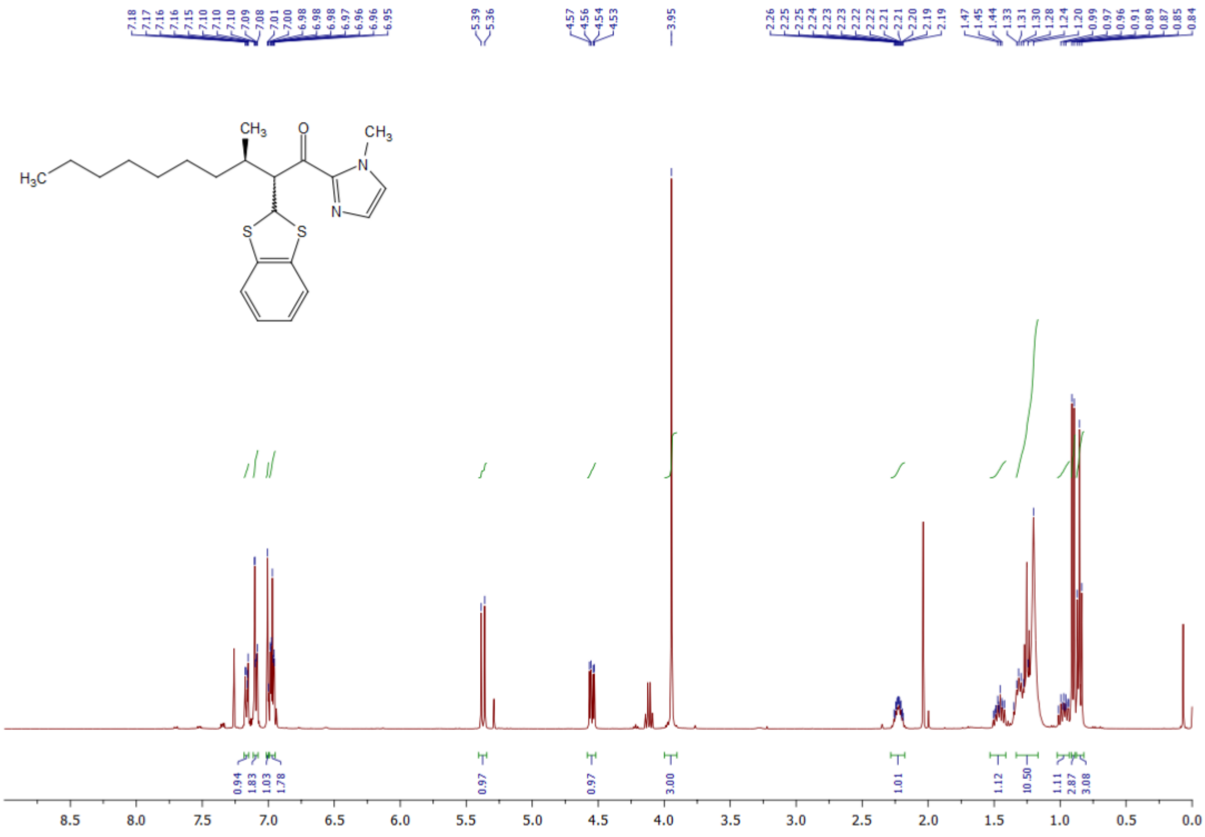

^{1}H NMR spectrum of compound **4 (diastereomer 1)**.

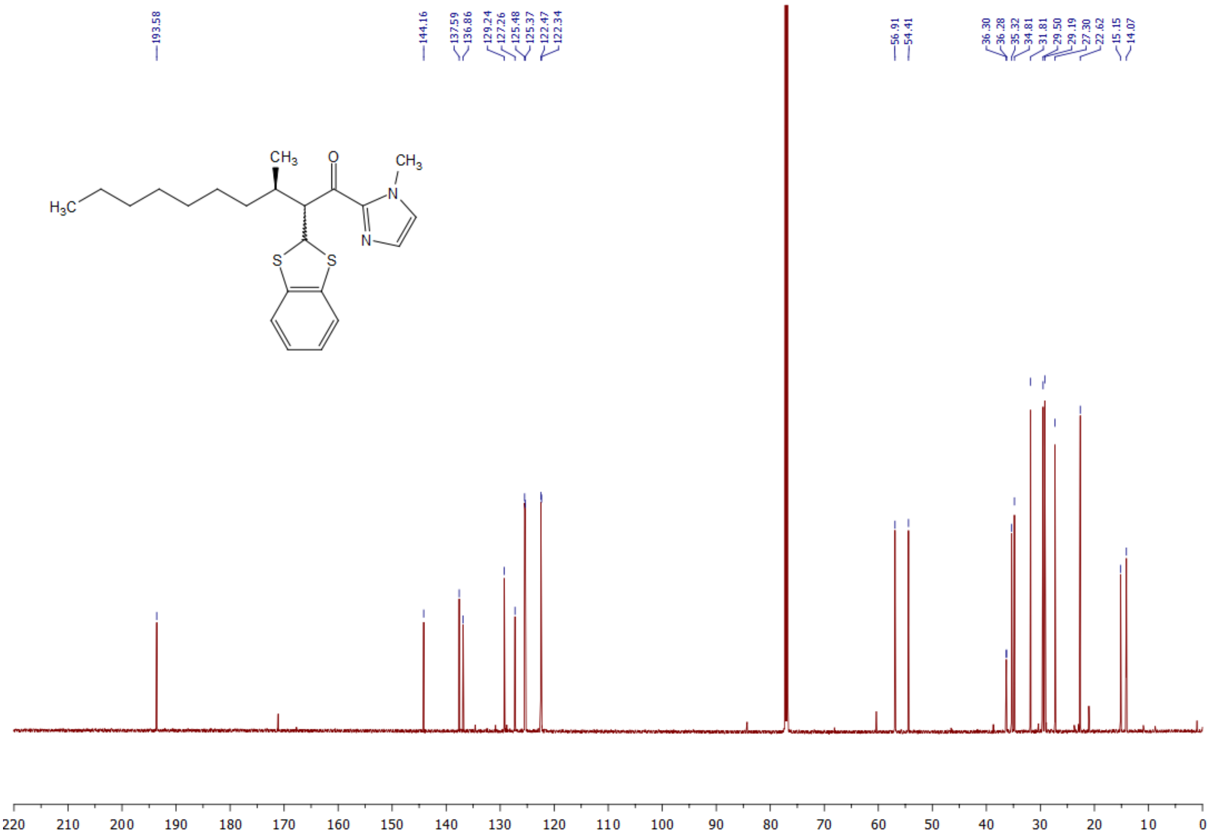

^{13}C NMR spectrum of compound **4 (diastereomer 1)**.

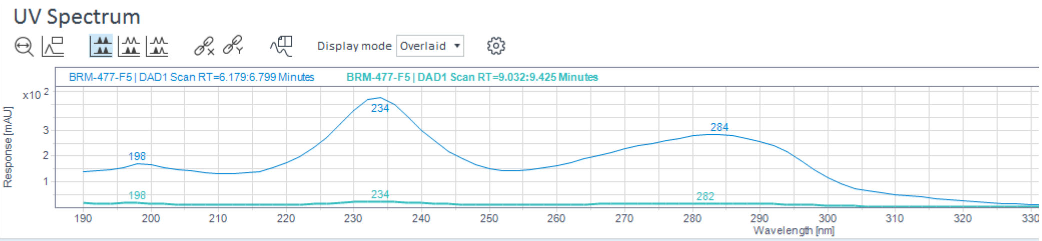

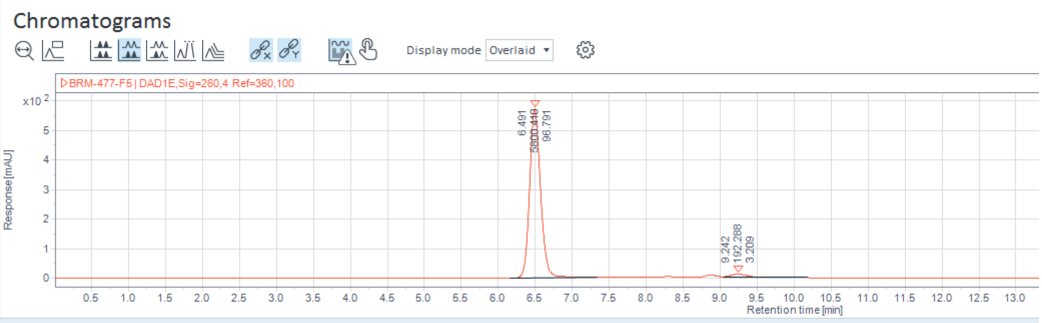

Injection Results

#	RT (min)	Area (mAU·s)	Area%	Height (mAU)	Height%
1	6.491	5800.410	96.791	571.148	98.05
2	9.242	192.288	3.209	11.356	1.95

HPLC chromatogram of compound **4 (diastereomer 1)** (non-racemic).

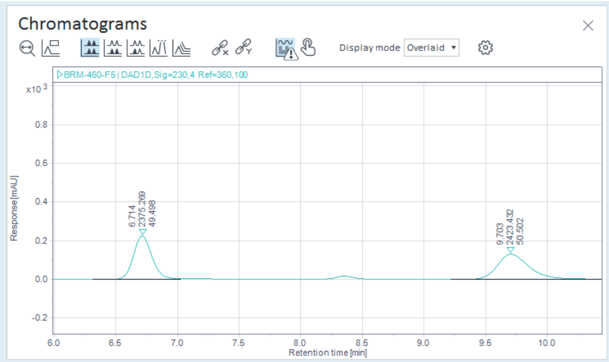

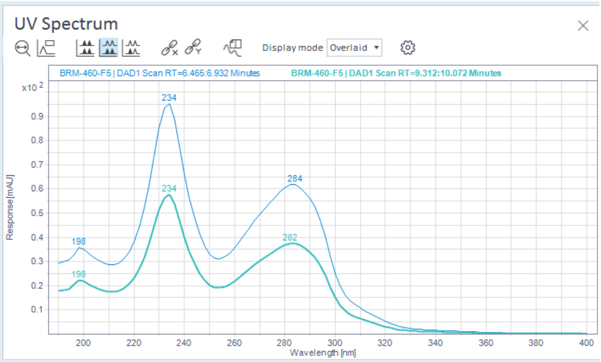

Injection Results

#	RT (min)	Area (mAU·s)	Area%	Height (mAU)	Height%
1	6.714	2375.269	49.498	225.617	63.39
2	9.703	2423.432	50.502	130.299	36.61

HPLC chromatogram of compound **4 (diastereomer 1)** (racemic).

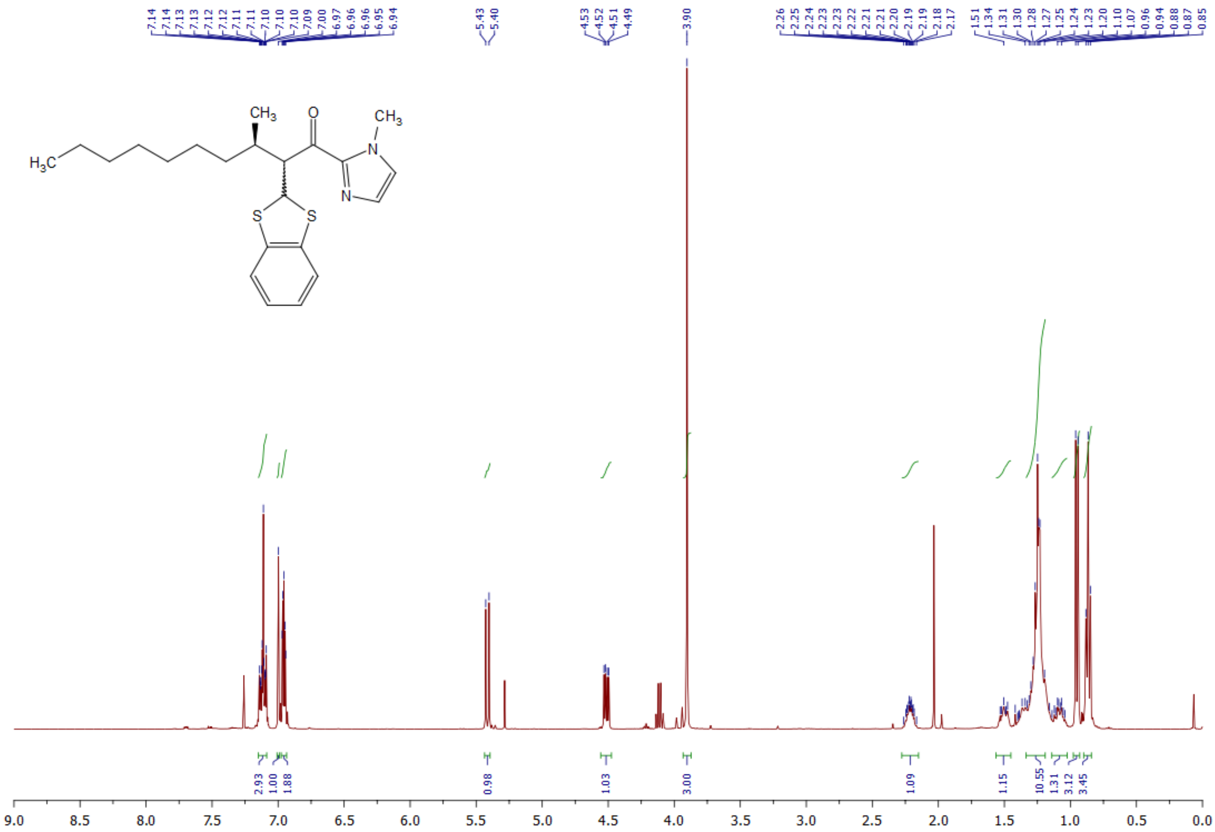

^{1}H NMR spectrum of compound **4 (diastereomer 2)**.

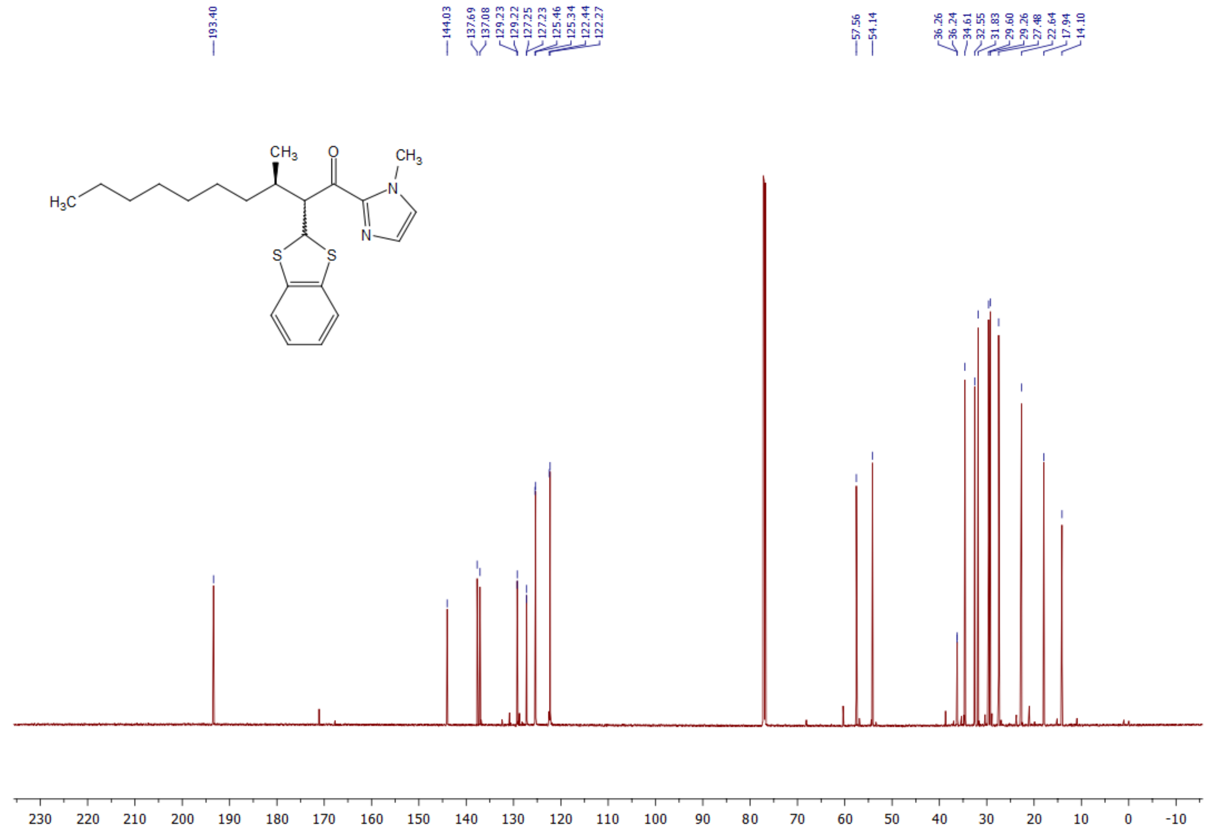

^{13}C NMR spectrum of compound **4 (diastereomer 2)**.

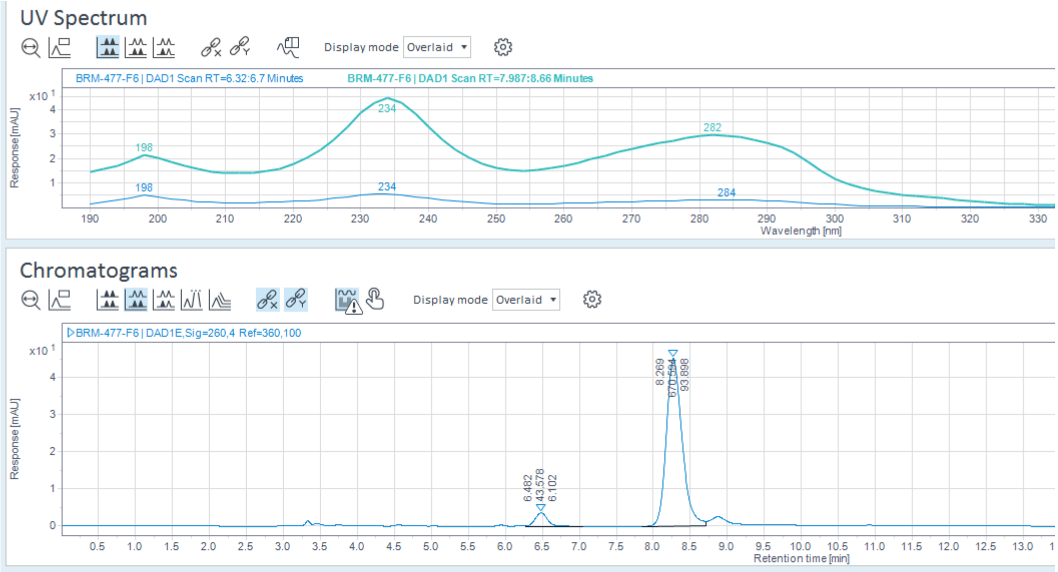

Injection Results

Peaks Summary

#	RT (min)	Area (mAU·s)	Area%	Height (mAU)	Height%
1	6.482	43.578	6.102	3.672	7.50
2	8.269	670.594	93.898	45.260	92.50

HPLC chromatogram of compound **4 (diastereomer 2)** (non-racemic).

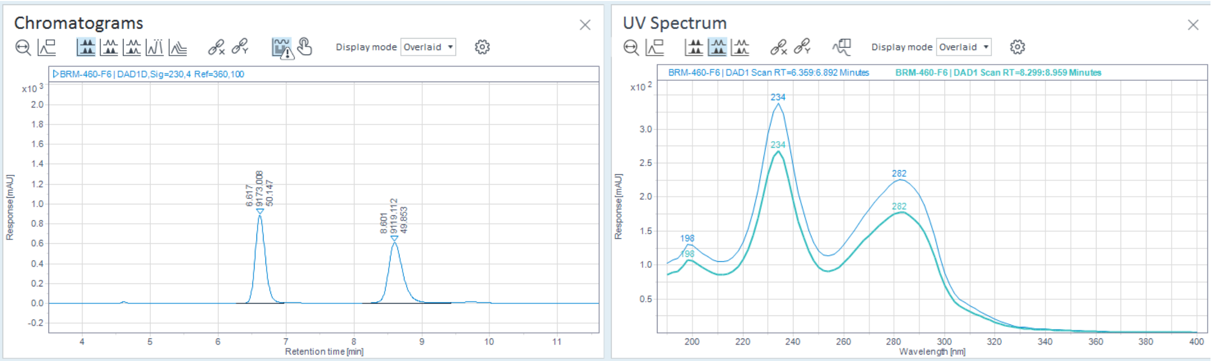

Injection Results

Peaks Summary

#	RT (min)	Area (mAU·s)	Area%	Height (mAU)	Height%
1	6.617	9173.008	50.147	884.154	59.15
2	8.601	9119.112	49.853	610.540	40.85

HPLC chromatogram of compound **4 (diastereomer 2)** (racemic).

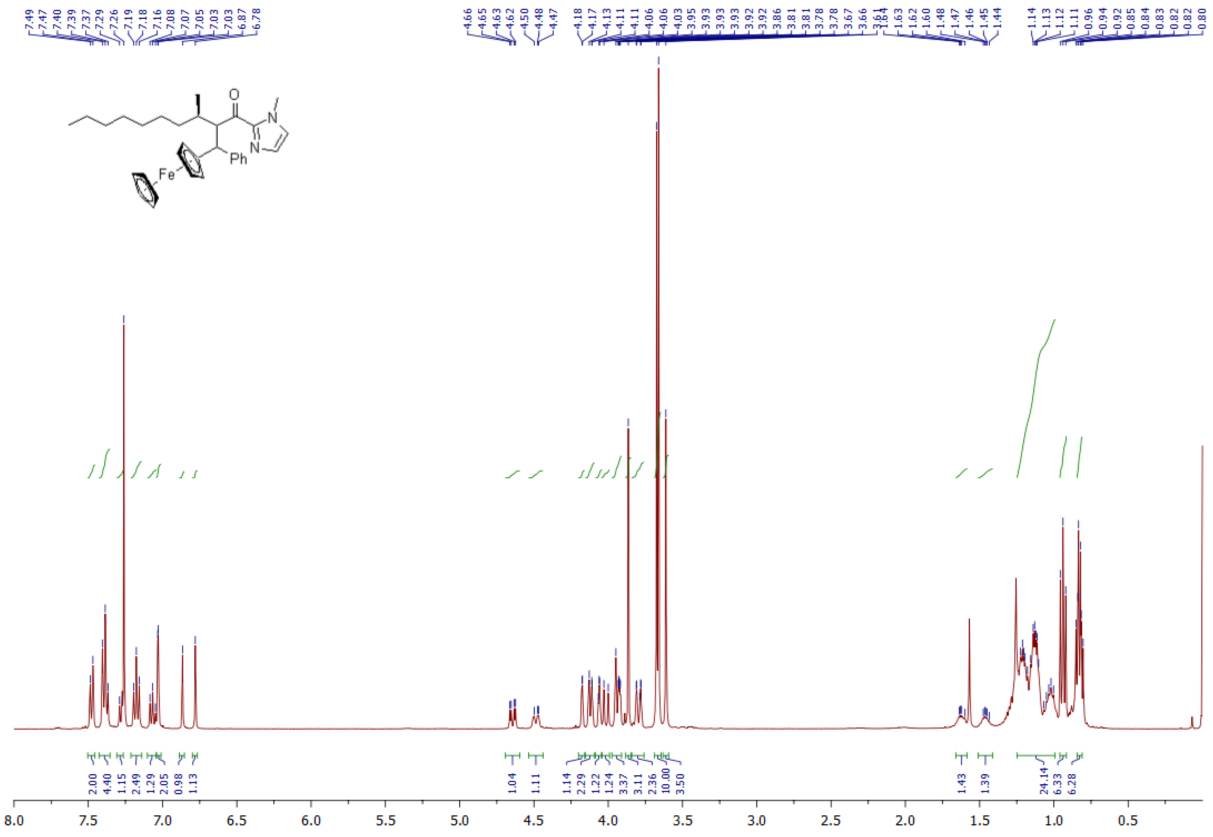

^{1}H NMR spectrum of compound **5 (diastereomer 1,2)**.

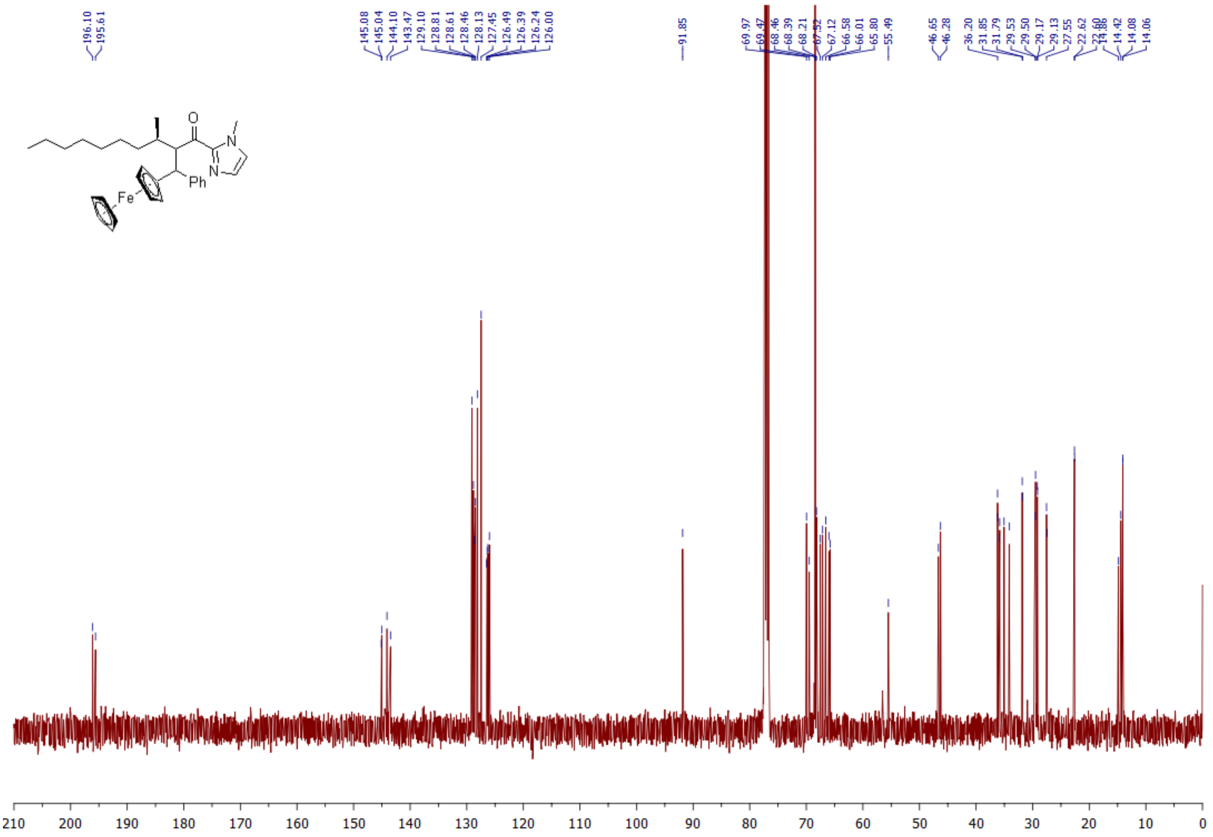

^{13}C NMR spectrum of compound **5 (diastereomer 1,2)**.

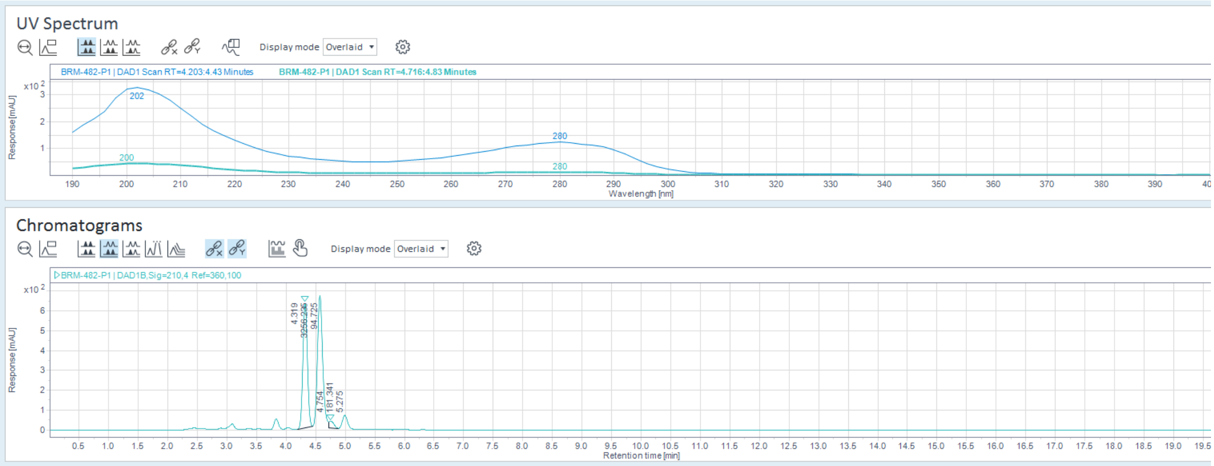

Injection Results

Peaks | Summary

#	RT (min)	Area (mAU·s)	Area%	Height (mAU)	Height%
1	4.319	3256.235	94.725	630.201	95.06
2	4.754	181.341	5.275	32.720	4.94

HPLC chromatogram of compound **5 (diastereomer 1)** (non-racemic).

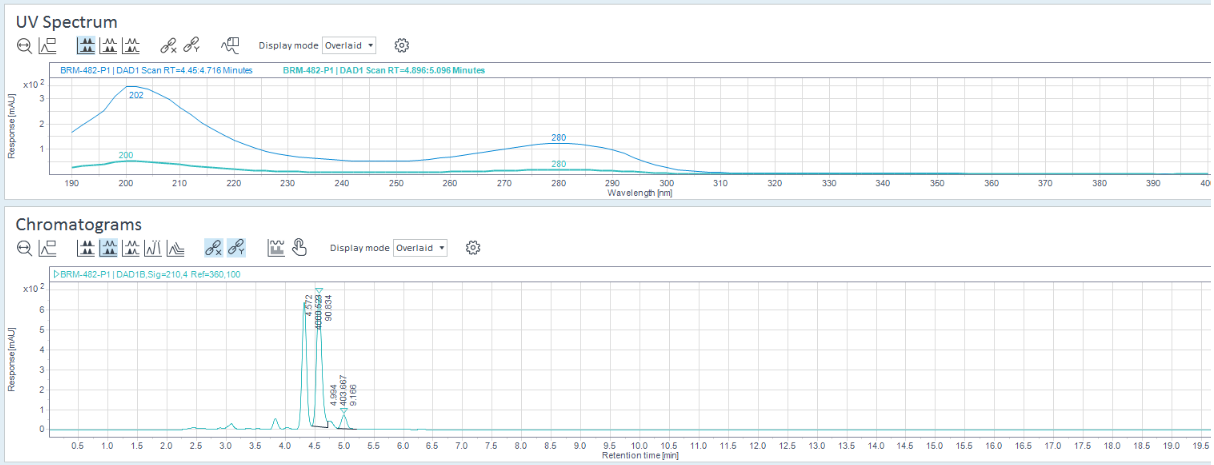

Injection Results

Peaks | Summary

#	RT (min)	Area (mAU·s)	Area%	Height (mAU)	Height%
1	4.572	4000.523	90.834	661.863	90.71
2	4.994	403.667	9.166	67.753	9.29

HPLC chromatogram of compound **5 (diastereomer 2)** (non-racemic).

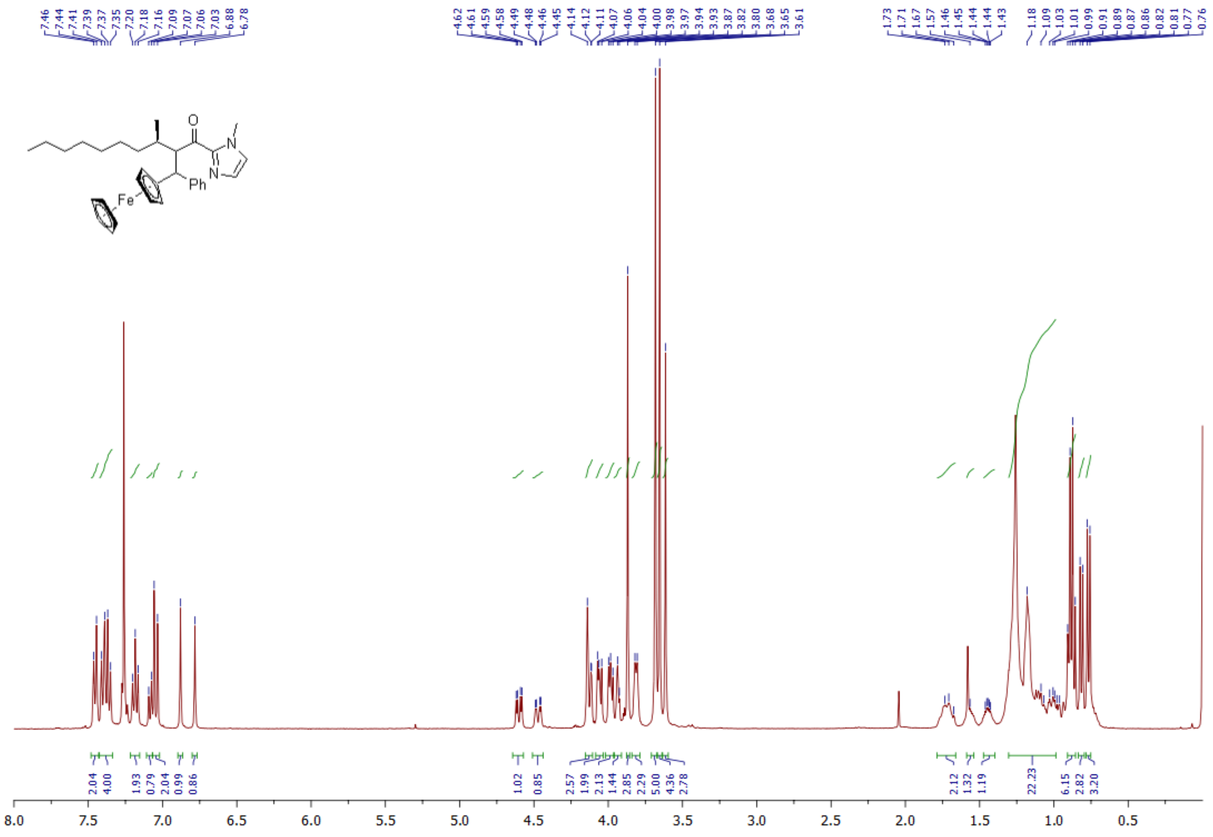

^{1}H NMR spectrum of compound **5 (diastereomer 3,4)**.

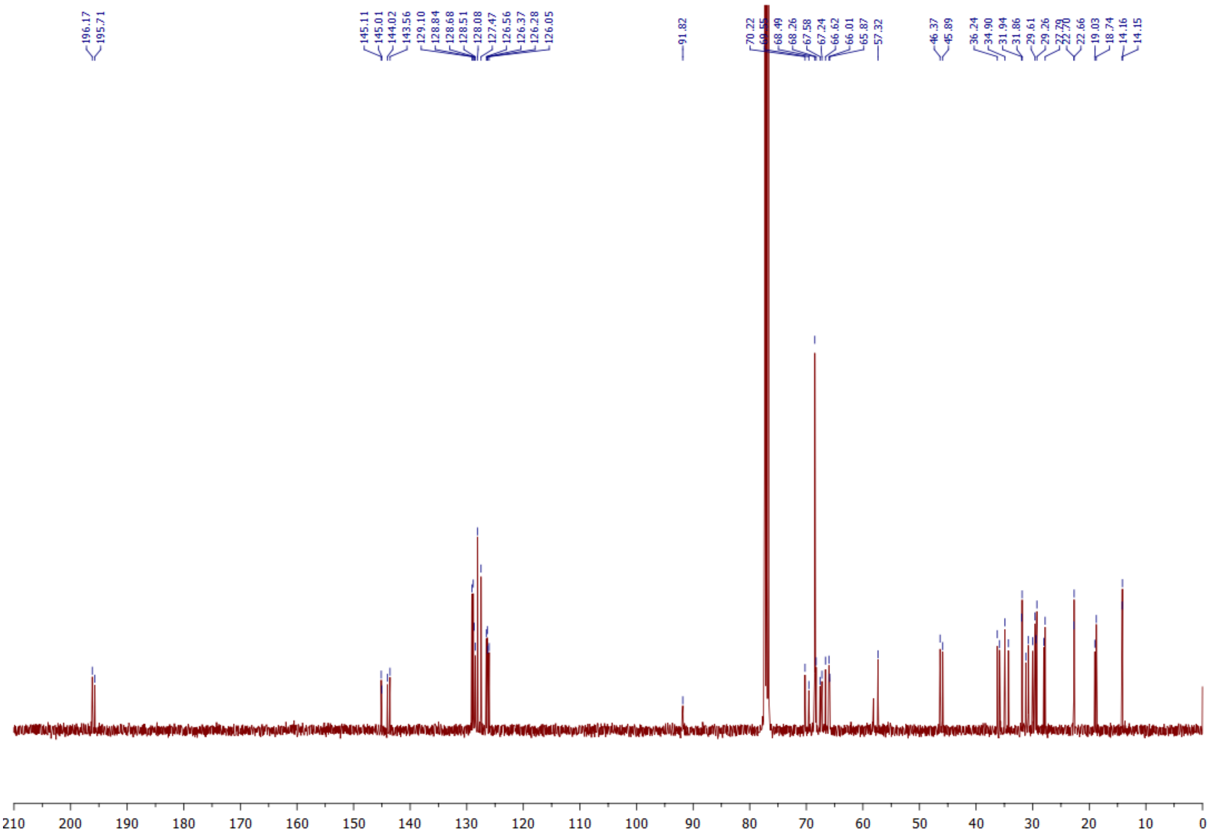

^{13}C NMR spectrum of compound **5 (diastereomer 3,4)**.

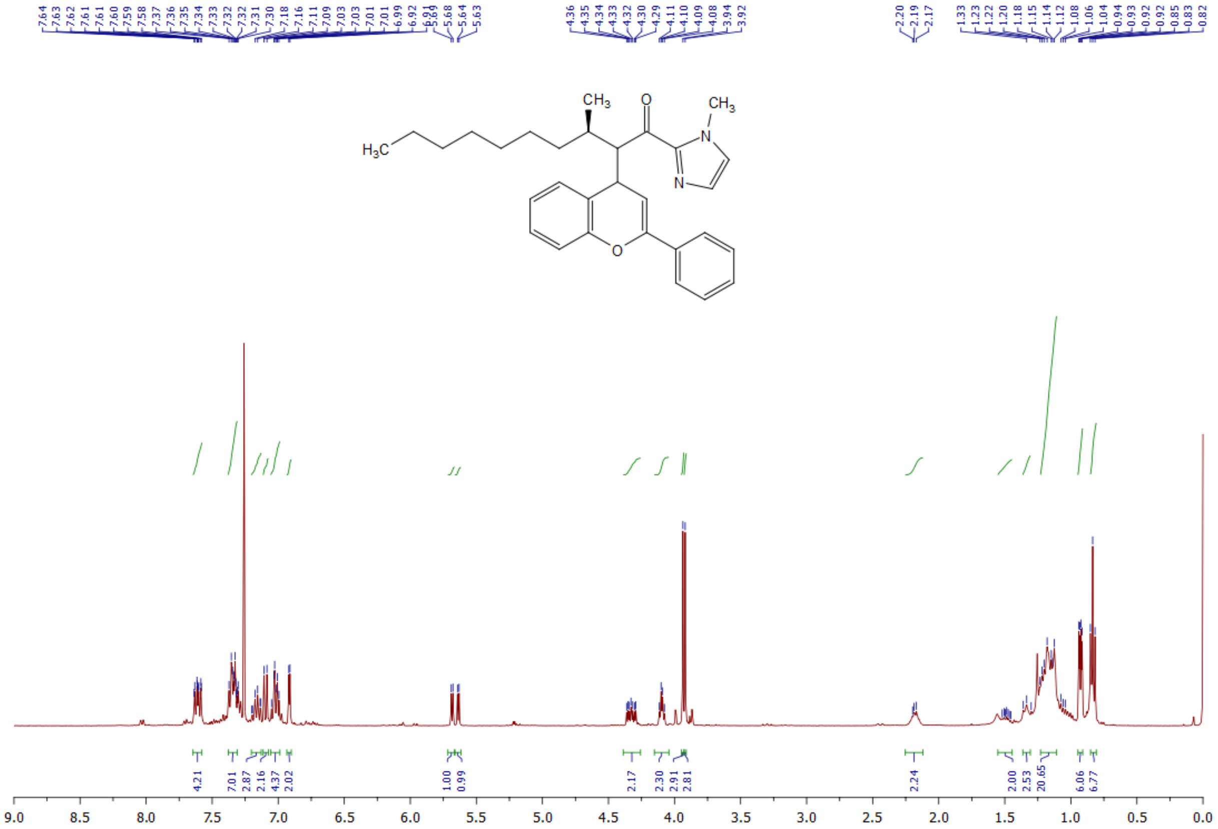

^{1}H NMR spectrum of compound **6 (diastereomer 1,2)**.

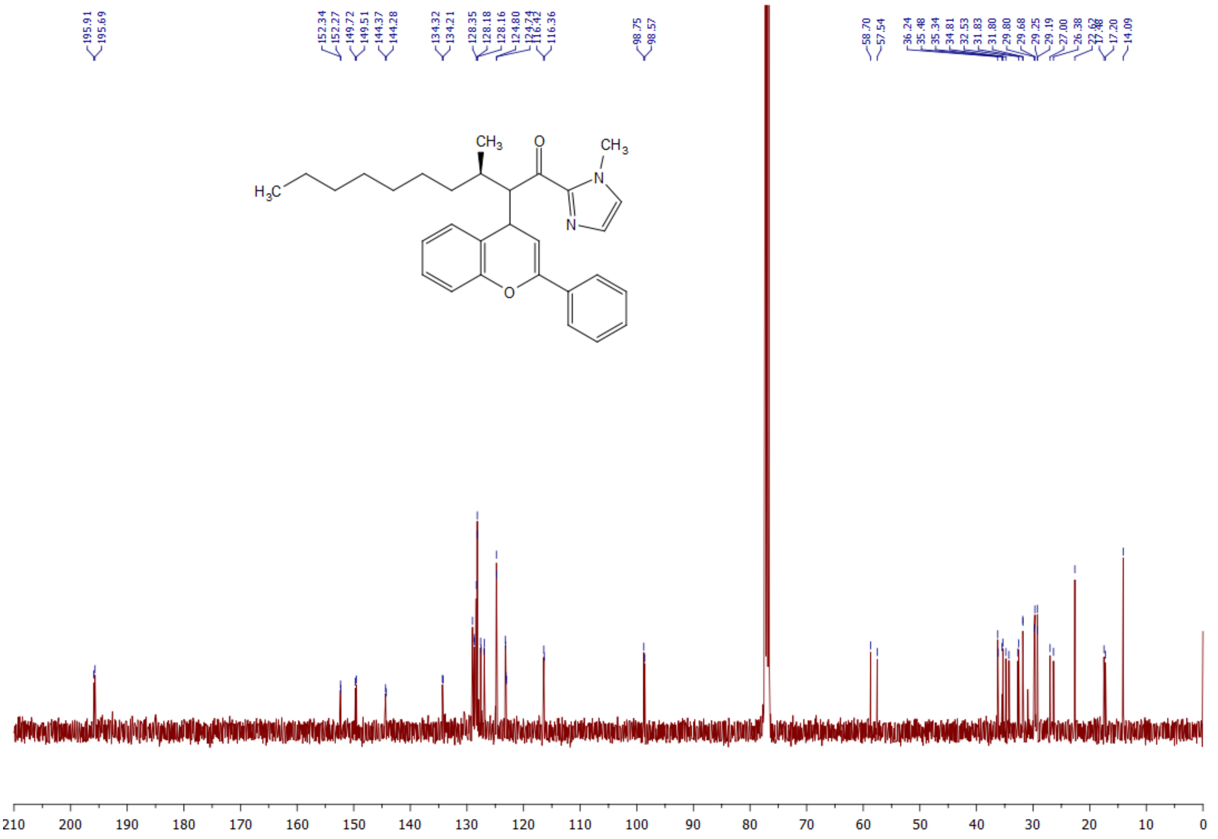

^{13}C NMR spectrum of compound **6 (diastereomer 1,2)**.

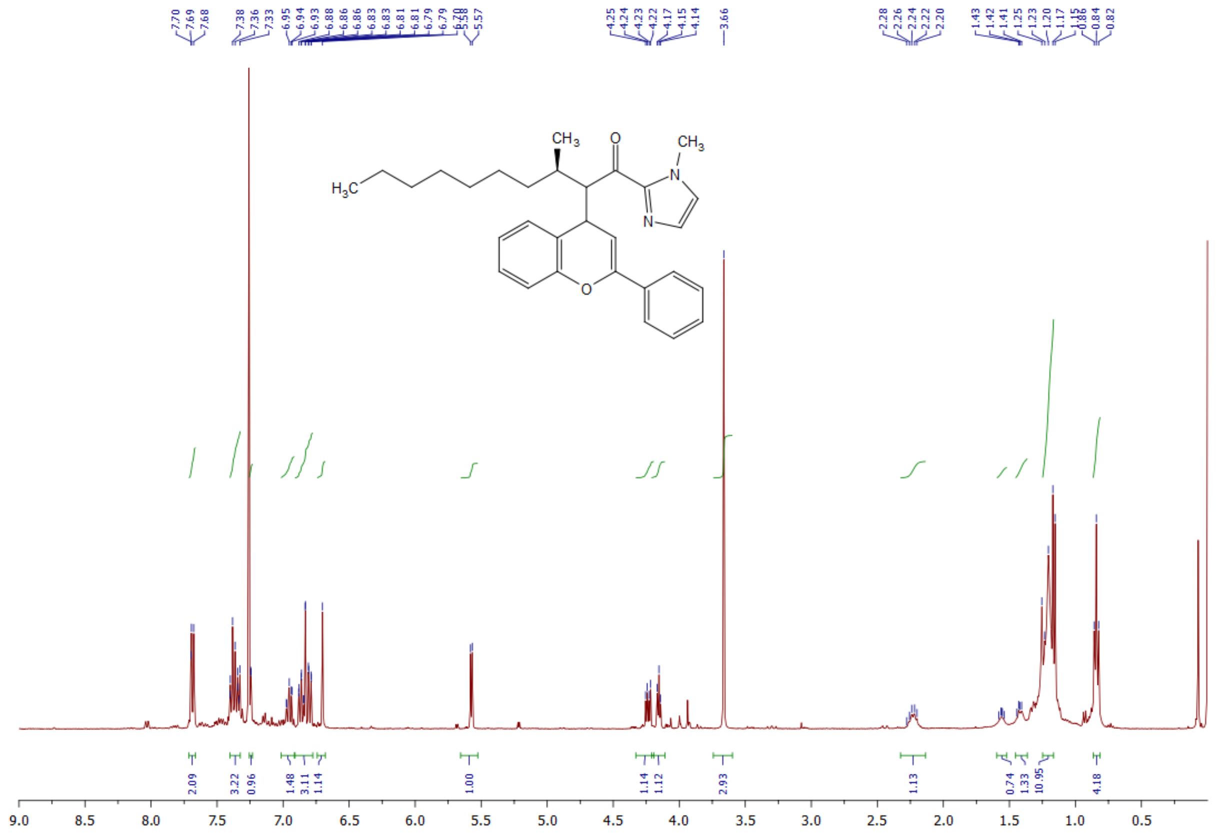

^{1}H NMR spectrum of compound **6 (diastereomer 3)**.

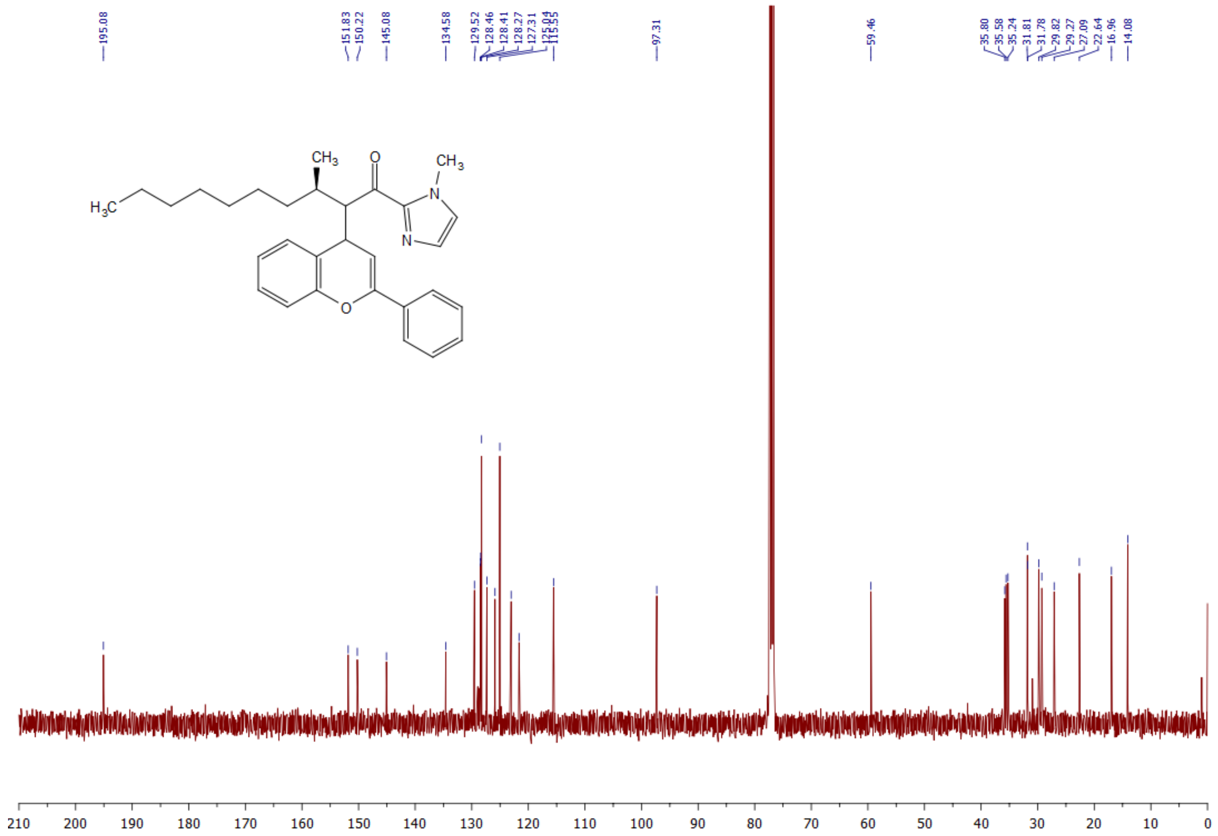

^{13}C NMR spectrum of compound **6 (diastereomer 3)**.

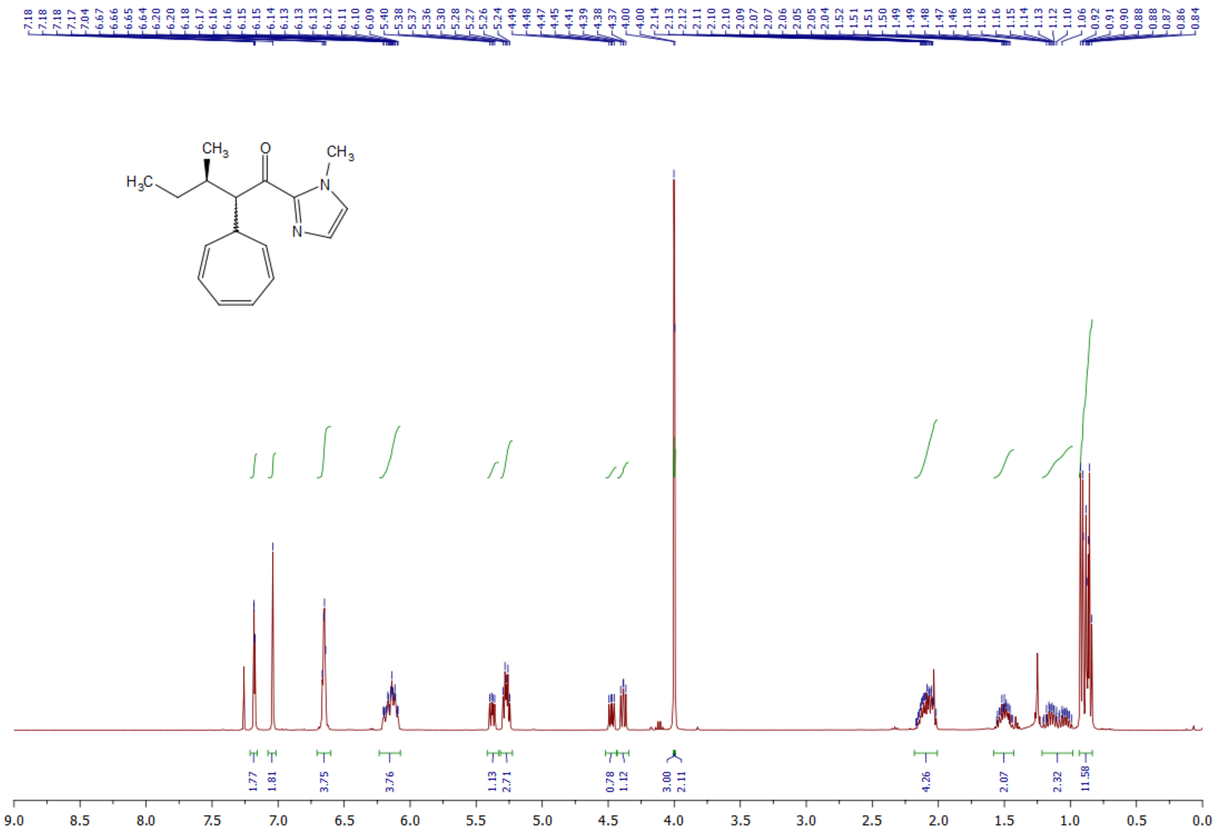

^{1}H NMR spectrum of compound **3b**.

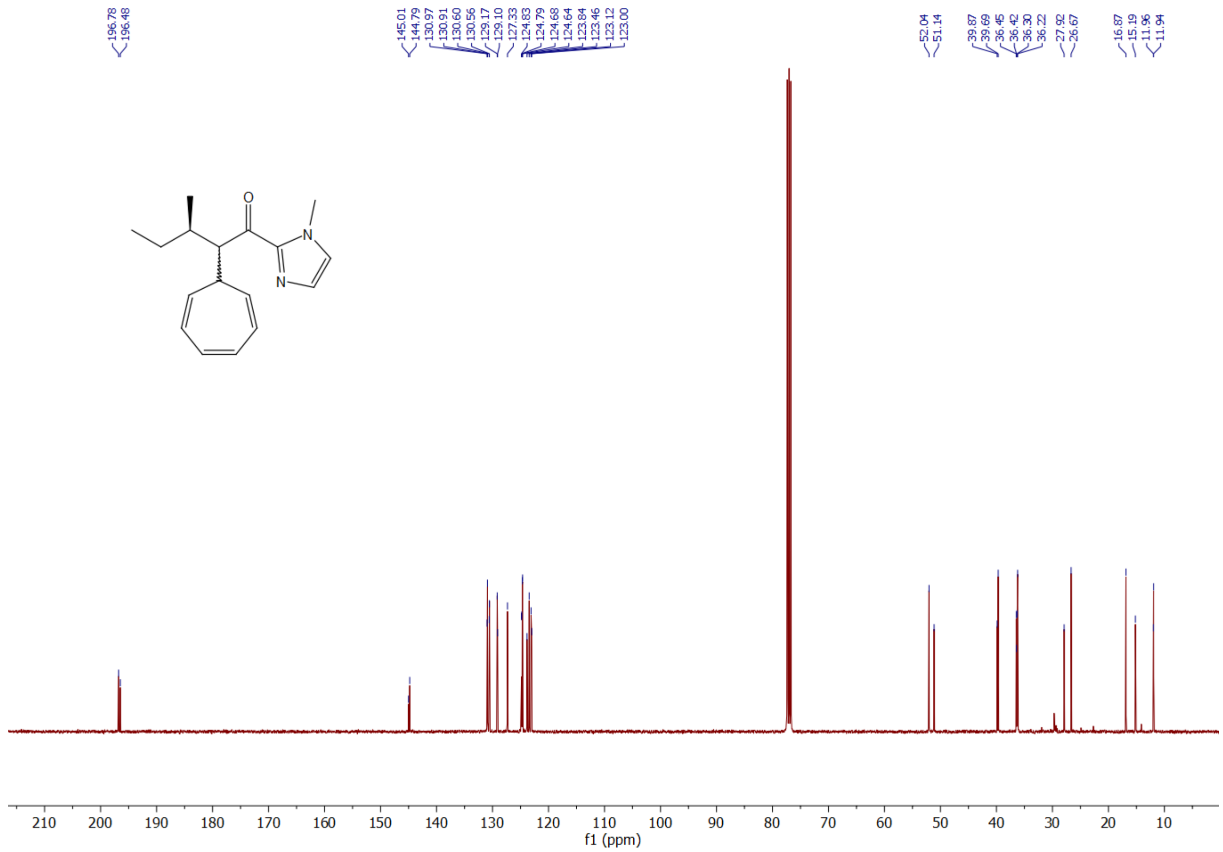

^{13}C NMR spectrum of compound **3b**.

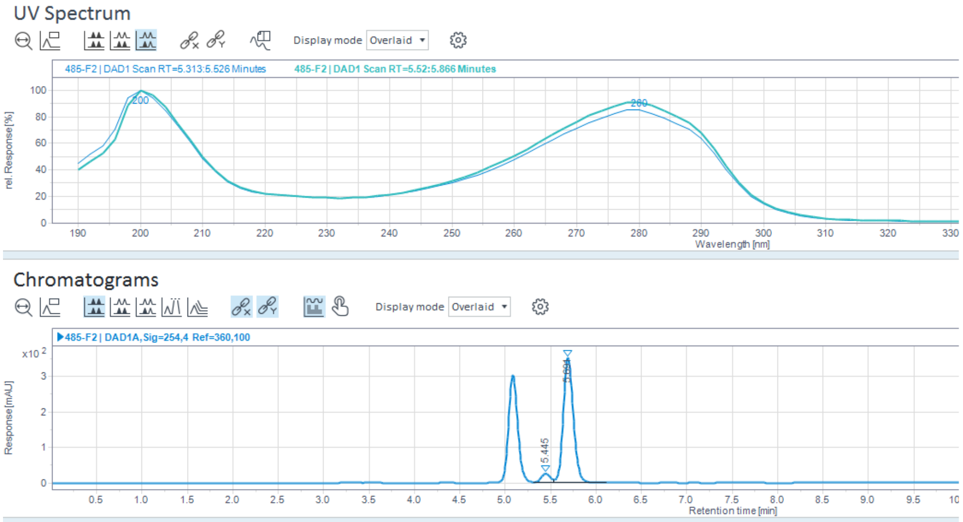

Injection Results

Peaks | Summary

#	RT (min)	Area (mAU·s)	Area%	Height (mAU)	Height%
1	5.445	187.034	6.447	25.866	6.86
2	5.691	2714.252	93.553	351.414	93.14

HPLC chromatogram of compound **3b** (non-racemic).

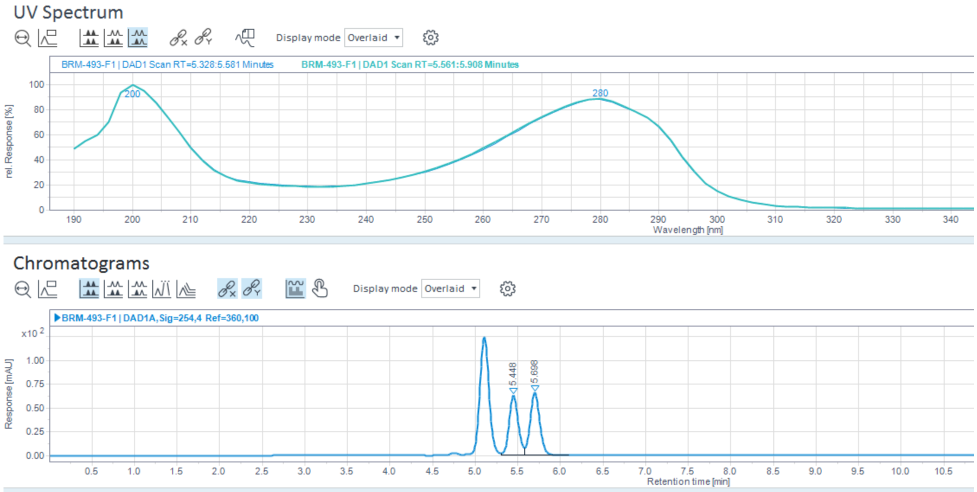

Injection Results

Peaks | Summary

#	RT (min)	Area (mAU·s)	Area%	Height (mAU)	Height%
1	5.448	461.563	47.573	62.405	48.76
2	5.698	508.650	52.427	65.579	51.24

HPLC chromatogram of compound **3b** (racemic).

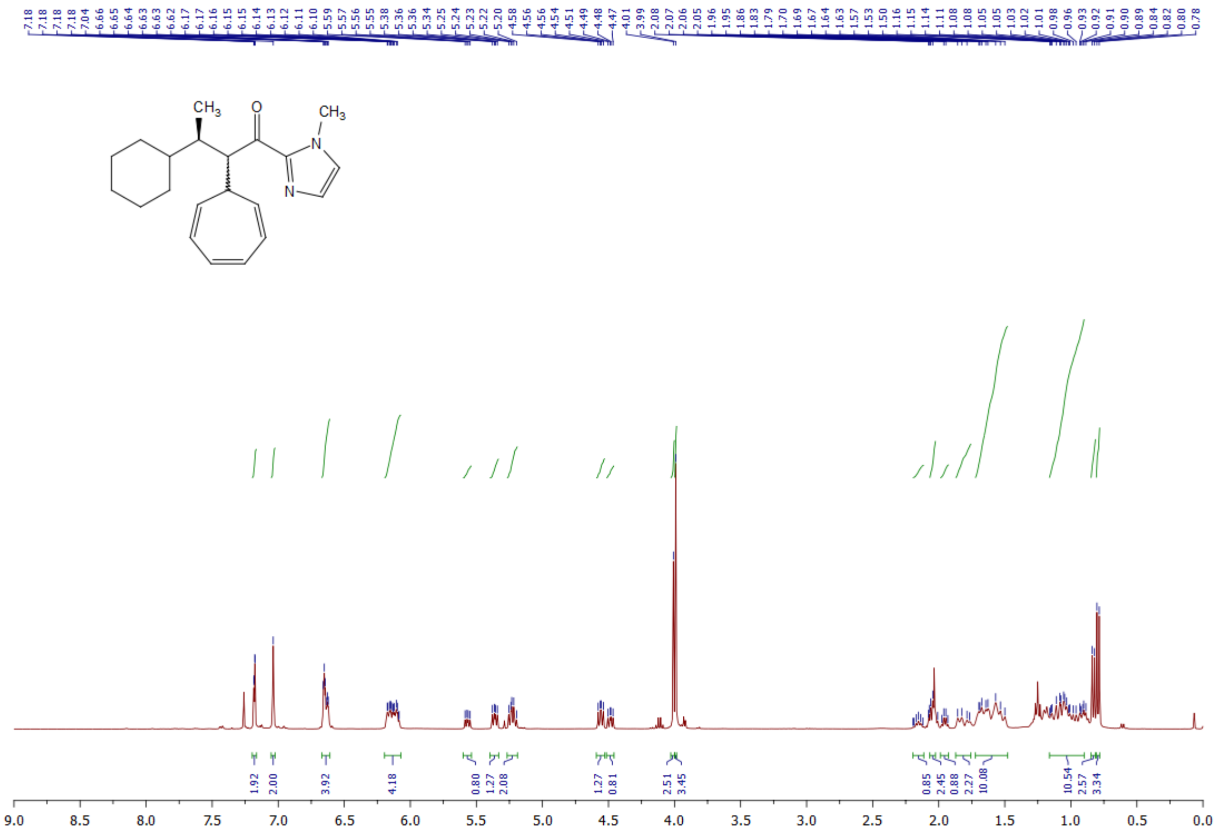

^{1}H NMR spectrum of compound **3c**.

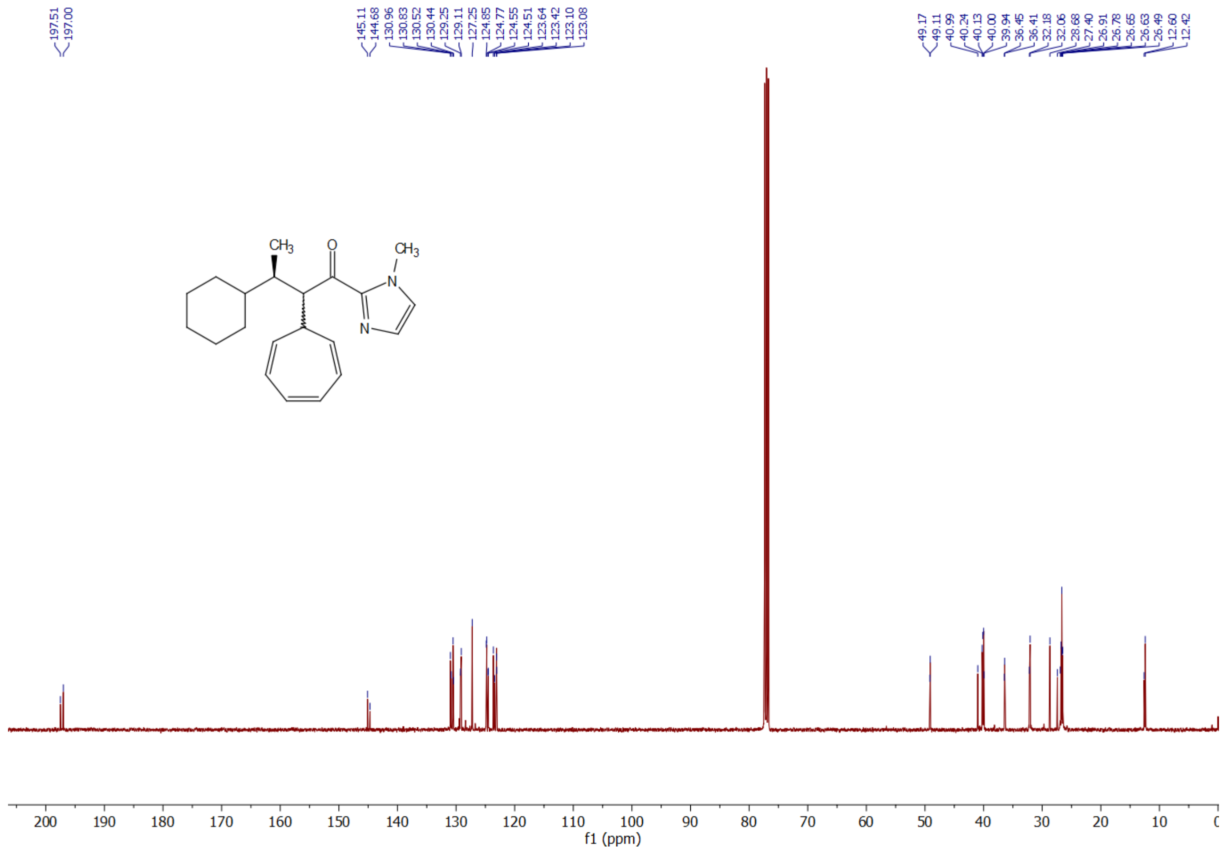

^{13}C NMR spectrum of compound **3c**.

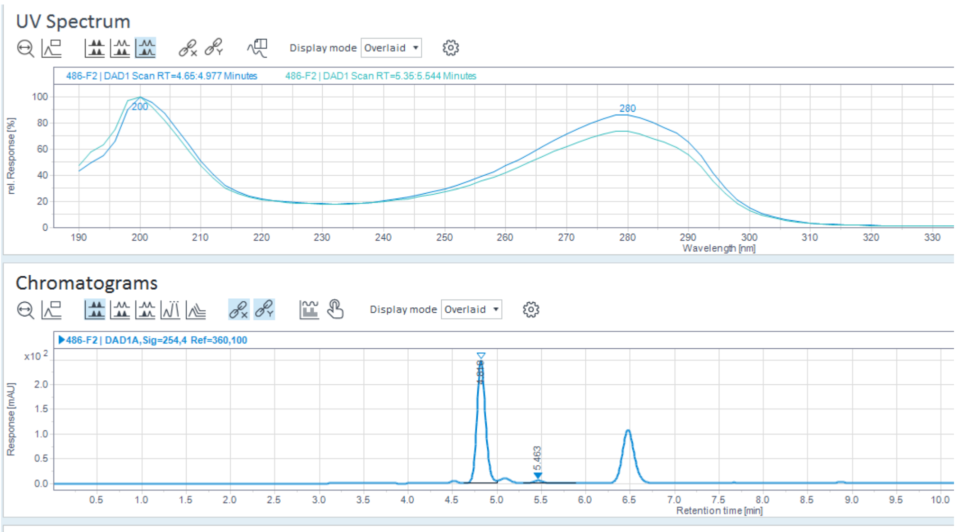

Injection Results

Peaks | Summary

#	RT (min)	Area (mAU·s)	Area%	Height (mAU)	Height%
1	4.818	1652.829	97.263	248.532	97.75
2	5.463	46.516	2.737	5.711	2.25

HPLC chromatogram of compound **3c** (non-racemic).

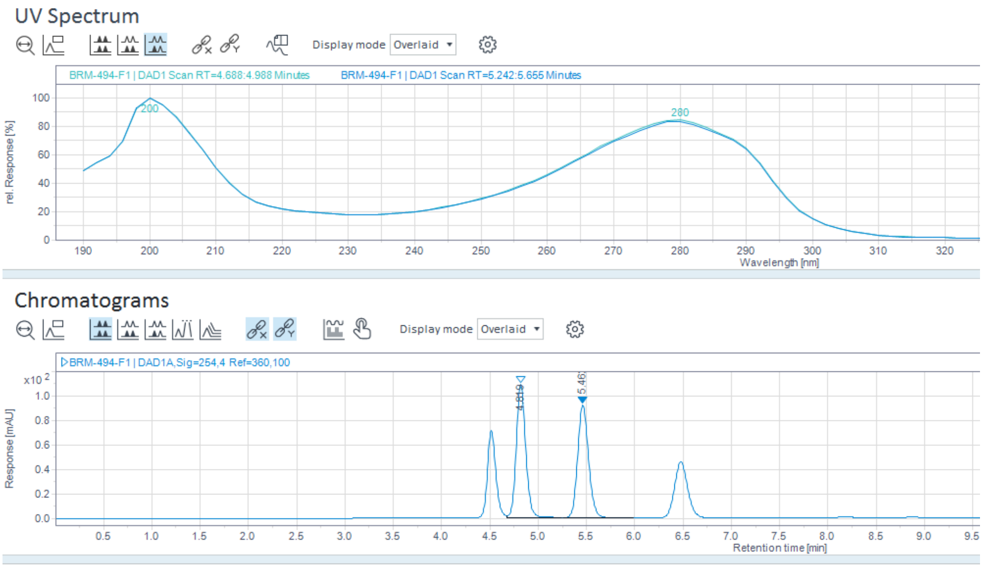

Injection Results

Peaks | Summary

#	RT (min)	Area (mAU·s)	Area%	Height (mAU)	Height%
1	4.819	739.790	50.657	109.488	54.19
2	5.462	720.612	49.343	92.572	45.81

HPLC chromatogram of compound **3c** (racemic).

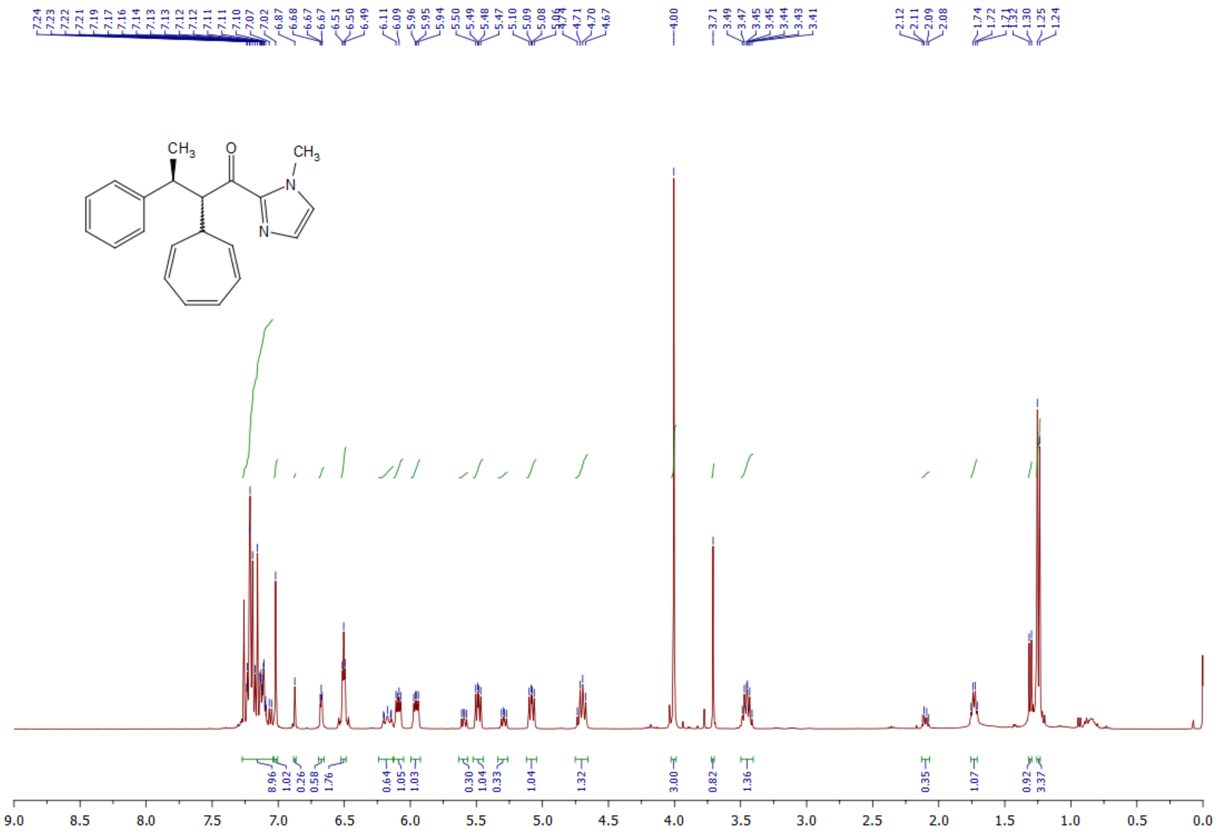

^{1}H NMR spectrum of compound **3d**.

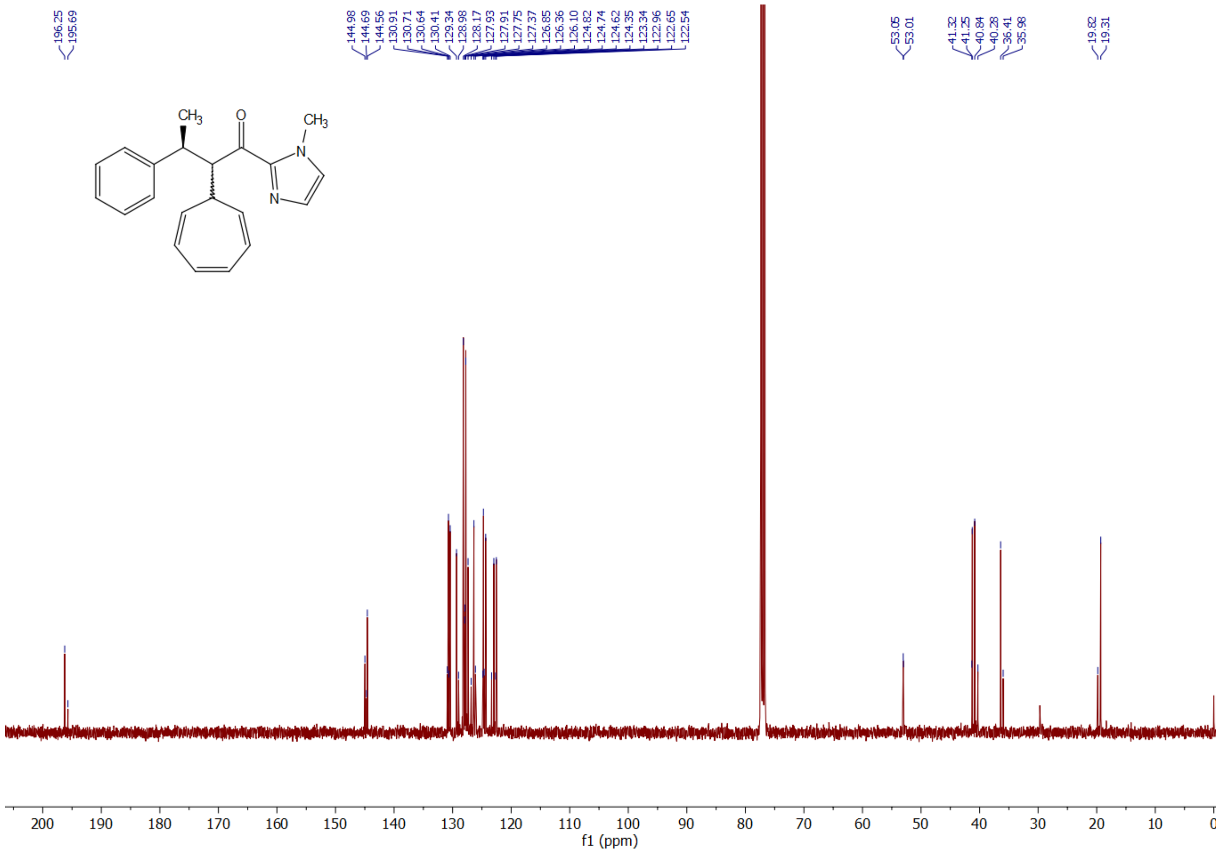

^{13}C NMR spectrum of compound **3d**.

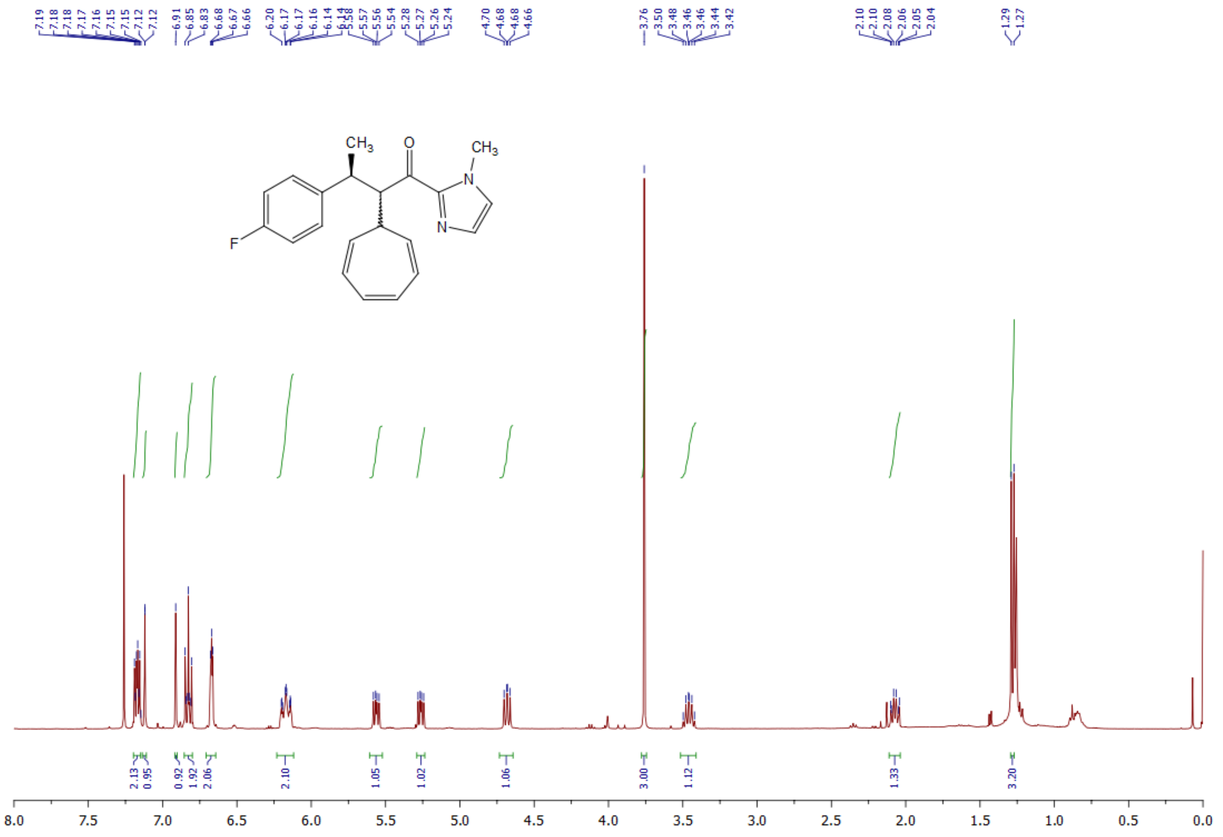

^{1}H NMR spectrum of compound **3e (1 diast.)**.

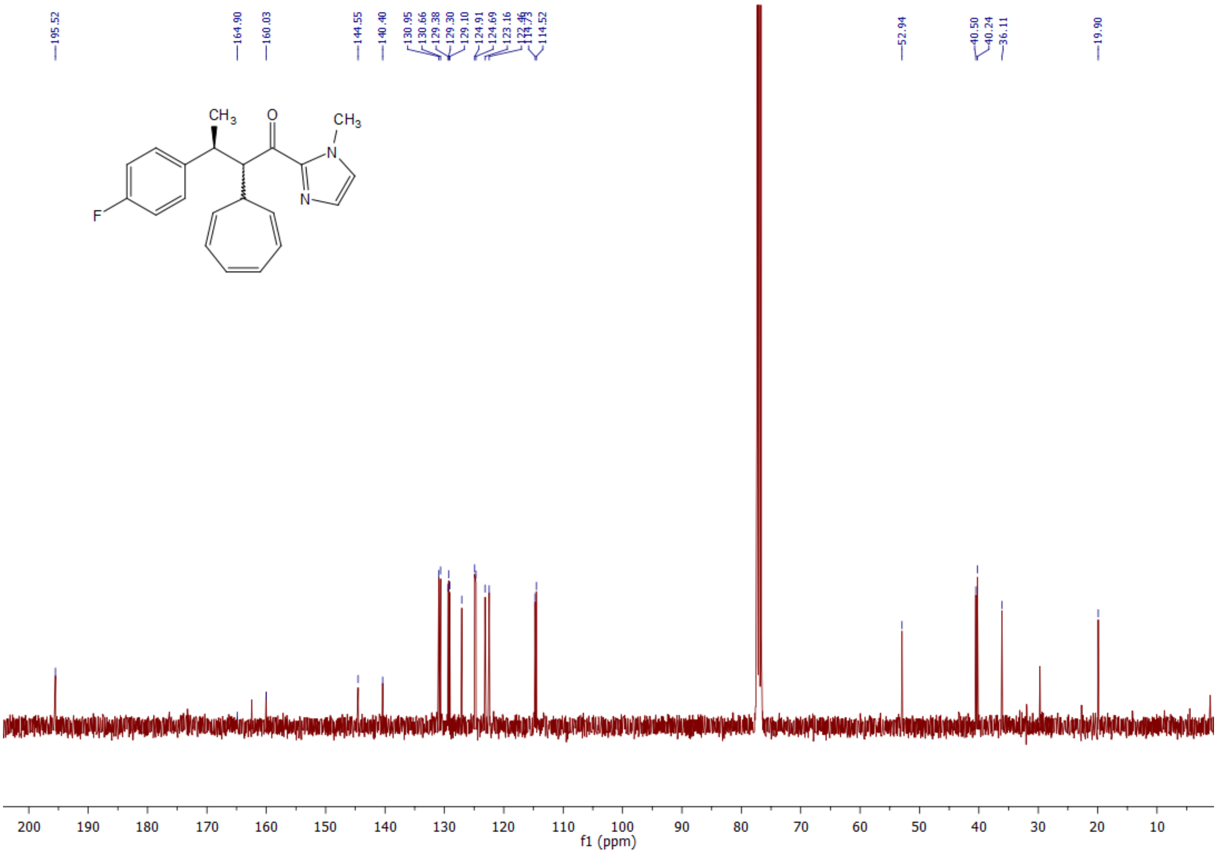

^{13}C NMR spectrum of compound **3e (1 diast)**.

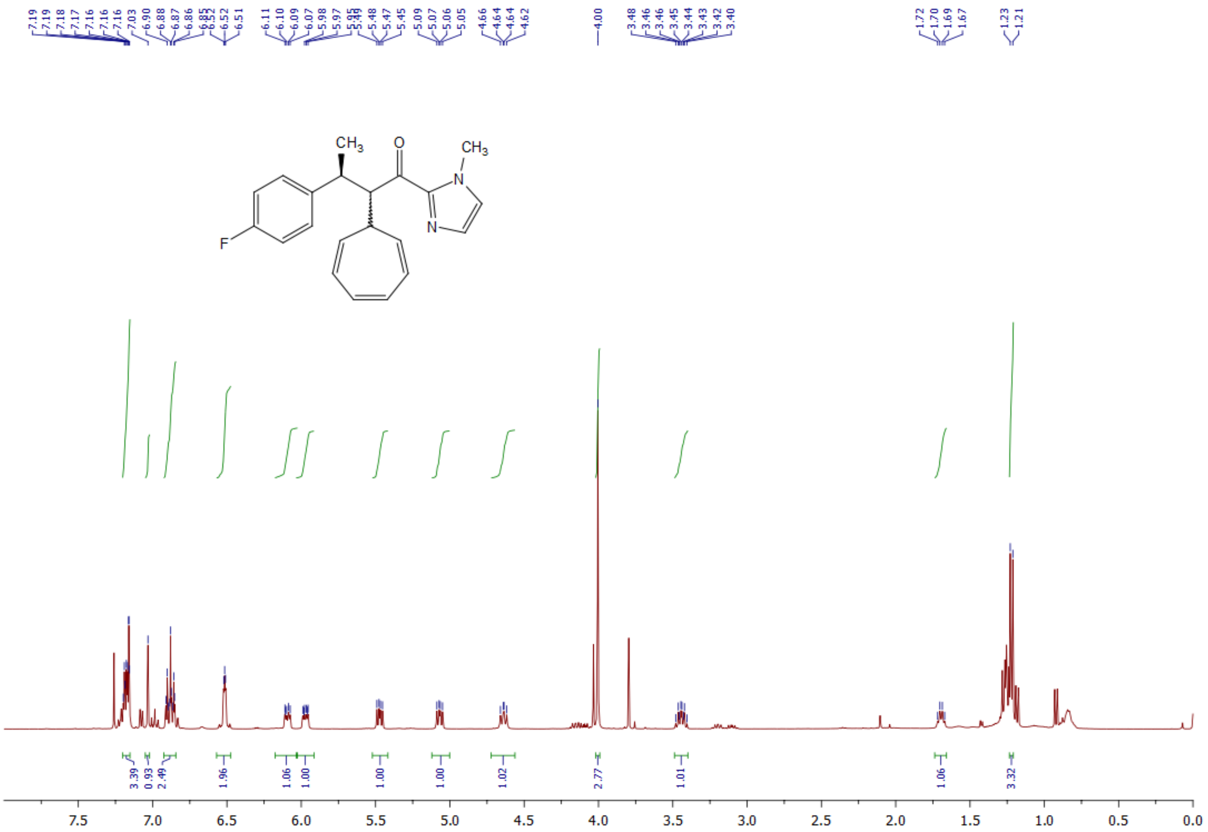

^{1}H NMR spectrum of compound **3e (2 diast.)**.

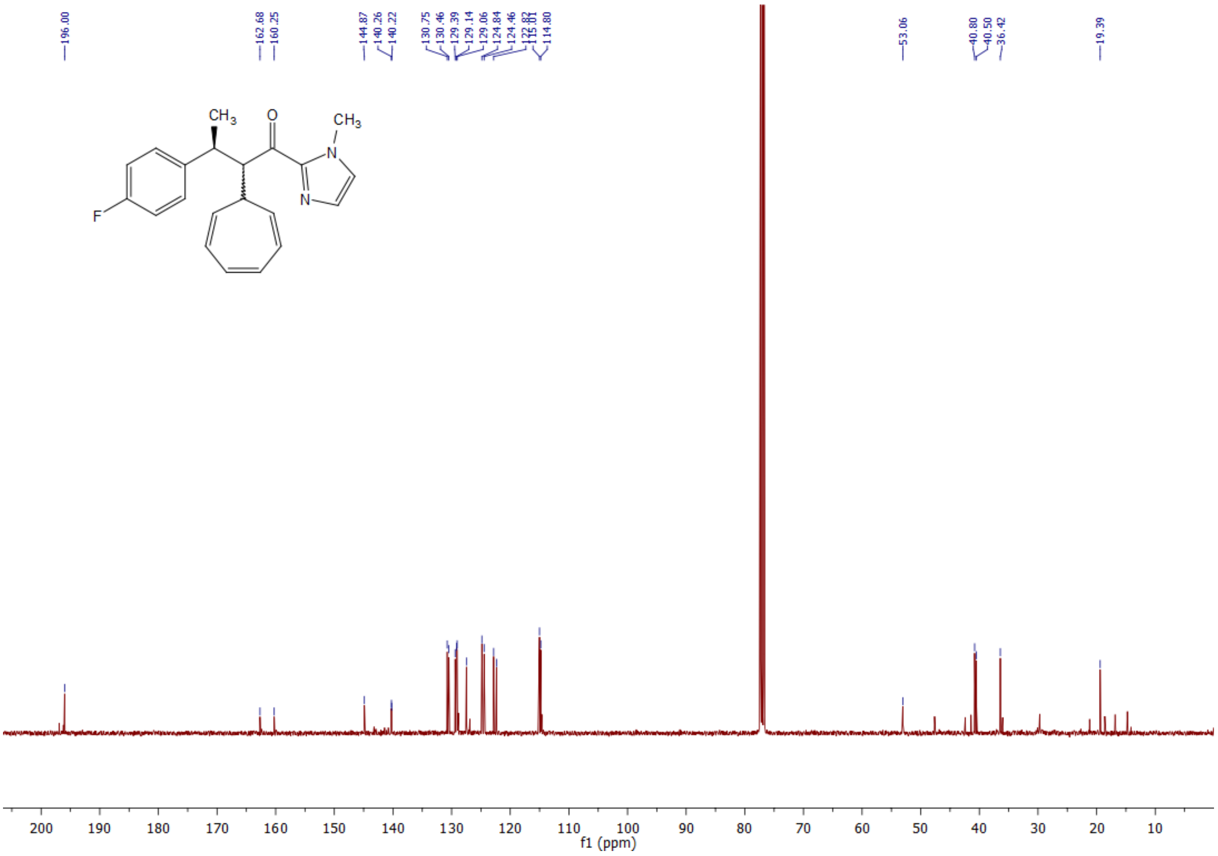

^{13}C NMR spectrum of compound **3e (2 diast)**.

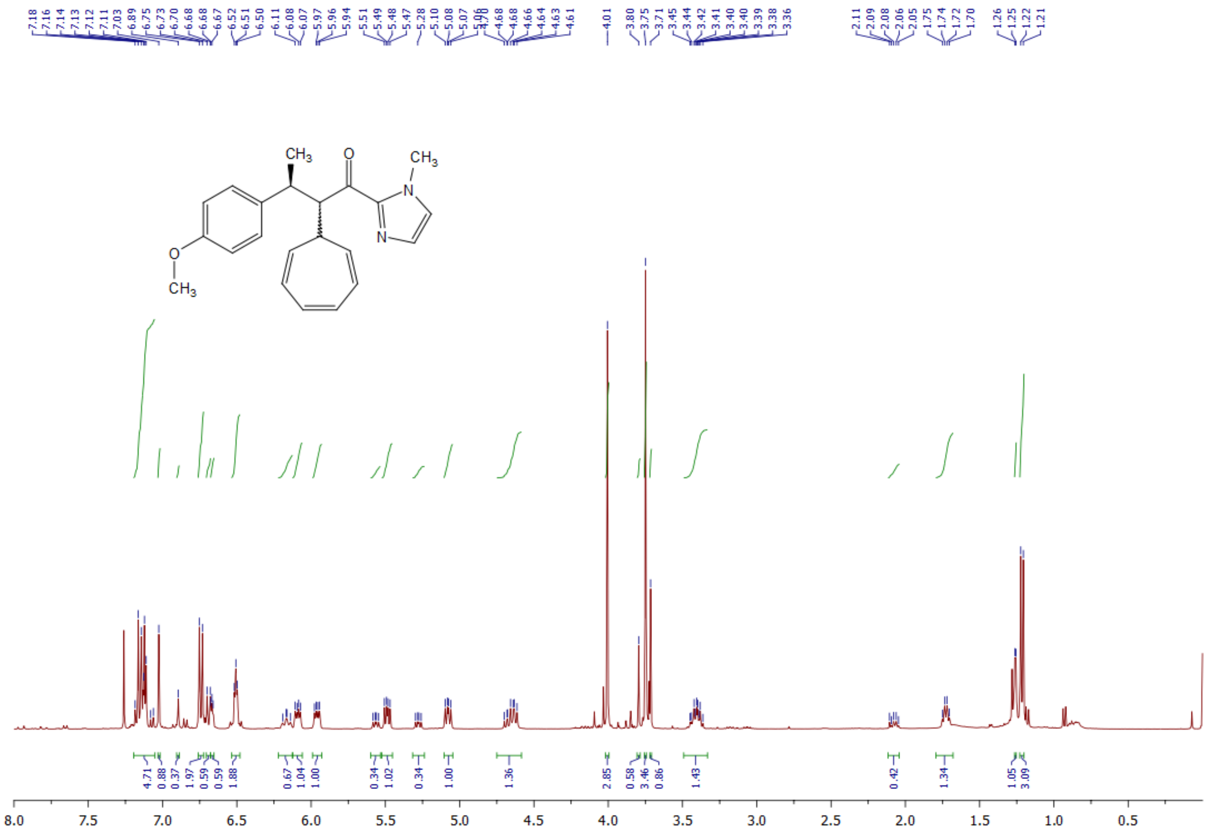

^{1}H NMR spectrum of compound **3f**.

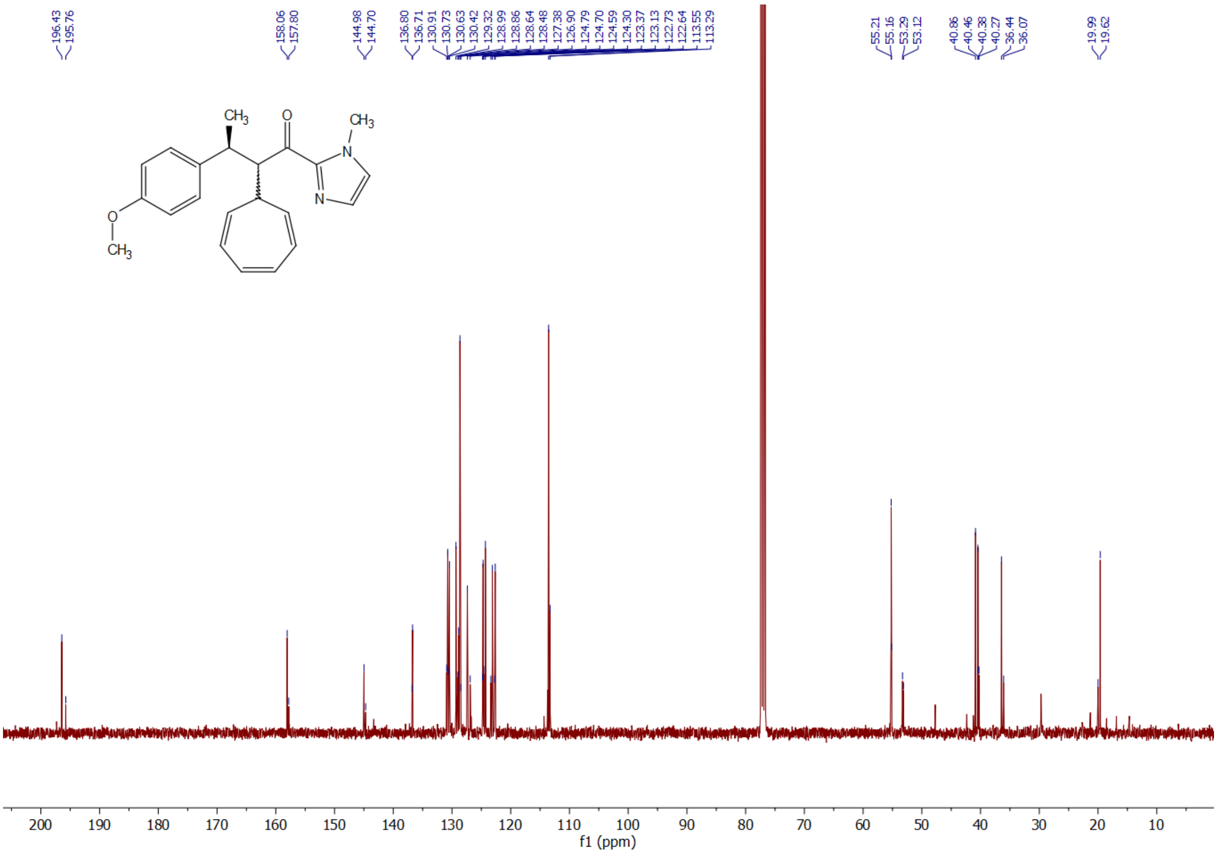

^{13}C NMR spectrum of compound **3f**.

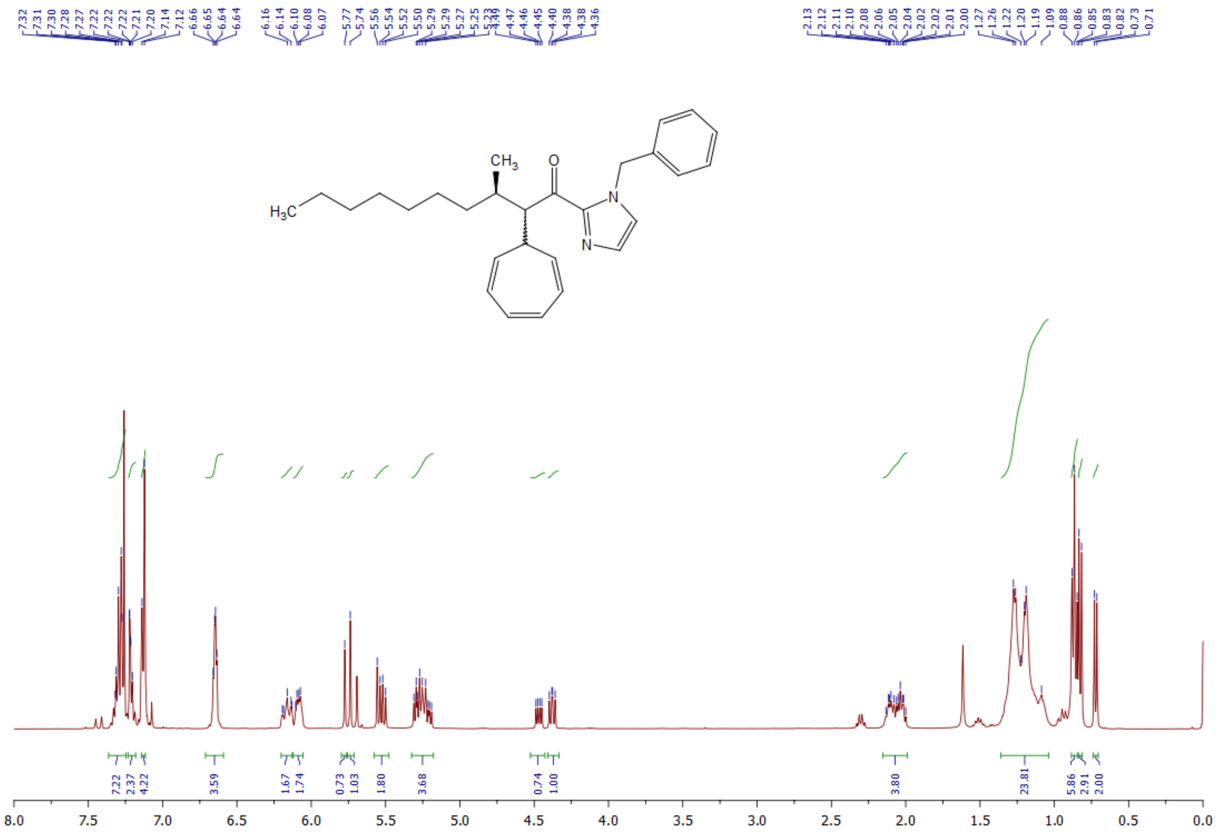

^{1}H NMR spectrum of compound **3g**.

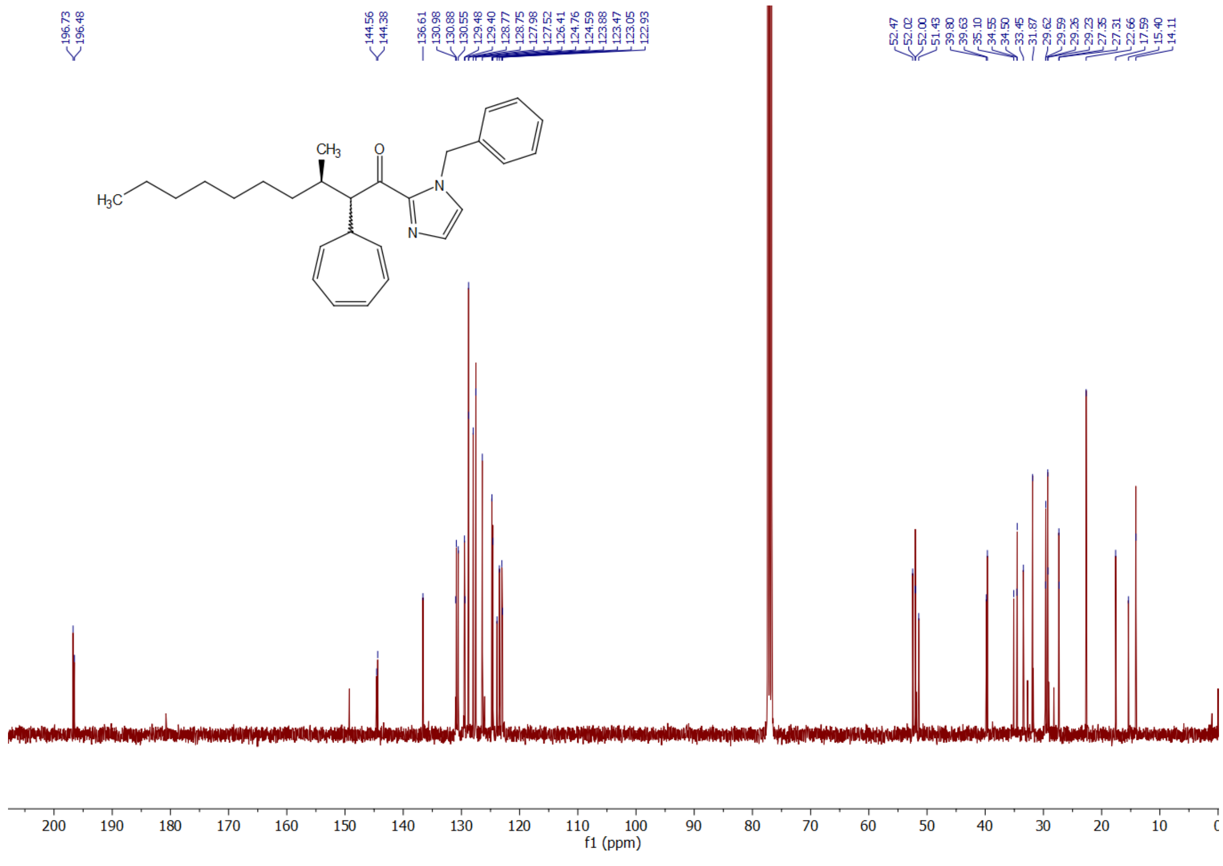

^{13}C NMR spectrum of compound **3g**.

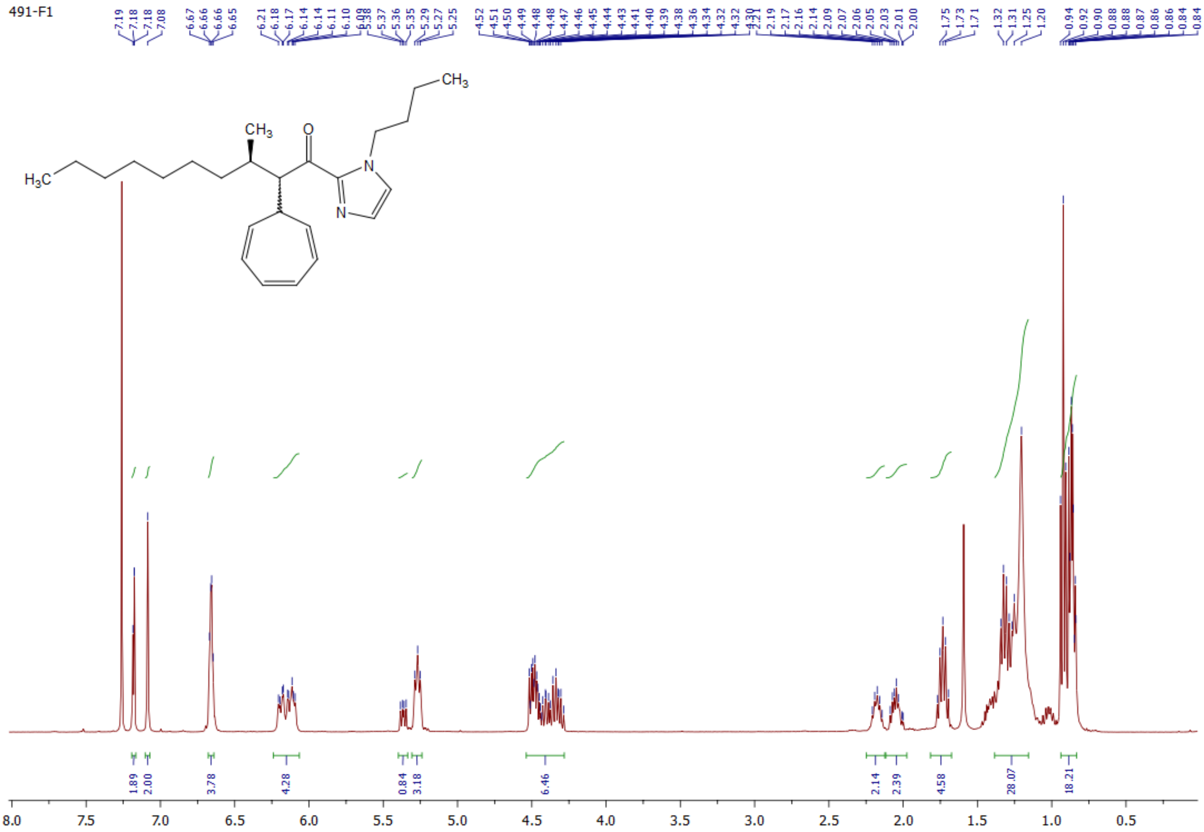

^{1}H NMR spectrum of compound **3h**.

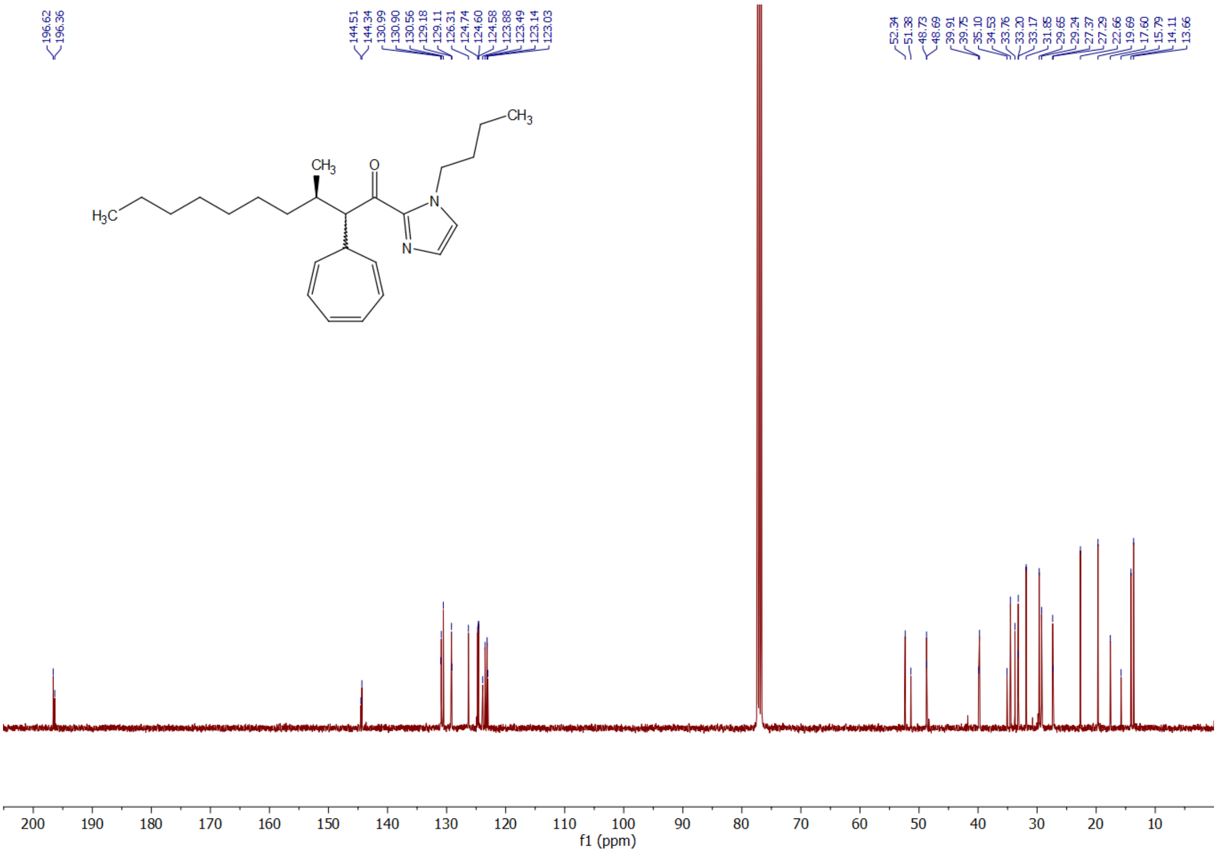

^{13}C NMR spectrum of compound **3h**.

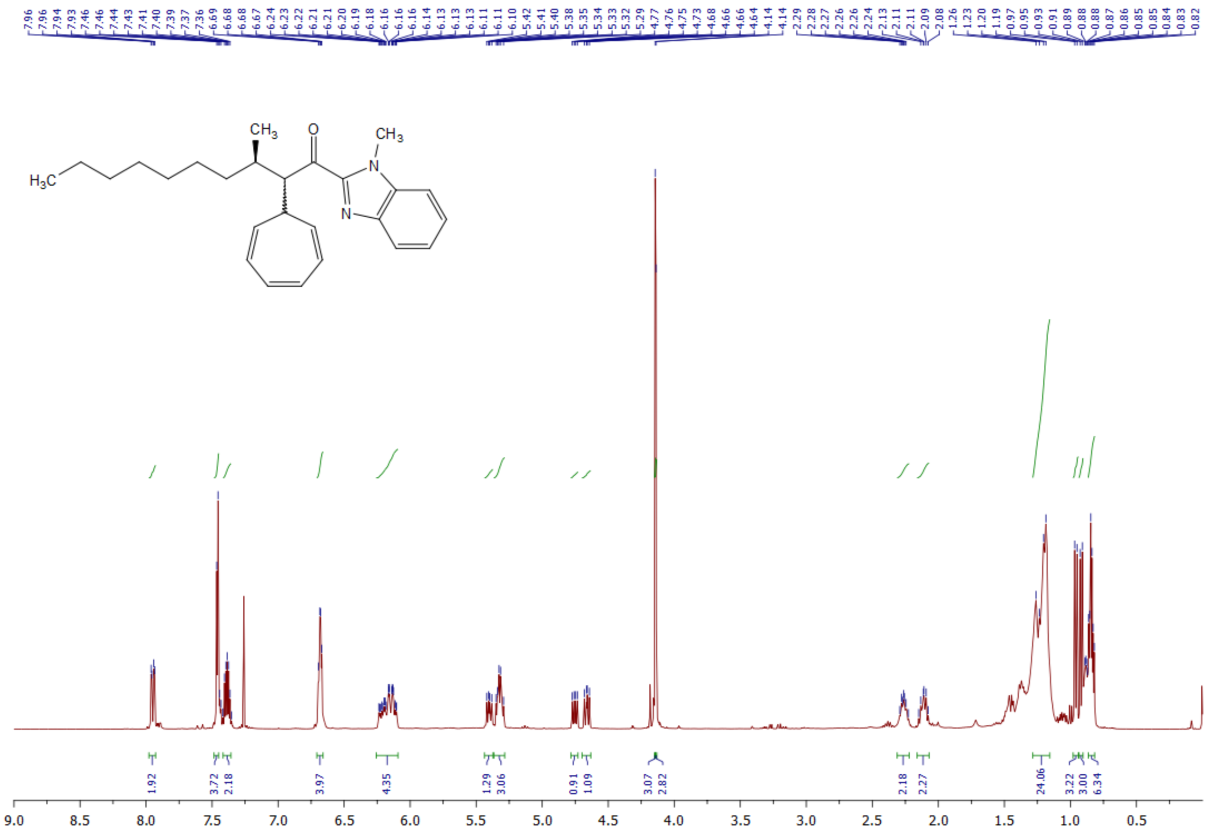

^{1}H NMR spectrum of compound **3i**.

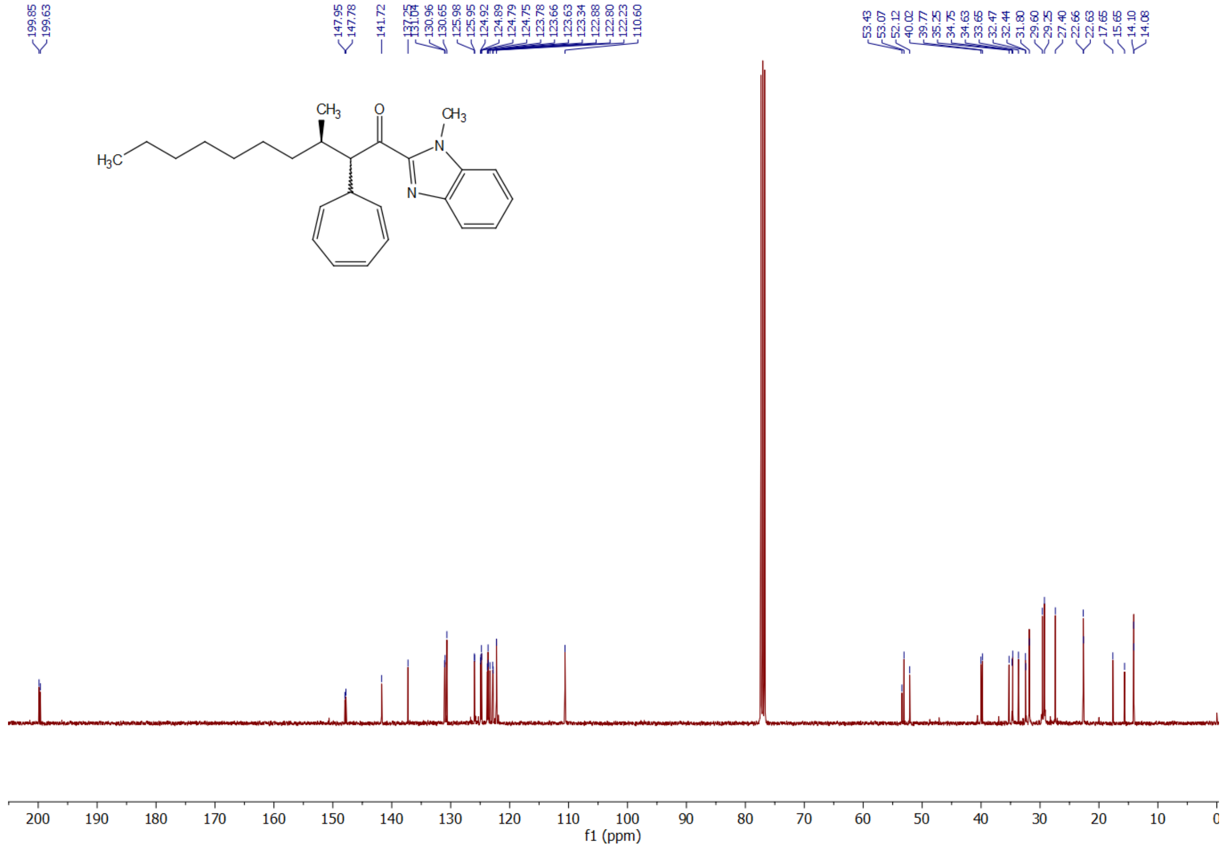

^{13}C NMR spectrum of compound **3i**.

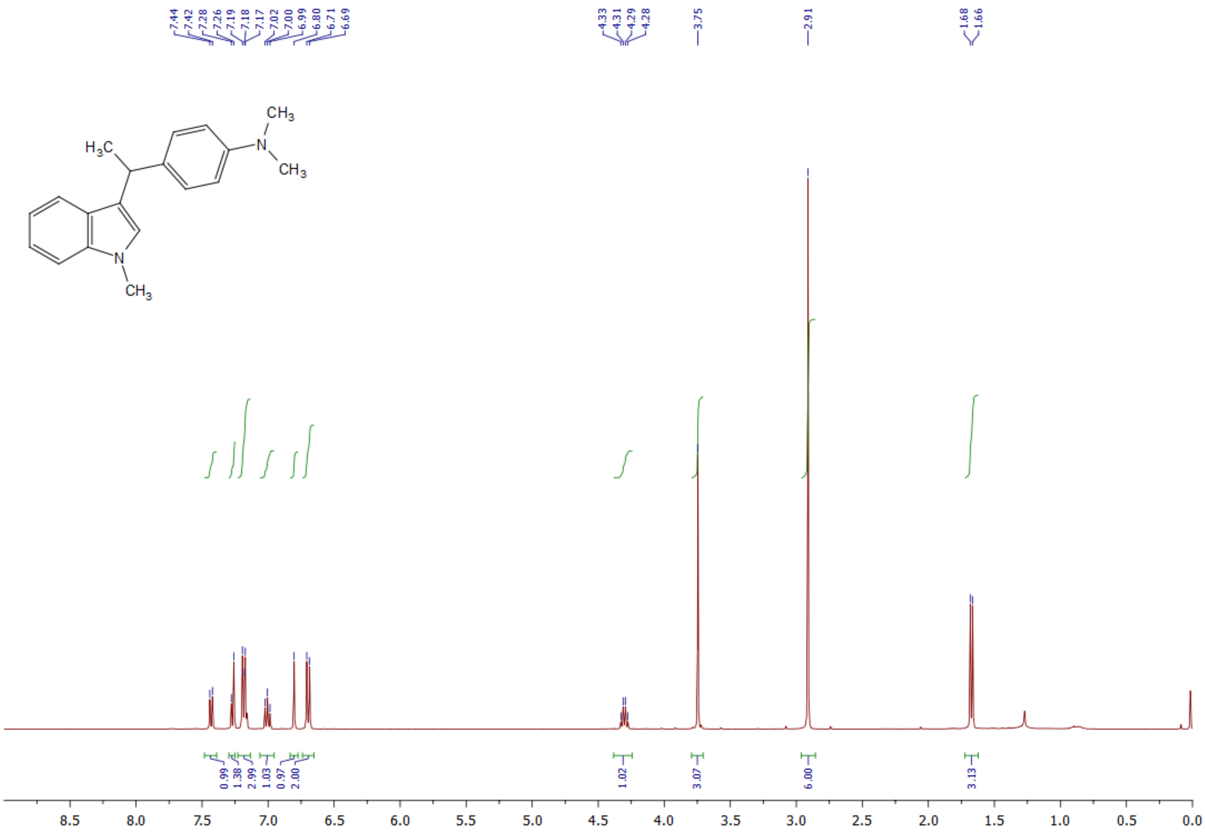

^{1}H NMR spectrum of compound **S1**.

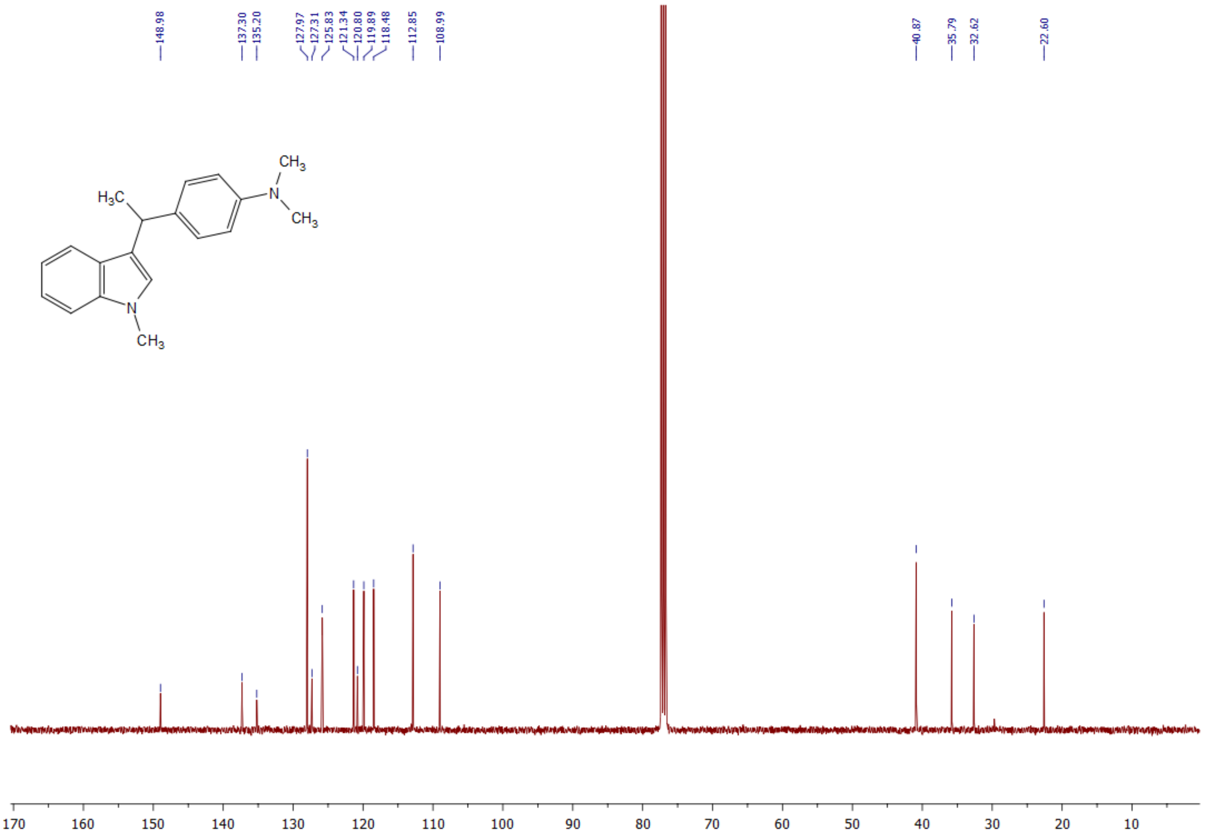

^{13}C NMR spectrum of compound **S1**.

6. CD spectra

CD spectra were measured in MeCN solutions.

CD of (*R,R*)-**4**

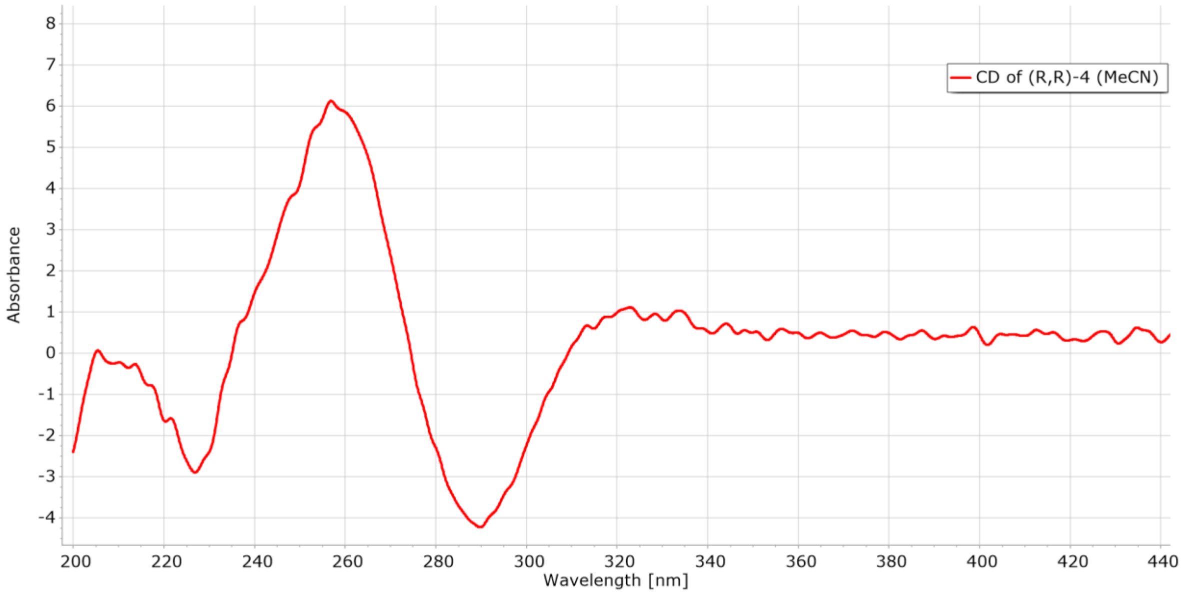

CD of (*S,R*)-**4**

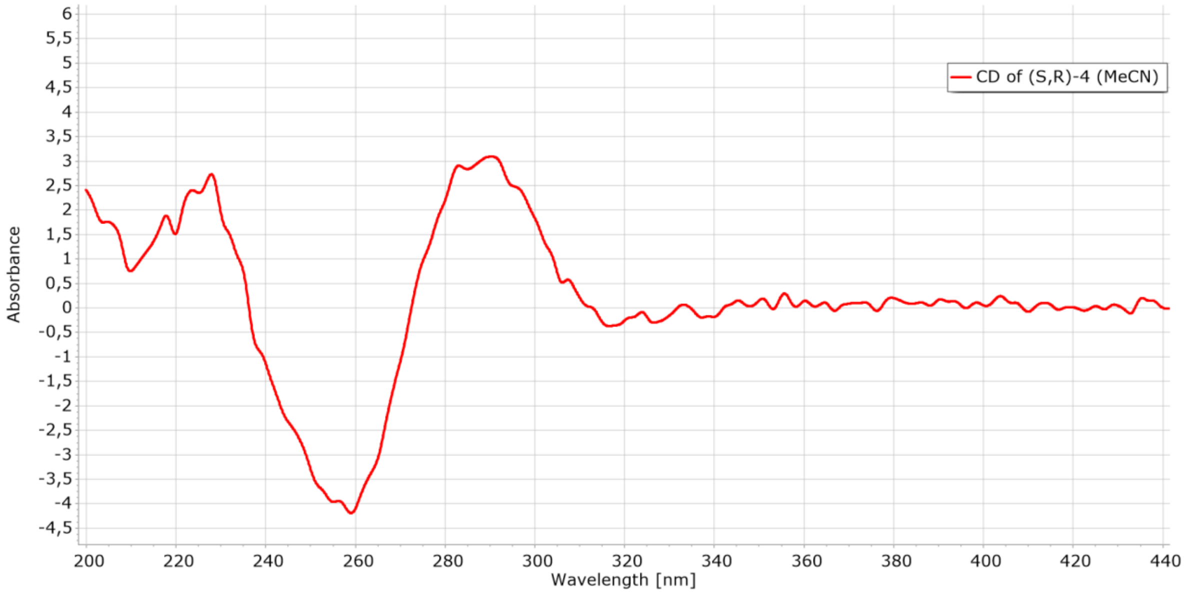

7. Details of DFT calculations

General

Initial structures were built and pre-optimized using the semi-empirical RM1 method and HF/3-21G calculations [7]. Conformational analyses were performed using Spartan 18. DFT calculations were realized using the Turbomole program package [8,9]. Geometric optimizations were performed using B3LYP-D3(BJ) [10-14], or PBEh-3c functional [15]. Geometrical optimizations were performed with def2-mSVP or SV(P) basis set [16]. Energies were refined at M06-2X/def2-TZVP [17], or ωB97X-D4/def2-TZVPP level [18-20]. CD spectra were calculated by TD DFT calculations using the Tamm-

Dancoff approximation at CAM-B3LYP/def2-TZVP [21], B97-3c/def2-mTZVP [22], and PBE0-D4/def2-SVP [23,24] level in Turbomole program [25,26]. Enolate properties were calculated at ωB97X-D4/def2-TZVPP and TZVPPD level.

Calculations of CD spectra

Calculated spectra for two most populated conformers of (*R,R*)-**4**

B97-3c/mTZVP (MeCN), conformer 1

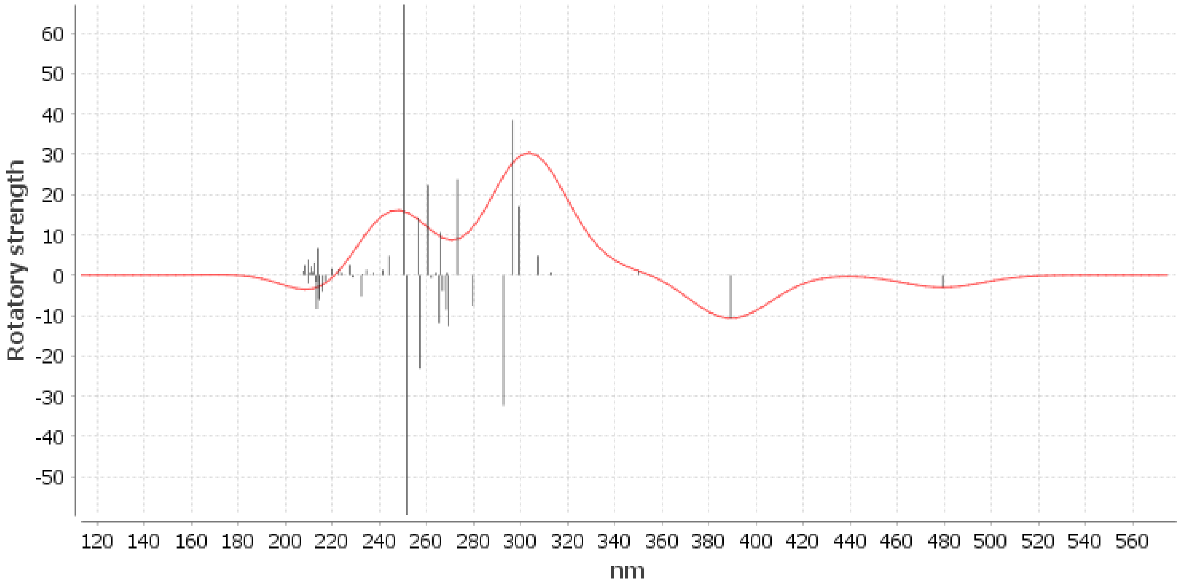

B97-3c/mTZVP (MeCN), conformer 2

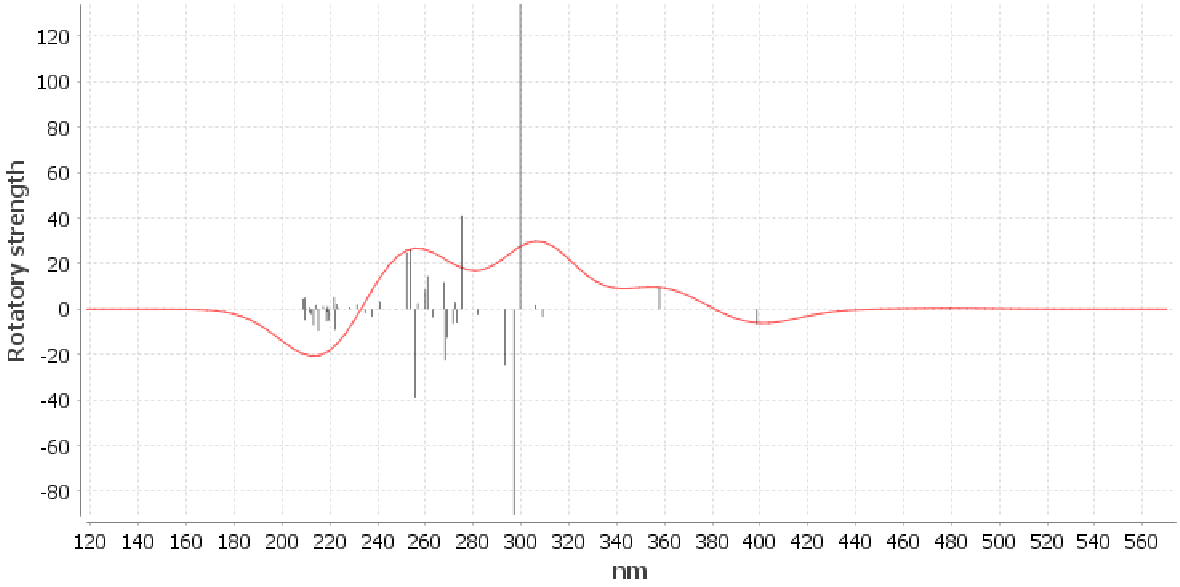

CAM-B3LYP/TZVP, conformer 1

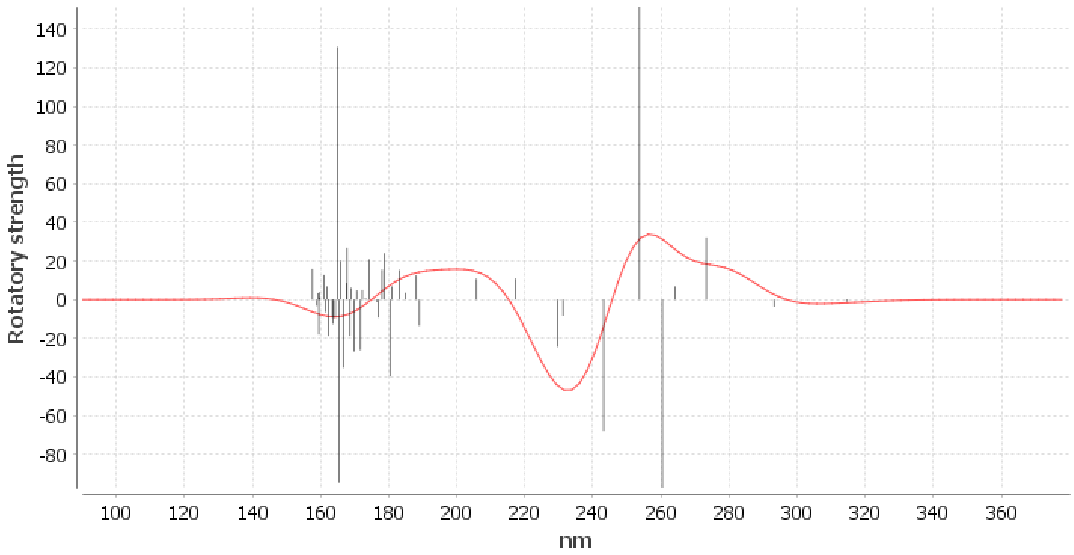

CAM-B3LYP/TZVP, conformer 2

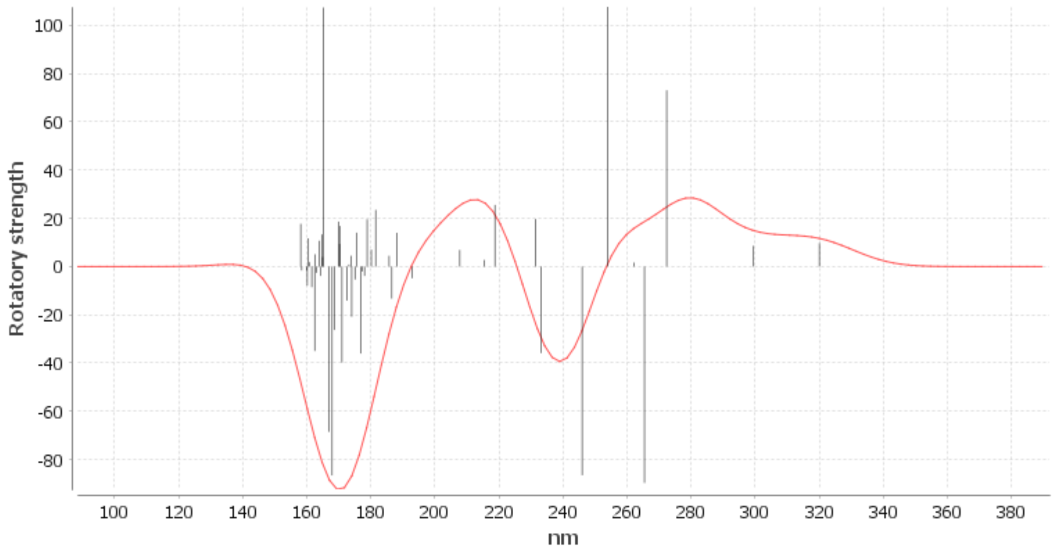

PBE0-D4/SVP (MeCN), conformer 1

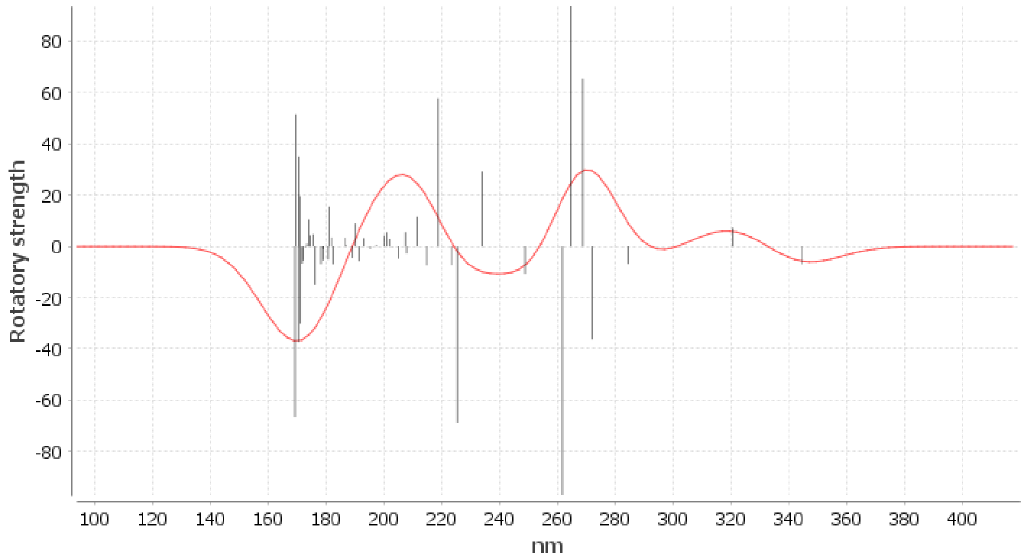

PBE0-D4/SVP (MeCN), conformer 2

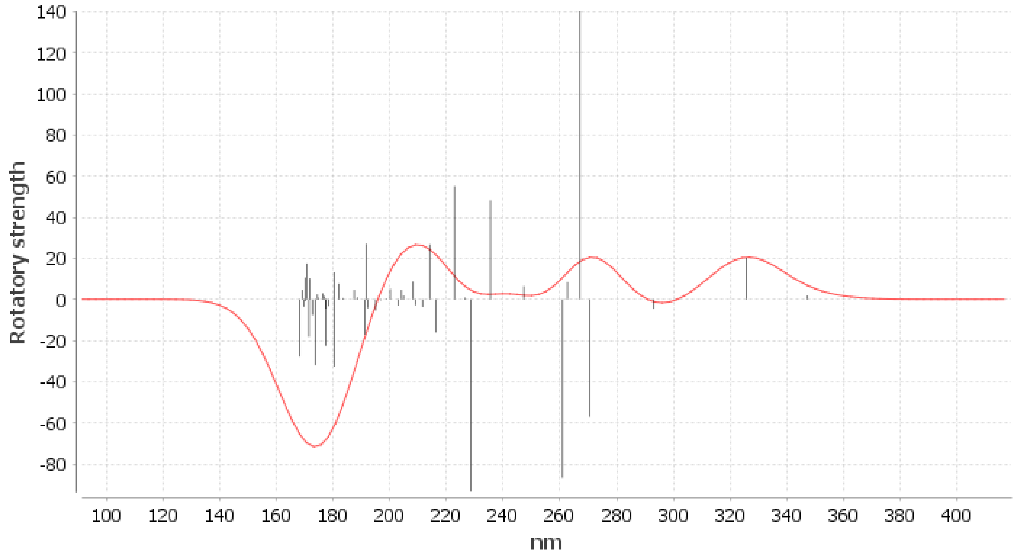

PBE0-D4/SVP, conformer 1

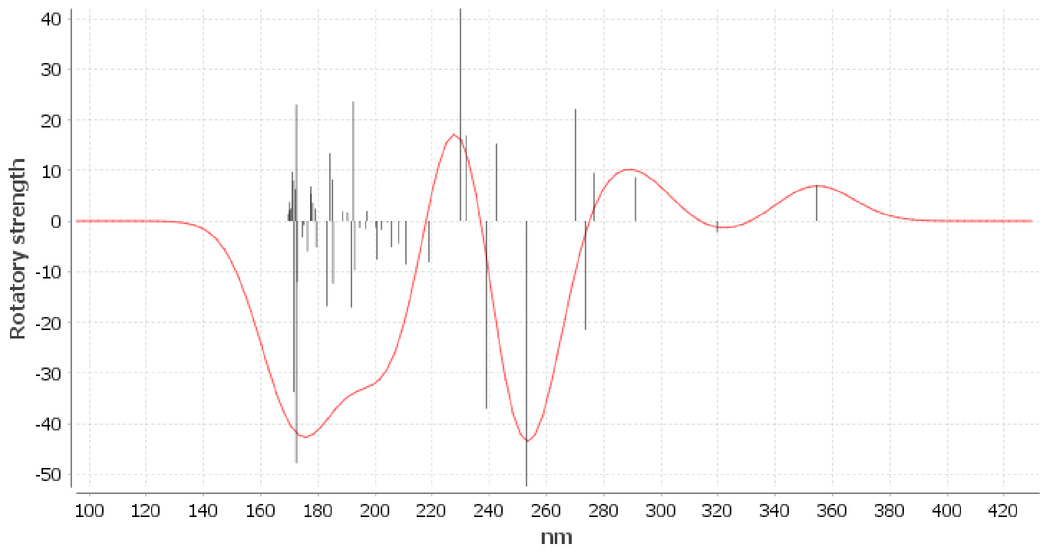

PBE0-D4/SVP, conformer 2

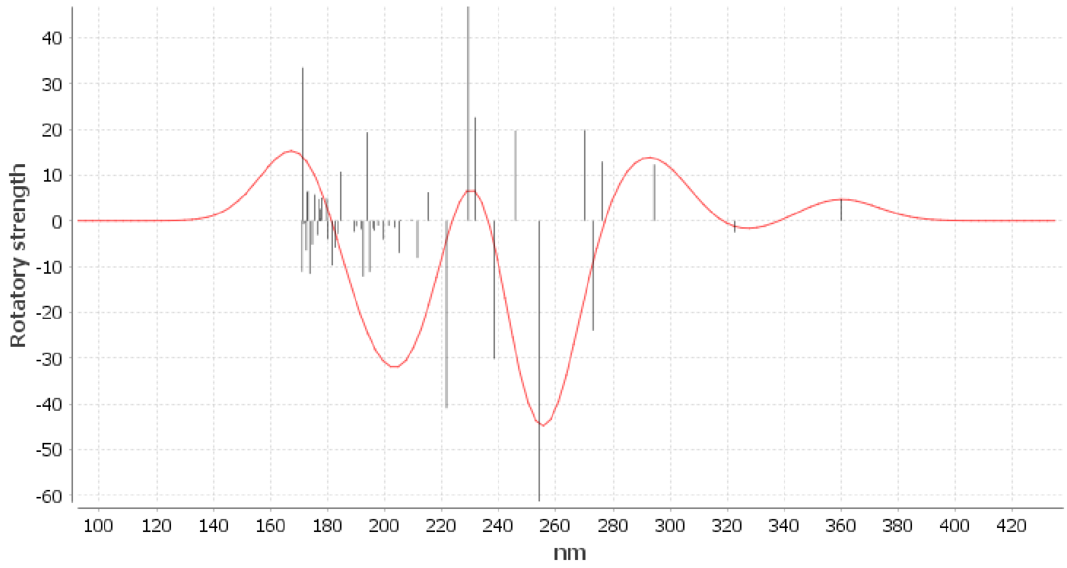

Calculated spectra for two most populated conformers of (*S*,*R*)-**4**

B97-3c/mTZVP (MeCN), conformer 1

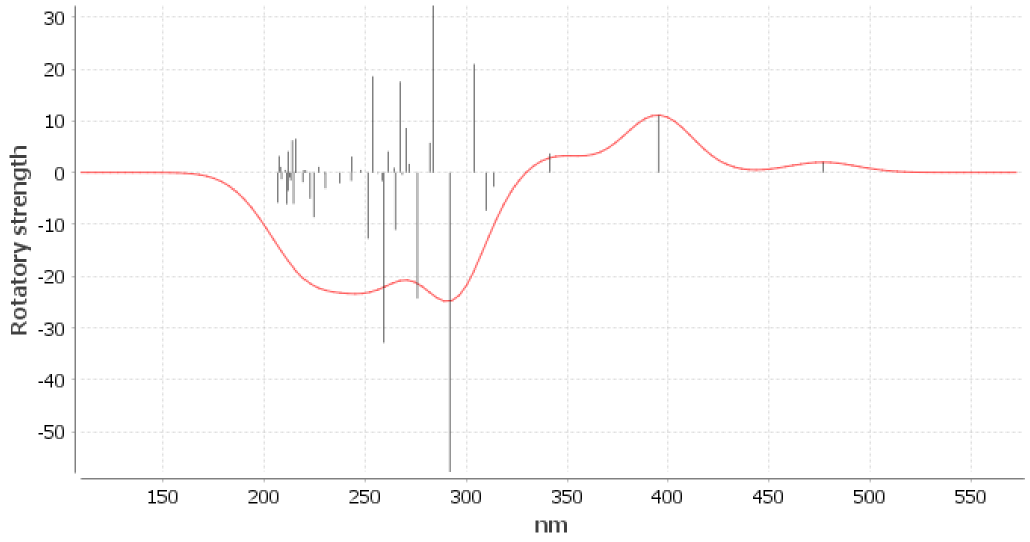

B97-3c/mTZVP (MeCN), conformer 2

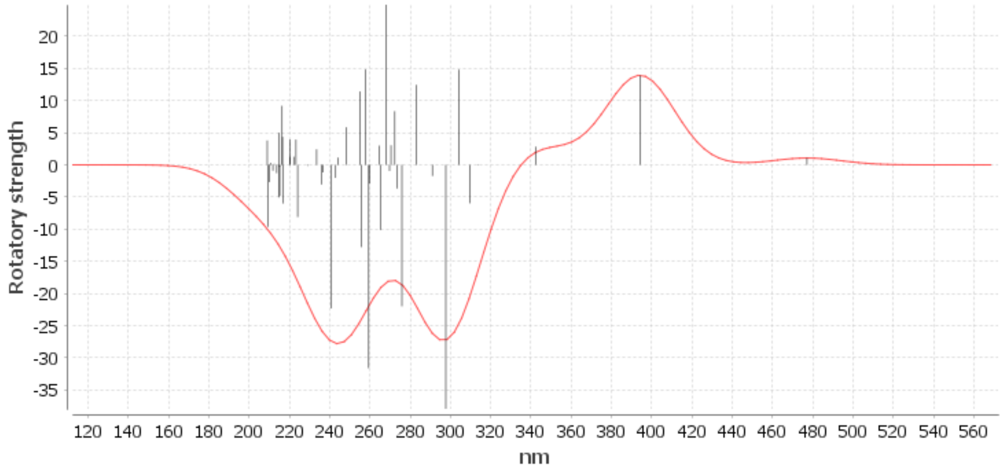

PBE0-D4/SVP (MeCN), conformer 1

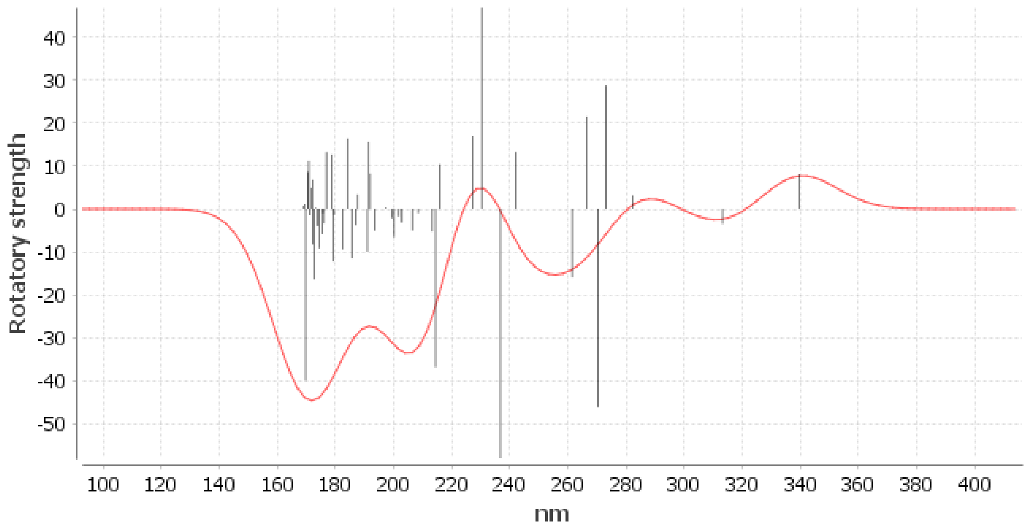

PBE0-D4/SVP (MeCN), conformer 2

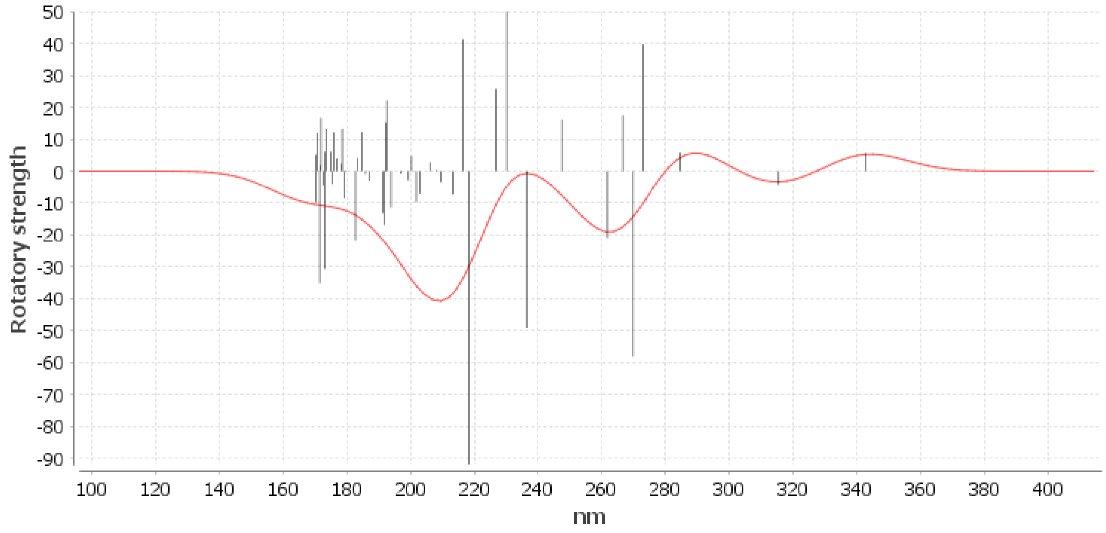

PBE0-D4/SVP, conformer 1

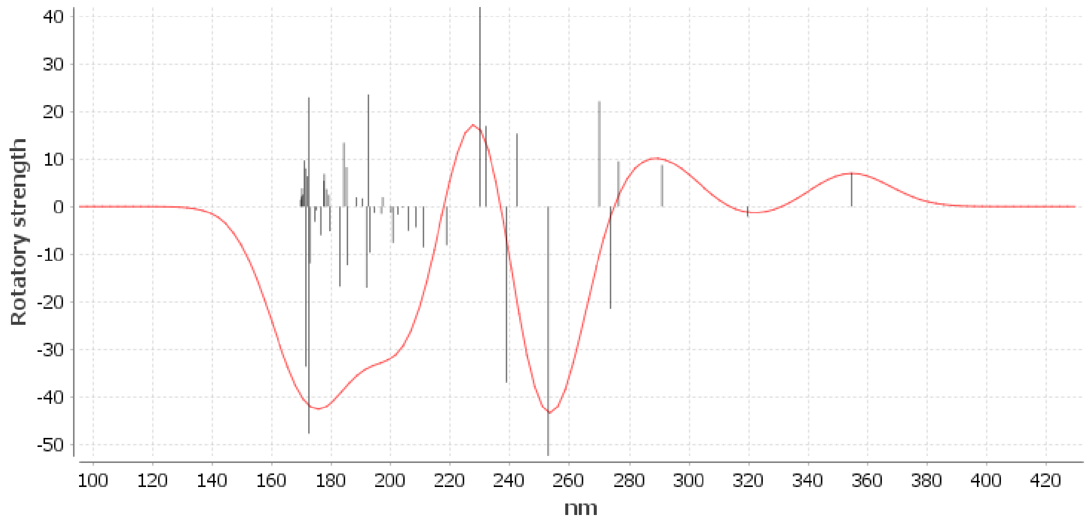

PBE0-D4/SVP, conformer 2

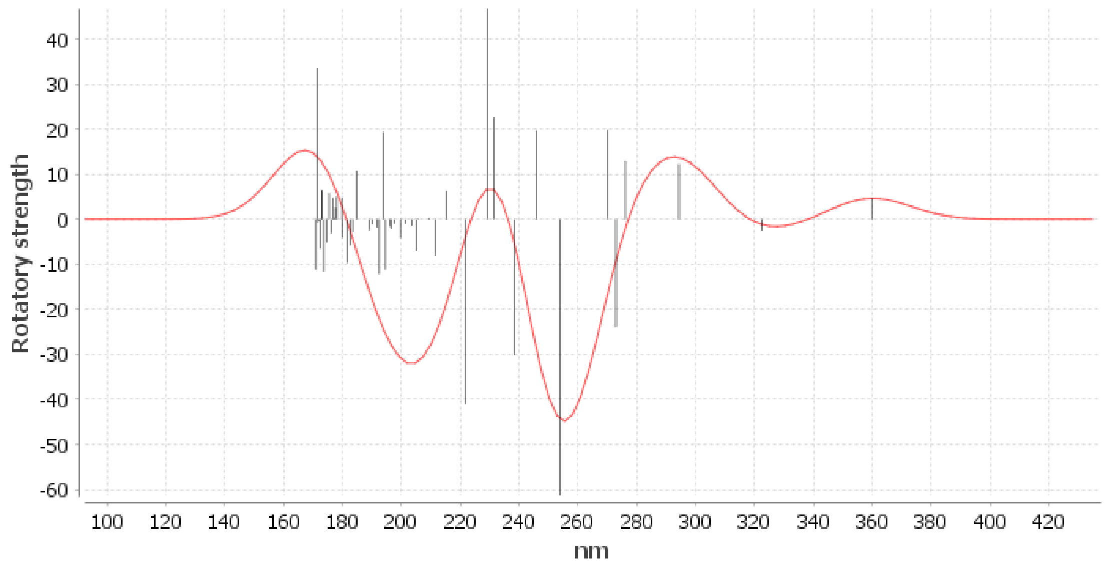

CAM-B3LYP/TZVP, conformer 1

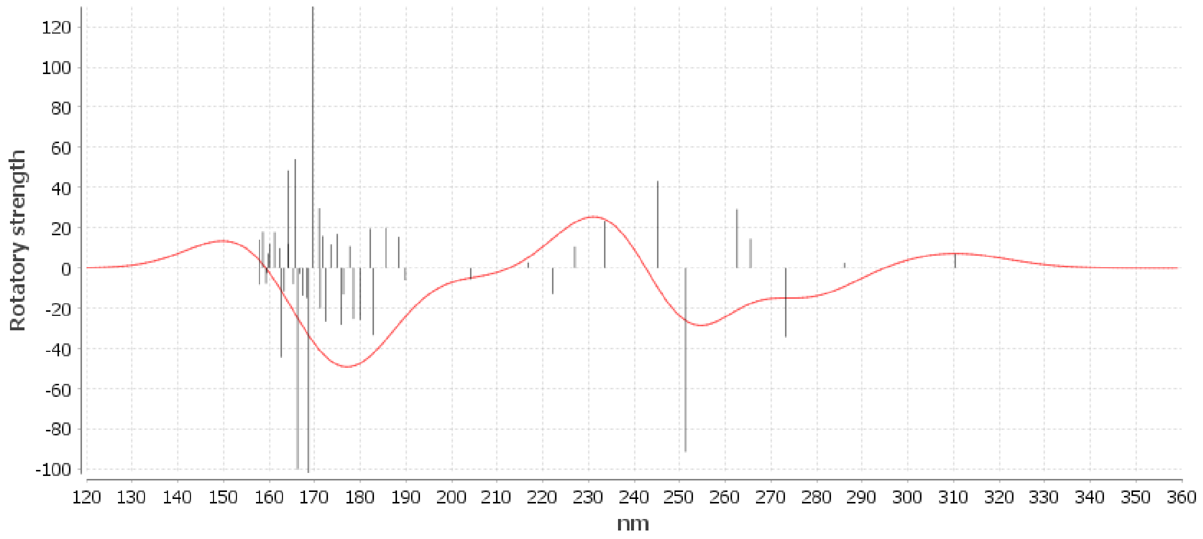

CAM-B3LYP/TZVP, conformer 2

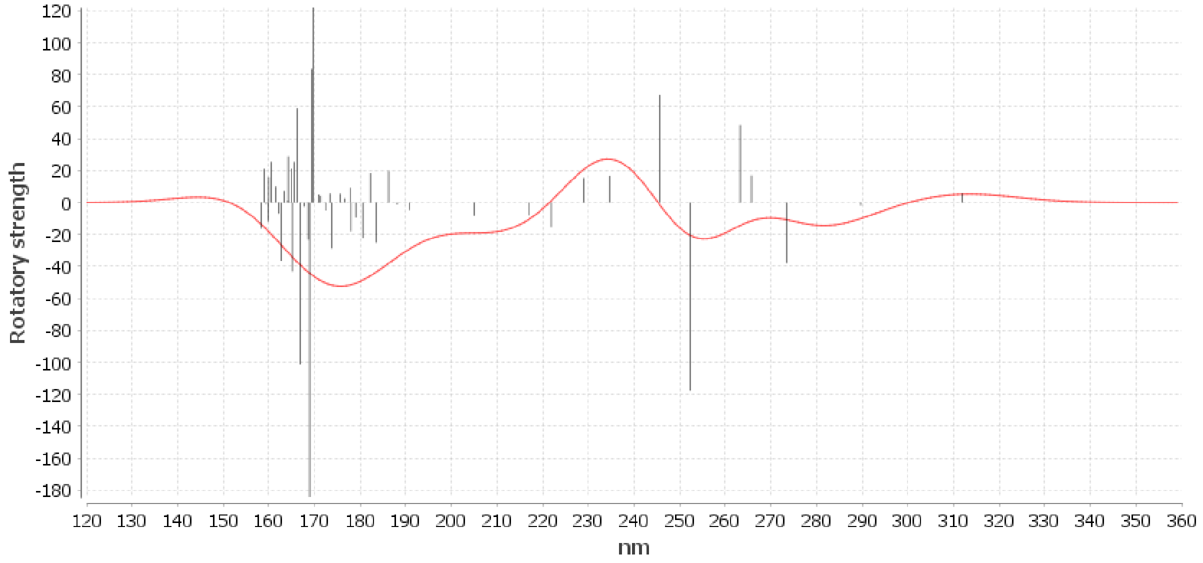

Calculations of enolate properties

Et-Z-enolate-imid-conf1

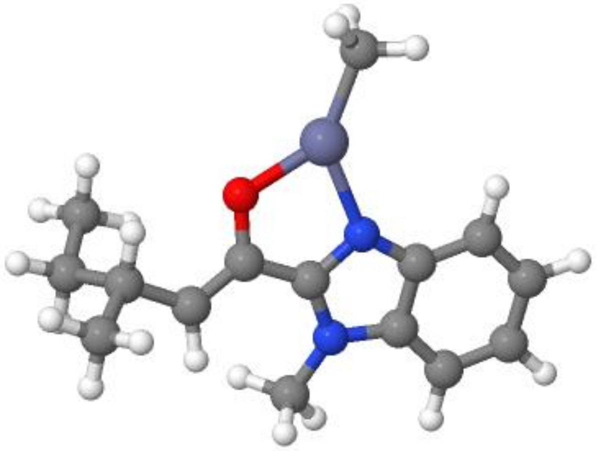

Energy (POTENTIAL) = -2547.588110965 Eh

	Atom	X	Y	Z
1	H	0.9953	3.7296	-1.6990
2	C	0.8516	3.8769	-0.6239
3	H	0.5806	4.9248	-0.4573
4	H	0.0044	3.2528	-0.3234
5	C	2.1106	3.5036	0.1472
6	H	2.9398	4.1531	-0.1685
7	H	1.9660	3.6900	1.2217
8	C	2.5443	2.0381	-0.0544
9	H	2.6094	1.8617	-1.1351
10	C	3.9198	1.7952	0.5708
11	H	4.6659	2.4768	0.1479
12	H	4.2582	0.7696	0.3950
13	H	3.8953	1.9621	1.6548
14	C	1.5341	1.0785	0.4999
15	H	1.5677	0.8923	1.5678
16	C	0.6665	0.4001	-0.2958
17	O	0.6351	0.5508	-1.6065
18	C	-0.3014	-0.6045	0.1767
19	N	-0.9063	-1.3658	-0.7431
20	C	-1.7545	-2.2178	-0.0723
21	C	-1.6586	-1.9542	1.3081
22	N	-0.7394	-0.9190	1.4356
23	C	-0.4301	-0.2374	2.6802
24	H	-0.3464	0.8365	2.5022
25	H	0.5043	-0.6025	3.1195
26	H	-1.2443	-0.4096	3.3863
27	Zn	-0.4247	-0.6795	-2.6280
28	C	-0.9013	-1.0941	-4.4639
29	H	-0.0417	-0.9643	-5.1290
30	H	-1.6906	-0.4250	-4.8232
31	H	-1.2612	-2.1207	-4.5821
32	C	-2.4046	-2.6629	2.2425
33	C	-3.2576	-3.6432	1.7499

34	C	-3.3617	-3.9078	0.3767
35	C	-2.6135	-3.2015	-0.5529
36	H	-2.3265	-2.4793	3.3095
37	H	-3.8555	-4.2210	2.4478
38	H	-4.0416	-4.6829	0.0366
39	H	-2.6873	-3.3995	-1.6181

Et-Z-enolate-Bn-imid

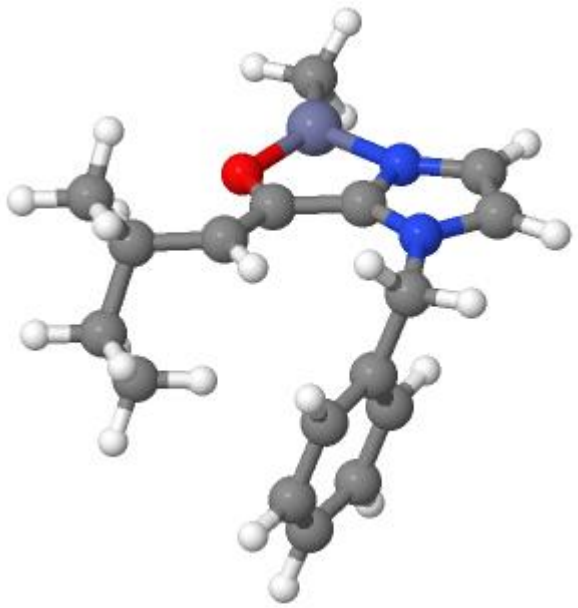

Energy (POTENTIAL) = -2624.964150169 Eh

	Atom	X	Y	Z
1	H	-1.8813	2.7354	0.2706
2	C	-1.3269	2.3396	1.1286
3	H	-1.8543	2.6389	2.0408
4	H	-1.3557	1.2480	1.0656
5	C	0.1091	2.8455	1.1265
6	H	0.1189	3.9408	1.2196
7	H	0.6418	2.4554	2.0072
8	C	0.8908	2.4567	-0.1417
9	H	0.3297	2.8173	-1.0120
10	C	2.2741	3.1161	-0.1441
11	H	2.1877	4.2058	-0.0631
12	H	2.8182	2.8834	-1.0642
13	H	2.8775	2.7658	0.7027
14	C	1.0050	0.9681	-0.2576
15	H	1.5424	0.4666	0.5376
16	C	0.4348	0.2567	-1.2596
17	O	-0.2428	0.8227	-2.2443
18	C	0.4874	-1.2105	-1.3906
19	N	-0.0415	-1.7722	-2.4885
20	C	0.0967	-3.1319	-2.3684
21	C	0.7106	-3.4104	-1.1853
22	N	0.9608	-2.1940	-0.5702
23	H	-0.2481	-3.8135	-3.1329
24	H	0.9902	-4.3465	-0.7240
25	C	1.4541	-2.0605	0.7951

26	H	1.8076	-3.0526	1.0980
27	H	2.3283	-1.4014	0.8067
28	Zn	-0.9414	-0.2906	-3.6271
29	C	-2.0088	-0.2037	-5.2473
30	H	-1.5138	-0.6865	-6.0958
31	H	-2.2023	0.8370	-5.5259
32	H	-2.9798	-0.6925	-5.1145
33	C	0.4007	-1.5667	1.7626
34	C	-1.5057	-0.6670	3.5958
35	C	0.7888	-0.8398	2.8862
36	C	-0.9504	-1.8434	1.5653
37	C	-1.8978	-1.3942	2.4776
38	C	-0.1581	-0.3917	3.7989
39	H	1.8401	-0.6015	3.0386
40	H	-1.2710	-2.3923	0.6833
41	H	-2.9499	-1.6027	2.3053
42	H	0.1568	0.1884	4.6616
43	H	-2.2492	-0.3043	4.2995

Et-Z-enolate-benzimid

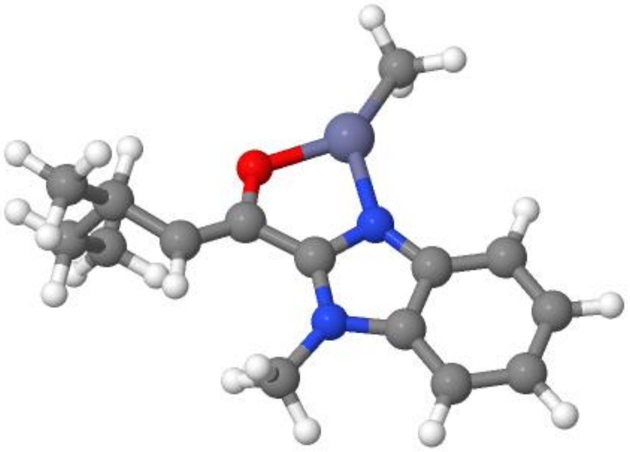

Energy (POTENTIAL) = -2547.588110965 Eh

	Atom	X	Y	Z
1	H	0.9953	3.7296	-1.6990
2	C	0.8516	3.8769	-0.6239
3	H	0.5806	4.9248	-0.4573
4	H	0.0044	3.2528	-0.3234
5	C	2.1106	3.5036	0.1472
6	H	2.9398	4.1531	-0.1685
7	H	1.9660	3.6900	1.2217
8	C	2.5443	2.0381	-0.0544
9	H	2.6094	1.8617	-1.1351
10	C	3.9198	1.7952	0.5708
11	H	4.6659	2.4768	0.1479
12	H	4.2582	0.7696	0.3950
13	H	3.8953	1.9621	1.6548
14	C	1.5341	1.0785	0.4999
15	H	1.5677	0.8923	1.5678
16	C	0.6665	0.4001	-0.2958
17	O	0.6351	0.5508	-1.6065
18	C	-0.3014	-0.6045	0.1767

19	N	-0.9063	-1.3658	-0.7431
20	C	-1.7545	-2.2178	-0.0723
21	C	-1.6586	-1.9542	1.3081
22	N	-0.7394	-0.9190	1.4356
23	C	-0.4301	-0.2374	2.6802
24	H	-0.3464	0.8365	2.5022
25	H	0.5043	-0.6025	3.1195
26	H	-1.2443	-0.4096	3.3863
27	Zn	-0.4247	-0.6795	-2.6280
28	C	-0.9013	-1.0941	-4.4639
29	H	-0.0417	-0.9643	-5.1290
30	H	-1.6906	-0.4250	-4.8232
31	H	-1.2612	-2.1207	-4.5821
32	C	-2.4046	-2.6629	2.2425
33	C	-3.2576	-3.6432	1.7499
34	C	-3.3617	-3.9078	0.3767
35	C	-2.6135	-3.2015	-0.5529
36	H	-2.3265	-2.4793	3.3095
37	H	-3.8555	-4.2210	2.4478
38	H	-4.0416	-4.6829	0.0366
39	H	-2.6873	-3.3995	-1.6181

Ph-Z-enolate-imid

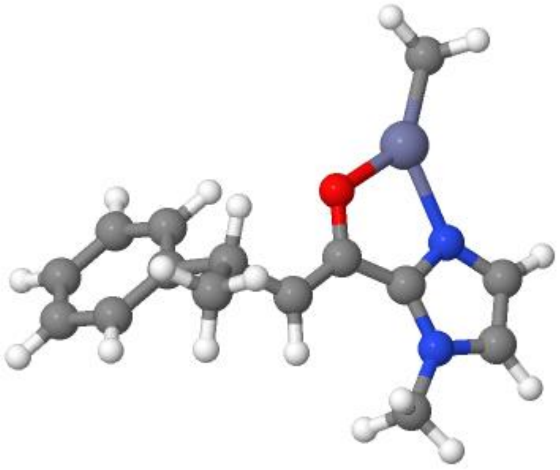

Energy (POTENTIAL) = -2546.364526575 Eh

	Atom	X	Y	Z
1	C	1.7517	0.8465	0.1662
2	H	1.6582	1.2001	-0.8672
3	C	3.2283	0.5677	0.4434
4	H	3.8193	1.4885	0.4064
5	H	3.6213	-0.1233	-0.3078
6	H	3.3816	0.1062	1.4254
7	C	0.9399	-0.4195	0.2642
8	H	1.1075	-1.0405	1.1353
9	C	0.0063	-0.7430	-0.6661
10	O	-0.2807	0.0431	-1.6894
11	C	-0.8076	-1.9705	-0.6648
12	N	-1.7615	-2.1084	-1.5989
13	C	-2.3362	-3.3407	-1.4232
14	C	-1.7284	-3.9664	-0.3766

15	N	-0.7610	-3.0976	0.0982
16	H	-3.1389	-3.6955	-2.0538
17	H	-1.8891	-4.9368	0.0697
18	C	0.1560	-3.3957	1.1866
19	H	1.1907	-3.2735	0.8542
20	H	0.0024	-4.4294	1.4993
21	H	-0.0233	-2.7375	2.0414
22	Zn	-1.7232	-0.4623	-2.8483
23	C	-2.6993	0.2068	-4.3876
24	H	-3.7138	0.5150	-4.1138
25	H	-2.7886	-0.5435	-5.1794
26	H	-2.1961	1.0801	-4.8141
27	C	1.1494	1.9345	1.0469
28	C	-0.0479	3.9538	2.5914
29	C	1.7075	2.3301	2.2624
30	C	-0.0242	2.5664	0.6193
31	C	-0.6157	3.5645	1.3804
32	C	1.1138	3.3313	3.0285
33	H	2.6241	1.8698	2.6193
34	H	-0.4670	2.2583	-0.3249
35	H	-1.5201	4.0508	1.0233
36	H	1.5699	3.6296	3.9693
37	H	-0.5054	4.7411	3.1848

Ph-E-enolate-imid

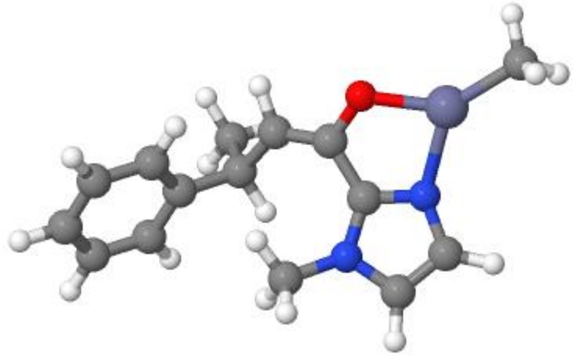

Energy (POTENTIAL) = -2546.356007402 Eh

	Atom	X	Y	Z
1	C	-0.4358	1.4165	0.4731
2	H	0.6563	1.3216	0.5407
3	C	-0.8689	2.3686	1.6089
4	H	-0.4070	3.3548	1.5021
5	H	-0.5929	1.9508	2.5812
6	H	-1.9551	2.5032	1.5903
7	C	-1.0744	0.0829	0.7038
8	C	-0.5075	-1.1291	0.9103
9	O	-1.2034	-2.1518	1.3850
10	C	0.9125	-1.4933	0.7202
11	N	1.4172	-2.4968	1.4539
12	C	2.6999	-2.7108	1.0186
13	C	2.9761	-1.8457	0.0014
14	N	1.8355	-1.0850	-0.1915
15	H	3.3343	-3.4694	1.4552

16	H	3.8565	-1.7148	-0.6109
17	C	1.6134	-0.2200	-1.3455
18	H	0.5417	-0.1138	-1.5153
19	H	2.0707	-0.6794	-2.2250
20	H	2.0425	0.7747	-1.1979
21	Zn	-0.2239	-3.5658	2.2123
22	C	-0.5002	-5.2238	3.1842
23	H	-1.5062	-5.2462	3.6154
24	H	0.2159	-5.3559	4.0008
25	H	-0.4104	-6.0922	2.5232
26	C	-0.7715	2.0575	-0.8672
27	C	-1.3908	3.3329	-3.2918
28	C	-1.8362	1.6183	-1.6534
29	C	-0.0182	3.1434	-1.3214
30	C	-0.3186	3.7741	-2.5216
31	C	-2.1468	2.2535	-2.8526
32	H	-2.4206	0.7613	-1.3302
33	H	0.8197	3.4987	-0.7226
34	H	0.2847	4.6135	-2.8577
35	H	-2.9846	1.8981	-3.4471
36	H	-1.6317	3.8265	-4.2293
37	H	-2.1421	0.1039	0.9239

Et-E-enolate-imid

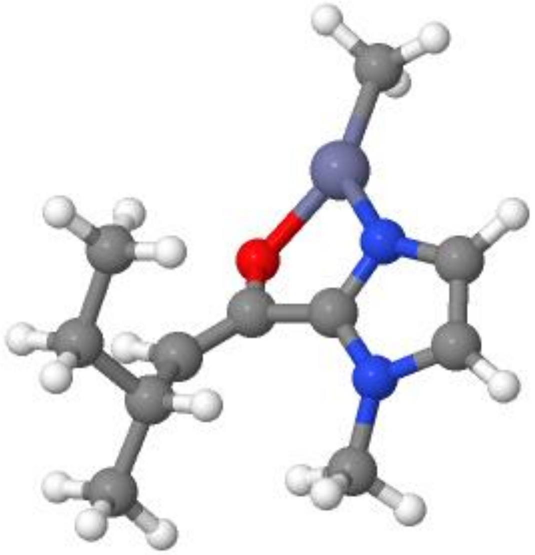

Energy (POTENTIAL) = -2393.962428901 Eh

	Atom	X	Y	Z
1	C	-1.5053	1.4897	-0.2803
2	H	-0.4879	1.8960	-0.2002
3	C	-2.4688	2.3651	0.5266
4	H	-2.4651	3.4010	0.1697
5	H	-2.2253	2.3667	1.5936
6	H	-3.4915	1.9829	0.4307
7	C	-1.5317	0.0647	0.1760
8	C	-0.5033	-0.7771	0.4315
9	O	-0.6571	-2.0948	0.4963
10	C	0.9133	-0.4016	0.6118

11	N	1.8620	-1.2806	0.2597
12	C	3.0665	-0.7527	0.6511
13	C	2.8522	0.4480	1.2609
14	N	1.4836	0.6605	1.2417
15	H	4.0015	-1.2664	0.4770
16	H	3.5308	1.1524	1.7198
17	C	0.7964	1.6845	2.0180
18	H	0.7964	2.6513	1.5069
19	H	1.2910	1.7904	2.9870
20	H	-0.2347	1.3653	2.1753
21	Zn	0.8861	-3.1404	0.0850
22	C	1.4814	-4.9559	-0.2639
23	H	1.2264	-5.6167	0.5712
24	H	2.5623	-5.0178	-0.4211
25	H	0.9914	-5.3614	-1.1551
26	H	-2.5013	-0.4304	0.0910
27	C	-1.8778	1.5508	-1.7783
28	H	-2.9032	1.1727	-1.8971
29	H	-1.8996	2.6050	-2.0885
30	C	-0.9328	0.7644	-2.6762
31	H	-0.9566	-0.3016	-2.4343
32	H	0.1023	1.1057	-2.5552
33	H	-1.2019	0.8802	-3.7308

Z-Me-TMS-benzoxazol

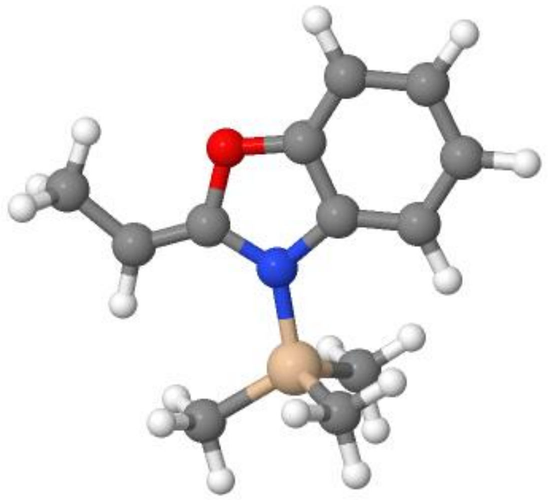

Energy (POTENTIAL) = -886.8360538115 Eh

	Atom	X	Y	Z
1	C	-0.6890	-0.0001	-3.9403
2	C	-0.9617	-0.0001	-2.4688
3	C	-0.0035	-0.0000	-1.5335
4	O	1.3344	0.0001	-1.9023
5	N	-0.0639	-0.0001	-0.1229
6	Si	-1.4640	-0.0000	0.9785
7	C	-1.3579	-1.5639	2.0056
8	C	-1.3576	1.5638	2.0056
9	C	-3.1002	0.0001	0.0705
10	C	3.2256	0.0001	1.7055
11	C	2.0949	0.0001	-0.7704

12	C	1.8268	-0.0001	1.6133
13	C	4.0331	0.0000	0.5759
14	C	3.4669	0.0002	-0.7048
15	C	1.2610	-0.0001	0.3457
16	H	-3.2520	-0.8881	-0.5484
17	H	-3.2518	0.8883	-0.5484
18	H	-1.4950	2.4400	1.3627
19	H	-0.3993	1.6866	2.5159
20	H	3.6837	0.0004	2.6898
21	H	5.1135	-0.0003	0.6801
22	H	4.0702	0.0004	-1.6066
23	H	0.3803	0.0001	-4.1564
24	H	-1.9927	-0.0002	-2.1488
25	H	-2.1447	1.5868	2.7673
26	H	1.2273	-0.0002	2.5164
27	H	-2.1450	-1.5868	2.7672
28	H	-3.8912	0.0002	0.8308
29	H	-1.4953	-2.4400	1.3626
30	H	-0.3995	-1.6868	2.5159
31	H	-1.1265	-0.8804	-4.4290
32	H	-1.1268	0.8801	-4.4290

Z-Me-TMS-ketene-aminal

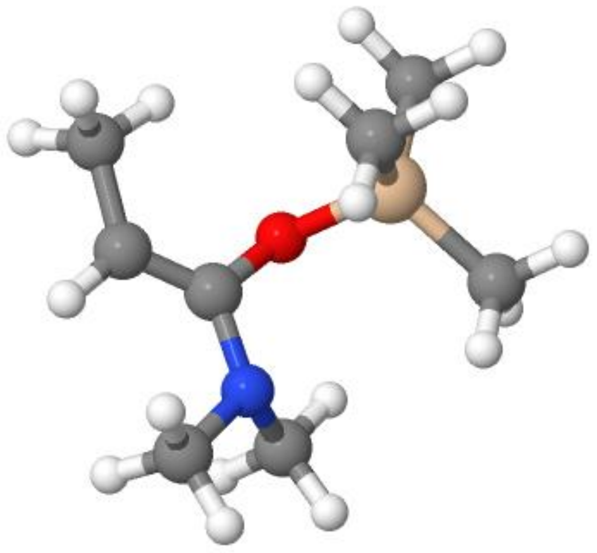

Energy (POTENTIAL) = -735.6419055809 Eh

	Atom	X	Y	Z
1	C	-1.7412	1.3412	0.3963
2	C	-0.7609	0.4680	0.6745
3	O	-0.5767	-0.6401	-0.1020
4	N	0.1701	0.5556	1.7232
5	C	0.1233	1.7667	2.5230
6	C	0.2776	-0.6492	2.5530
7	Si	0.6668	-0.8966	-1.2013
8	C	-0.0378	-2.1439	-2.4056
9	C	2.1752	-1.6239	-0.3572
10	C	1.0824	0.7243	-2.0365
11	C	-2.6179	1.2531	-0.8118
12	H	-1.9275	2.1555	1.0880

13	H	-3.6763	1.1929	-0.5290
14	H	-2.5152	2.1345	-1.4588
15	H	-2.3875	0.3670	-1.4067
16	H	2.5738	-0.9495	0.4069
17	H	2.9728	-1.8117	-1.0854
18	H	1.4468	1.4618	-1.3145
19	H	1.8584	0.5901	-2.7983
20	H	1.9457	-2.5807	0.1245
21	H	-0.7968	1.8506	3.1298
22	H	0.1918	2.6451	1.8758
23	H	0.9781	1.7730	3.2063
24	H	1.2068	-0.6139	3.1308
25	H	-0.5680	-0.7430	3.2574
26	H	-0.8956	-1.7388	-2.9520
27	H	0.2020	1.1507	-2.5283
28	H	0.7096	-2.4548	-3.1440
29	H	-0.3776	-3.0448	-1.8834
30	H	0.2975	-1.5394	1.9252

Z-Me-TMS-ketene-acetal

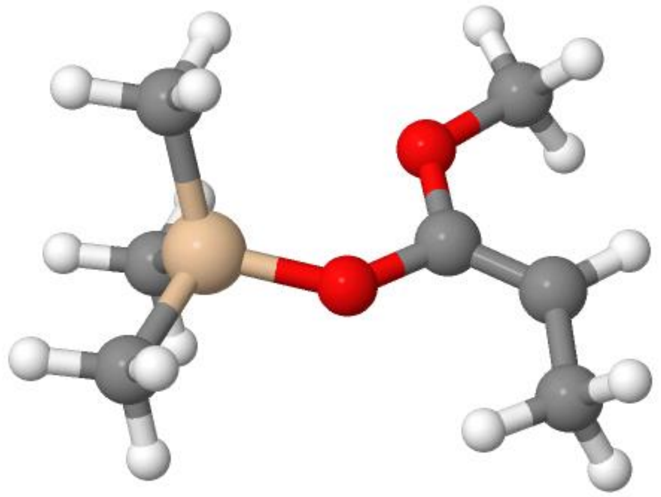

Energy (POTENTIAL) = -716.2253846488 Eh

	Atom	X	Y	Z
1	C	-0.8391	-0.0000	2.3653
2	C	-0.0666	-0.0000	1.2724
3	O	-0.5800	-0.0000	0.0345
4	O	1.2943	0.0000	1.2320
5	C	2.0026	0.0000	2.4617
6	Si	0.0305	-0.0000	-1.5307
7	C	-1.5205	-0.0000	-2.5813
8	C	1.0329	-1.5399	-1.8878
9	C	1.0329	1.5399	-1.8878
10	C	-2.3328	-0.0000	2.3034
11	H	-0.3652	0.0000	3.3395
12	H	-2.7573	-0.8817	2.7996
13	H	-2.7573	0.8817	2.7996
14	H	-2.6811	-0.0000	1.2690
15	H	1.9604	-1.5751	-1.3104
16	H	1.3004	-1.5715	-2.9512
17	H	1.9605	1.5751	-1.3104

18	H	1.3004	1.5715	-2.9512
19	H	0.4628	-2.4487	-1.6690
20	H	1.7725	-0.8957	3.0513
21	H	1.7725	0.8958	3.0513
22	H	3.0612	0.0000	2.1971
23	H	-2.1385	0.8831	-2.3898
24	H	0.4629	2.4487	-1.6690
25	H	-1.2700	-0.0000	-3.6484
26	H	-2.1385	-0.8831	-2.3898

RR-Me-C7-benzodithiolyl-conf3

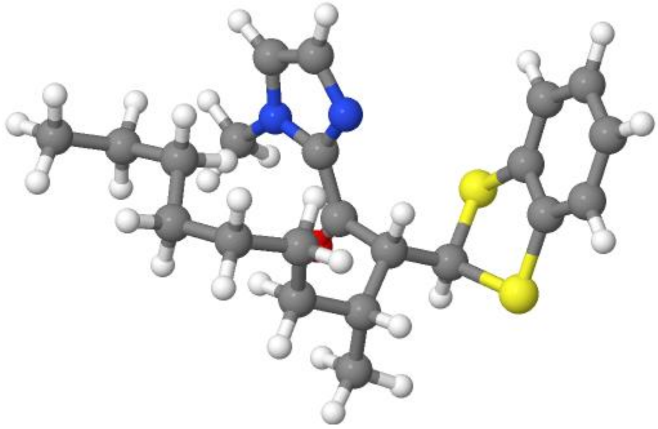

Energy (POTENTIAL) = -1835.832379765 Eh

	Atom	X	Y	Z
1	C	0.6501	-0.6081	-1.4224
2	O	0.3900	-0.8385	-2.5957
3	C	0.9371	0.7555	-0.9547
4	N	1.2941	1.0863	0.2852
5	C	1.4825	2.4277	0.2844
6	C	1.2355	2.9382	-0.9840
7	N	0.8923	1.8719	-1.7667
8	H	1.7795	2.9716	1.1817
9	H	1.2708	3.9508	-1.3823
10	C	0.5184	1.9397	-3.1729
11	H	1.1736	1.2955	-3.7763
12	H	0.6075	2.9866	-3.5021
13	H	-0.5186	1.5971	-3.3095
14	C	0.7140	-1.7167	-0.3725
15	H	0.9779	-1.2510	0.5870
16	C	-0.6544	-2.4412	-0.2059
17	H	-0.5394	-3.0709	0.6950
18	C	-1.8006	-1.4481	0.0654
19	H	-2.7413	-2.0288	0.1267
20	H	-1.9181	-0.7904	-0.8163
21	C	-1.0135	-3.3611	-1.3811
22	H	-1.0447	-2.8030	-2.3313
23	H	-2.0043	-3.8195	-1.2111
24	H	-0.2912	-4.1884	-1.4953
25	C	-1.6651	-0.6076	1.3391
26	H	-1.4949	-1.2868	2.1965
27	H	-0.7695	0.0335	1.2833
28	C	-2.9033	0.2549	1.6382

29	H	-2.7111	0.8395	2.5586
30	H	-3.7586	-0.4093	1.8689
31	C	-3.3277	1.2173	0.5164
32	H	-3.6364	0.6383	-0.3756
33	H	-4.2328	1.7587	0.8488
34	C	-2.2433	2.2200	0.1082
35	H	-1.3176	1.6724	-0.1301
36	H	-1.9967	2.8653	0.9743
37	C	-2.6021	3.0978	-1.0982
38	H	-1.7193	3.7137	-1.3560
39	H	-2.7854	2.4451	-1.9753
40	C	-3.8090	4.0162	-0.8885
41	H	-3.6616	4.6677	-0.0067
42	H	-4.7384	3.4418	-0.7251
43	H	-3.9742	4.6690	-1.7649
44	C	1.8704	-2.6601	-0.7490
45	S	3.4609	-1.7430	-0.9252
46	C	3.8250	-1.8427	0.8070
47	C	3.2099	-2.9074	1.4984
48	S	2.1576	-3.9555	0.5238
49	H	1.6934	-3.1606	-1.7098
50	C	3.4662	-3.1012	2.8571
51	C	4.3464	-2.2391	3.5235
52	C	4.9503	-1.1798	2.8393
53	C	4.6845	-0.9729	1.4801
54	H	2.9765	-3.9180	3.3948
55	H	4.5470	-2.3908	4.5882
56	H	5.6233	-0.4981	3.3678
57	H	5.1384	-0.1334	0.9466

RR-Me-C7-benzodithiolyl-conf2

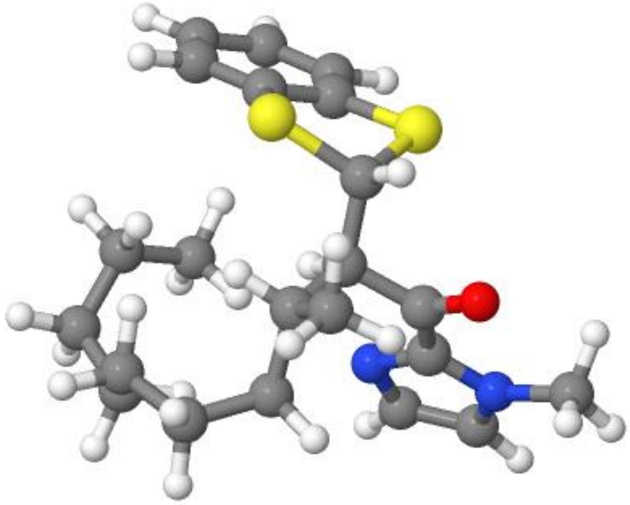

Energy (POTENTIAL) = -1835.833804445 Eh

	Atom	X	Y	Z
1	C	0.6376	-2.0494	-1.3158
2	O	0.6383	-3.2440	-1.5830
3	C	1.3716	-1.0795	-2.1398
4	N	1.3981	0.2381	-1.9475
5	C	2.1876	0.7526	-2.9215

6	C	2.6625	-0.2707	-3.7316
7	N	2.1397	-1.4306	-3.2322
8	H	2.3846	1.8221	-3.0018
9	H	3.3190	-0.2668	-4.6006
10	C	2.3815	-2.7660	-3.7634
11	H	2.8259	-3.4110	-2.9909
12	H	3.0695	-2.6765	-4.6186
13	H	1.4379	-3.2290	-4.0884
14	C	-0.1189	-1.5034	-0.1093
15	H	0.2302	-0.4796	0.0862
16	C	-1.6493	-1.4211	-0.3818
17	H	-2.0567	-0.9820	0.5437
18	C	-1.9648	-0.4522	-1.5455
19	H	-2.0740	-1.0323	-2.4815
20	H	-1.1119	0.2243	-1.7104
21	C	-2.3287	-2.7800	-0.5814
22	H	-1.8907	-3.3328	-1.4298
23	H	-3.4052	-2.6347	-0.7827
24	H	-2.2493	-3.4213	0.3142
25	C	-3.2278	0.3913	-1.3090
26	H	-3.4984	0.9095	-2.2492
27	H	-4.0783	-0.2755	-1.0703
28	C	-3.0674	1.4313	-0.1886
29	H	-2.7380	0.9184	0.7337
30	H	-4.0562	1.8646	0.0540
31	C	-2.0939	2.5736	-0.5173
32	H	-1.2050	2.1782	-1.0389
33	H	-2.5825	3.2617	-1.2334
34	C	-1.6344	3.3596	0.7211
35	H	-2.5228	3.6296	1.3257
36	H	-1.1812	4.3167	0.3979
37	C	-0.6206	2.6162	1.6078
38	H	-0.4966	3.1788	2.5526
39	H	-1.0258	1.6300	1.9029
40	C	0.7542	2.4217	0.9615
41	H	1.1933	3.3966	0.6741
42	H	0.7143	1.7950	0.0538
43	H	1.4506	1.9332	1.6638
44	C	0.2414	-2.3657	1.1094
45	S	2.0613	-2.3581	1.4206
46	C	2.0020	-0.9761	2.5278
47	C	0.7512	-0.7146	3.1223
48	S	-0.5725	-1.8005	2.6584
49	H	-0.0364	-3.4153	0.9494
50	C	0.6170	0.3196	4.0507
51	C	1.7356	1.0871	4.3959
52	C	2.9764	0.8312	3.8041
53	C	3.1119	-0.1966	2.8631
54	H	-0.3583	0.5312	4.4971
55	H	1.6291	1.8997	5.1204
56	H	3.8452	1.4431	4.0637
57	H	4.0780	-0.3896	2.3883

SR-Me-C7-benzodithiolyl-conf2

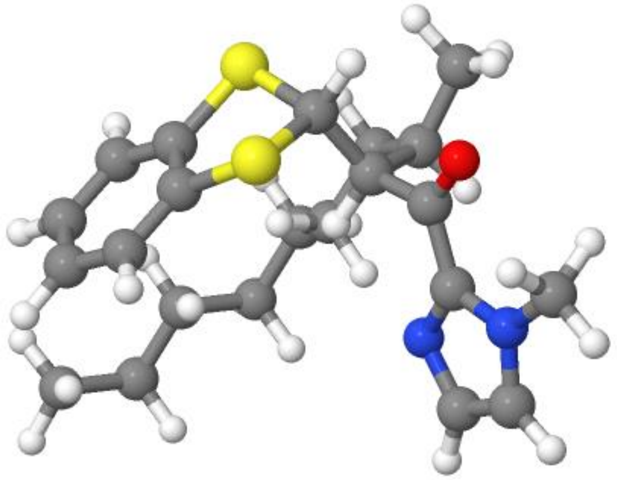

Energy (POTENTIAL) = -1835.831360044 Eh

	Atom	X	Y	Z
1	C	0.8281	2.7297	-1.4600
2	O	0.9209	3.9484	-1.5034
3	C	1.6451	1.8619	-2.3216
4	N	1.5952	0.5316	-2.3373
5	C	2.4991	0.1279	-3.2636
6	C	3.1214	1.2330	-3.8294
7	N	2.5731	2.3319	-3.2290
8	H	2.6696	-0.9261	-3.4864
9	H	3.8947	1.3237	-4.5908
10	C	2.9325	3.7170	-3.5031
11	H	2.0500	4.2871	-3.8288
12	H	3.6969	3.7245	-4.2959
13	H	3.3298	4.1980	-2.5964
14	C	-0.1661	2.0146	-0.5477
15	C	-1.4924	1.8220	-1.3536
16	H	-1.1576	1.5630	-2.3753
17	C	-2.3632	0.6325	-0.8914
18	H	-3.1088	0.4556	-1.6913
19	H	-2.9362	0.9129	0.0085
20	C	-2.3159	3.1110	-1.4489
21	H	-2.7447	3.3872	-0.4684
22	H	-3.1610	2.9756	-2.1475
23	H	-1.7075	3.9603	-1.8054
24	C	-1.6014	-0.6664	-0.6183
25	H	-0.9053	-0.8753	-1.4529
26	H	-0.9699	-0.5316	0.2761
27	C	-2.5192	-1.8729	-0.3990
28	H	-3.1795	-1.9917	-1.2800
29	H	-3.1900	-1.6702	0.4598
30	C	-1.7694	-3.1907	-0.1593
31	H	-2.5009	-4.0220	-0.1325
32	H	-1.1087	-3.3921	-1.0257
33	C	-0.9351	-3.2253	1.1255
34	H	-1.5927	-3.0098	1.9911
35	H	-0.1838	-2.4146	1.1145
36	C	-0.2183	-4.5600	1.3509

37	H	-0.9637	-5.3795	1.3708
38	H	0.4374	-4.7677	0.4828
39	C	0.6135	-4.5927	2.6349
40	H	-0.0234	-4.4393	3.5263
41	H	1.3772	-3.7929	2.6350
42	H	1.1340	-5.5599	2.7576
43	C	-0.2453	2.7961	0.7682
44	S	-1.4408	2.1359	1.9945
45	C	-0.2941	0.9530	2.6498
46	C	1.0701	1.2593	2.4601
47	S	1.4082	2.7742	1.6005
48	H	-0.4935	3.8511	0.5962
49	C	2.0529	0.4050	2.9657
50	C	1.6748	-0.7429	3.6744
51	C	0.3226	-1.0427	3.8634
52	C	-0.6670	-0.1998	3.3433
53	H	3.1102	0.6321	2.8026
54	H	2.4448	-1.4135	4.0669
55	H	0.0317	-1.9507	4.3981
56	H	-1.7254	-0.4457	3.4662
57	H	0.2467	1.0201	-0.3414

SR-Me-C7-benzodithiolyl-conf3

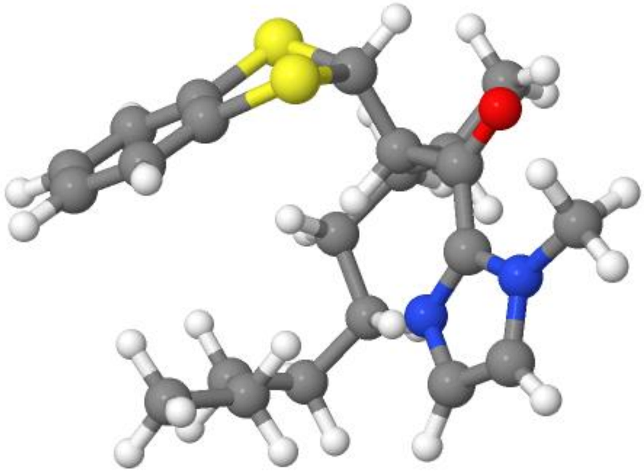

Energy (POTENTIAL) = -1835.834404836 Eh

	Atom	X	Y	Z
1	C	0.2822	2.4531	-0.9490
2	O	0.4073	3.6700	-0.9426
3	C	0.8360	1.6347	-2.0355
4	N	0.7385	0.3114	-2.1269
5	C	1.3877	-0.0460	-3.2620
6	C	1.9000	1.0850	-3.8843
7	N	1.5451	2.1494	-3.1025
8	H	1.4645	-1.0863	-3.5796
9	H	2.4795	1.2147	-4.7972
10	C	1.8816	3.5430	-3.3634
11	H	0.9682	4.1471	-3.4697
12	H	2.4703	3.5868	-4.2929
13	H	2.4660	3.9591	-2.5293
14	C	-0.4808	1.7110	0.1512

15	C	-1.9957	1.7150	-0.2358
16	H	-2.0045	1.4472	-1.3088
17	C	-2.8651	0.6605	0.4873
18	H	-3.8395	0.6454	-0.0396
19	H	-3.0756	1.0101	1.5110
20	C	-2.6378	3.1016	-0.0971
21	H	-2.7212	3.3976	0.9650
22	H	-3.6614	3.0897	-0.5120
23	H	-2.0610	3.8818	-0.6208
24	C	-2.3269	-0.7723	0.5620
25	H	-1.3886	-0.7875	1.1435
26	H	-3.0482	-1.3691	1.1544
27	C	-2.1198	-1.4542	-0.7944
28	H	-1.3107	-0.9489	-1.3505
29	H	-3.0395	-1.3256	-1.3987
30	C	-1.8190	-2.9576	-0.6879
31	H	-2.6776	-3.4584	-0.1983
32	H	-1.7600	-3.3813	-1.7097
33	C	-0.5349	-3.3286	0.0689
34	H	-0.4757	-4.4329	0.1354
35	H	-0.5912	-2.9681	1.1143
36	C	0.7514	-2.7996	-0.5742
37	H	0.7728	-3.1074	-1.6394
38	H	0.7427	-1.6963	-0.5865
39	C	2.0180	-3.2910	0.1276
40	H	2.0892	-4.3954	0.1075
41	H	2.0298	-2.9718	1.1847
42	H	2.9281	-2.8857	-0.3518
43	C	-0.0876	2.3589	1.4859
44	S	-0.8535	1.6200	2.9793
45	C	0.3789	0.3555	3.1339
46	C	1.6271	0.6492	2.5465
47	S	1.7447	2.2184	1.7296
48	H	-0.3173	3.4319	1.4923
49	C	2.6831	-0.2570	2.6632
50	C	2.4966	-1.4482	3.3756
51	C	1.2551	-1.7439	3.9466
52	C	0.1881	-0.8459	3.8195
53	H	3.6467	-0.0374	2.1952
54	H	3.3247	-2.1570	3.4660
55	H	1.1083	-2.6850	4.4848
56	H	-0.7887	-1.0791	4.2522
57	H	-0.1332	0.6697	0.1532

Energies and enolate properties

Geometry: r2-SCAN-3c/def2-mTZVP

SP energy and property calculations: wB97X-D4/def2-TZVPPD

Name	Energy (Hartree)	HOMO energy (Hartree)	HOMO energy (eV)	NBO charge at C-2
Et-Z-enolate-imid	-2394.29790888442	-0.2855	-7.77	-0.275
Et-E-enolate-imid	-2394.28636376604	-0.2861	-7.79	-0.287
Ph-Z-enolate-imid	-2546.72904419517	-0.2871	-7.81	-0.261
Ph-E-enolate-imid	-2546.71972031304	-0.2894	-7.88	-0.291
Et-Z-enolate-benzimid	-2547.95816848451	-0.2899	-7.89	-0.254
Et-Z-enolate-Bn-imid	-2625.36949727832	-0.2816	-7.66	-0.252
Z-Me-TMS-benzoxazol	-887.11236124528	-0.2621	-7.13	-0.368
Z-Me-TMS-ketene-aminal	-735.89437601138	-0.2894	-7.87	-0.343
Z-Me-TMS-ketene-acetal	-716.45213101026	-0.2968	-8.08	-0.396

8. References

1. Armarego, W. L. F.; Chai, C. L. L. *Purification of Laboratory Chemicals (6th Edition)*; Elsevier: Online version available at www.knovel.com, 2009.
2. Drissi-Amraoui, S.; Schmid, T. E.; Lauberteaux, J.; Crévisy, C.; Baslé, O.; de Figueiredo, R. M.; Halbert, S.; Gérard, H.; Mauduit, M.; Campagne, J.-M. *Adv. Synth. Catal.* **2016**, *358*, 2519-2540.
3. Drissi-Amraoui, S.; Morin, M. S. T.; Crévisy, C.; Baslé, O.; Marcia de Figueiredo, R.; Mauduit, M.; Campagne, J.-M. *Angew. Chem. Int. Ed.* **2015**, *54*, 11830-11834.
4. Drusan, M.; Rakovský, E.; Marek, J.; Šebesta, R. *Adv. Synth. Catal.* **2015**, *357*, 1493-1498.
5. Vargová, D.; Perez, J. M.; Harutyunyan, S. R.; Šebesta, R. *Chem. Commun.* **2019**, *55*, 11766-11769.
6. Mudráková, B.; Kisszékelyi, P.; Vargová, D.; Zakiewicz, D.; Šebesta, R. *Adv. Synth. Catal.* **2022**, *364*, 1337-1344.
7. Shao, Y.; Molnar, L. F.; Jung, Y.; Kussmann, J.; Ochsenfeld, C.; Brown, S. T.; Gilbert, A. T. B.; Slipchenko, L. V.; Levchenko, S. V.; ONeill, D. P.; Jr, R. A. D.; Lochan, R. C.; Wang, T.; Beran, G. J. O.; Besley, N. A.; Herbert, J. M.; Lin, C. Y.; Voorhis, T. V.; Chien, S. H.; Sodt, A.; Steele, R. P.; Rassolov, V. A.; Maslen, P. E.; Korambath, P. P.; Adamson, R. D.; Austin, B.; Baker, J.; Byrd, E. F. C.; Dachsel, H.; Doerksen, R. J.; Dreuw, A.; Dunietz, B. D.; Dutoi, A. D.; Furlani, T. R.; Gwaltney, S. R.; Heyden, A.; Hirata, S.; Hsu, C.-P.; Kedziora, G.; Khalliulin, R. Z.; Klunzinger, P.; Lee, A. M.; Lee, M. S.; Liang, W.; Lotan, I.; Nair, N.; Peters, B.; Proynov, E. I.; Pieniazek, P. A.; Rhee, Y. M.; Ritchie, J.; Rosta, E.; Sherrill, C. D.; Simmonett, A. C.; Subotnik, J. E.; Woodcock III, H. L.; Zhang, W.; Bell, A. T.; Chakraborty, A. K. *Phys. Chem. Chem. Phys.* **2006**, *8*, 3172 - 3191.
8. TURBOMOLE V7.5, TURBOMOLE GmbH, Karlsruhe, 2019.
9. Balasubramani, S. G.; Chen, G. P.; Coriani, S.; Diedenhofen, M.; Frank, M. S.; Franzke, Y. J.; Furche, F.; Grotjahn, R.; Harding, M. E.; Hättig, C.; Hellweg, A.; Helmich-Paris, B.; Holzer, C.; Huniar, U.; Kaupp, M.; Marefat Khah, A.; Karbalaei Khani, S.; Müller, T.; Mack, F.; Nguyen, B. D.; Parker, S. M.; Perlt, E.; Rappoport, D.; Reiter, K.; Roy, S.; Rückert, M.; Schmitz, G.; Sierka, M.; Tapavicza, E.; Tew, D. P.; van Wüllen, C.; Voora, V. K.; Weigend, F.; Wodyński, A.; Yu, J. M. *J. Chem. Phys.* **2020**, *152*, 184107.
10. Becke, A. D. *J. Chem. Phys.* **1993**, *98*, 5648-5652.
11. Lee, C.; Yang, W.; R.G. Parr *Phys. Rev. B* **1988**, *37*, 785-789.

12. Vosko, S. H.; Wilk, L.; Nusair, M. *Can. J. Phys.* **1980**, *58*, 1200-1211.
13. Stephens, P. J.; Devlin, F. J.; Chabalowski, C. F.; Frisch, M. J. *J. Phys. Chem.* **1994**, *98*, 11623-11627.
14. Grimme, S.; Ehrlich, S.; Goerigk, L. *J. Comput. Chem.* **2011**, *32*, 1456-1465.
15. Grimme, S.; Brandenburg, J. G.; Bannwarth, C.; Hansen, A. *J. Chem. Phys.* **2015**, *143*, 054107.
16. Schäfer, A.; Horn, H.; Ahlrichs, R. *J. Chem. Phys.* **1992**, *97*, 2571-2577.
17. Zhao, Y.; Truhlar, D. *Theor. Chem. Acc.* **2008**, *120*, 215-241.
18. Zhao, Y.; Truhlar, D. G. *Theor. Chem. Acc.* **2008**, *120*, 215-241.
19. Weigend, F.; Ahlrichs, R. *Phys. Chem. Chem. Phys.* **2005**, *7*, 3297-3305.
20. Chai, J.-D.; Head-Gordon, M. *Phys. Chem. Chem. Phys.* **2008**, *10*, 6615-6620.
21. Yanai, T.; Tew, D. P.; Handy, N. C. *Chem. Phys. Lett.* **2004**, *393*, 51-57.
22. Brandenburg, J. G.; Bannwarth, C.; Hansen, A.; Grimme, S. *J. Chem. Phys.* **2018**, *148*, 064104.
23. Ernzerhof, M.; Scuseria, G. E. *J. Chem. Phys.* **1999**, *110*, 5029-5036.
24. Caldeweyher, E.; Ehlert, S.; Hansen, A.; Neugebauer, H.; Spicher, S.; Bannwarth, C.; Grimme, S. *J. Chem. Phys.* **2019**, *150*.
25. Kühn, M.; Weigend, F. *J. Chem. Theory Comput.* **2013**, *9*, 5341-5348.
26. Kühn, M.; Weigend, F. *J. Chem. Phys.* **2015**, *142*, 034116.
